# High-resolution multimodal profiling of human epileptic brain activity via explanted depth electrodes

**DOI:** 10.1172/jci.insight.184518

**Published:** 2025-01-09

**Authors:** Anuj Kumar Dwivedi, Arun Mahesh, Albert Sanfeliu, Julian Larkin, Rebecca A. Siwicki, Kieron J. Sweeney, Donncha F. O’Brien, Peter Widdess-Walsh, Simone Picelli, David C. Henshall, Vijay K. Tiwari

**Affiliations:** 1 Institute for Molecular Medicine, University of Southern Denmark, Odense, Denmark.; 2 FutureNeuro Research Ireland Centre for Translational Brain Science and; 3 Department of Physiology & Medical Physics, RCSI University of Medicine & Health Sciences, Dublin, Ireland.; 4 Department of Neurology and Clinical Neurophysiology, Beaumont Hospital, Dublin, Ireland.; 5 Strategic Academic Recruitment Doctor of Medicine Programme, RCSI University of Medicine and Health Sciences in collaboration with Blackrock Clinic, Dublin, Ireland.; 6 Institute of Molecular and Clinical Ophthalmology Basel (IOB), Basel, Switzerland.; 7 Department of Neurosurgery, Beaumont Hospital, Dublin, Ireland.; 8 Danish Institute for Advanced Study (DIAS), Odense, Denmark.; 9 Department of Clinical Genetics, Odense University Hospital, Odense, Denmark.; 10 Wellcome-Wolfson Institute for Experimental Medicine, School of Medicine, Dentistry, and Biomedical Science, Queens University Belfast, Belfast, United Kingdom.

**Keywords:** Neuroscience, Epilepsy

## Abstract

The availability and integration of electrophysiological and molecular data from the living brain is critical in understanding and diagnosing complex human disease. Intracranial stereo electroencephalography (SEEG) electrodes used for identifying the seizure focus in patients with epilepsy could enable the integration of such multimodal data. Here, we report multimodal profiling of epileptic brain activity via explanted depth electrodes (MoPEDE), a method that recovers extensive protein-coding transcripts, including cell type markers, DNA methylation, and short variant profiles from explanted SEEG electrodes matched with electrophysiological and radiological data allowing for high-resolution reconstructions of brain structure and function. We found gene expression gradients that corresponded with the neurophysiology-assigned epileptogenicity index but also outlier molecular fingerprints in some electrodes, potentially indicating seizure generation or propagation zones not detected during electroclinical assessments. Additionally, we identified DNA methylation profiles indicative of transcriptionally permissive or restrictive chromatin states and SEEG-adherent differentially expressed and methylated genes not previously associated with epilepsy. Together, these findings validate that RNA profiles and genome-wide epigenetic data from explanted SEEG electrodes offer high-resolution surrogate molecular landscapes of brain activity. The MoPEDE approach has the potential to enhance diagnostic decisions and deepen our understanding of epileptogenic network processes in the human brain.

## Abstract 2

We used SEEG electrodes from epilepsy patients to gather RNA, DNA, and protein data, revealing unique molecular profiles tied to brain activity, potentially improving epilepsy diagnosis and treatment.

## Graphical abstract

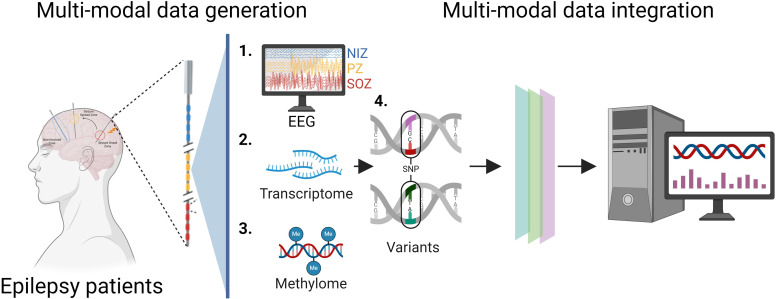

## Introduction

An improved understanding of human brain function requires the use of systems-level approaches that sample and integrate multimodal data spanning several orders of magnitude, from single molecules through individual cells, to local networks, and up to higher order organization (1, 2). Combining RNA-Seq and genome architecture maps with in vivo recording technologies and brain imaging enables the production of high-resolution reconstructions of the structure and function of the mammalian brain in health and disease. Indeed, increasingly comprehensive taxonomies of the mammalian brain have been achieved by integrating sequencing approaches with other modalities. These studies have demonstrated that neuronal phenotypes, including morphology, location, and electrophysiologic properties, are strongly defined by cell type–specific transcriptional and epigenetic signatures for different neuron subtypes (3–5) across different layers of the cortex (3, 4, 6) and for structures throughout the mouse brain including the hippocampus (5–7). Transcription factors are a principle driver of cellular diversity, regional specialization of function, and neurotransmitter type which in turn establishes the electrophysiological properties of different cell types (7). Equivalent mapping exercises for the human brain have emerged, with recent single-cell transcriptional and epigenetic studies revealing the distinct molecular programs that define neuronal and nonneuronal cell type and diversity, network and regional organization, and complexity (8–10).

Treatment-resistant epilepsies represent unique opportunities for access to the living human brain. Ex vivo electrophysiologic, pharmacologic, and gene expression profiling of surgically resected material has enabled important advances in brain function in health and disease (11–13). The analysis from implanted electrodes, placed to guide surgical decisions, has also enabled studies of human brain function (14), including elucidating network behavior during seizure onset (15) and how epileptiform activity can interfere with memory (16). Intracranial electroencephalography (EEG) recordings from stereotactically implanted electrodes (known as stereoelectroencephalography [SEEG]) are performed for a subset of patients with difficult-to-localize focal epilepsy to identify seizure-onset zones. The implantation of, typically, between 5 and 15 electrodes with multiple contact sites into deep brain structures yields necessary spatial and temporal mapping of hyperexcitable epileptogenic tissue, enabling localization of the seizure onset zone (SOZ), the associated propagation zones (PZs), and differentiation from the normal brain (14, 17). The neurophysiologic findings are then combined with imaging, computational tools, and clinical information to guide surgical decisions, with operations performed upon explantation of SEEG electrodes (17–19). Researchers recently reported that DNA present on explanted SEEG electrodes can be used to identify somatic mutations in patients with preresection epilepsy (20–22). Notably, a gradient of mosaic gene variation may be present in relation to recorded epileptiform activity (21). Mapping epigenetic marks using such material may also have utility for subtyping malformations of cortical development such as focal cortical dysplasia (FCD) (23). Assembling comprehensive molecular architectures that comprise read-outs of gene activity — for example, seizure-regulated gene transcripts or inflammatory signals — in combination with recorded neurophysiology would offer powerful insights into human brain function and causal mechanisms of epilepsy, potentially supporting surgical decision-making. There are, however, several unknowns. What additional nucleic acids can be reliably obtained from explanted electrodes, in particular mRNA transcripts? Furthermore, do these correspond to epigenetic marks that influence chromatin state? Do the signals retain information about cell types and implantation locations? Finally, epilepsies are heterogeneous in clinical presentation, semiology, and underlying mechanism, so can this approach be applied across surgical candidates with different etiologies, and is it scalable from highly focal epilepsies through to those affecting larger structures and networks? Indeed, defining the extent of epilepsy with existing technology is one of the greatest challenges in epileptology; hence, novel methodological developments are urgently required.

Here we report a method called multimodal profiling of epileptic brain activity via explanted depth electrodes (MoPEDE) in which an extensive repertoire of protein-coding transcripts including immediate early genes and mediators of inflammation, DNA methylation, and variant profiles can be recovered from explanted SEEG electrodes matched with recorded neurophysiological and radiological data. Our findings provide proof of concept that (a) RNA profiles and genome-wide epigenetic surveillance can be obtained from explanted SEEG electrodes and (b) these provide surrogate molecular landscapes of human brain activity at high resolution that may support diagnostic decisions as well as improve our understanding of the epileptogenic process in the live human brain.

## Results

### Multimodal profiling of SEEG electrode surfaces from patients with epilepsy.

The MoPEDE method utilizes intracranial SEEG electrodes to measure electrophysiological activity in various brain regions, including deeper structures such as the hippocampus, amygdala, and insula, providing a comprehensive sampling of these areas. Previous studies have demonstrated that these electrodes can be used to identify brain-specific somatic mutations in different epileptic brain regions (20–22).

**Figure 1:**
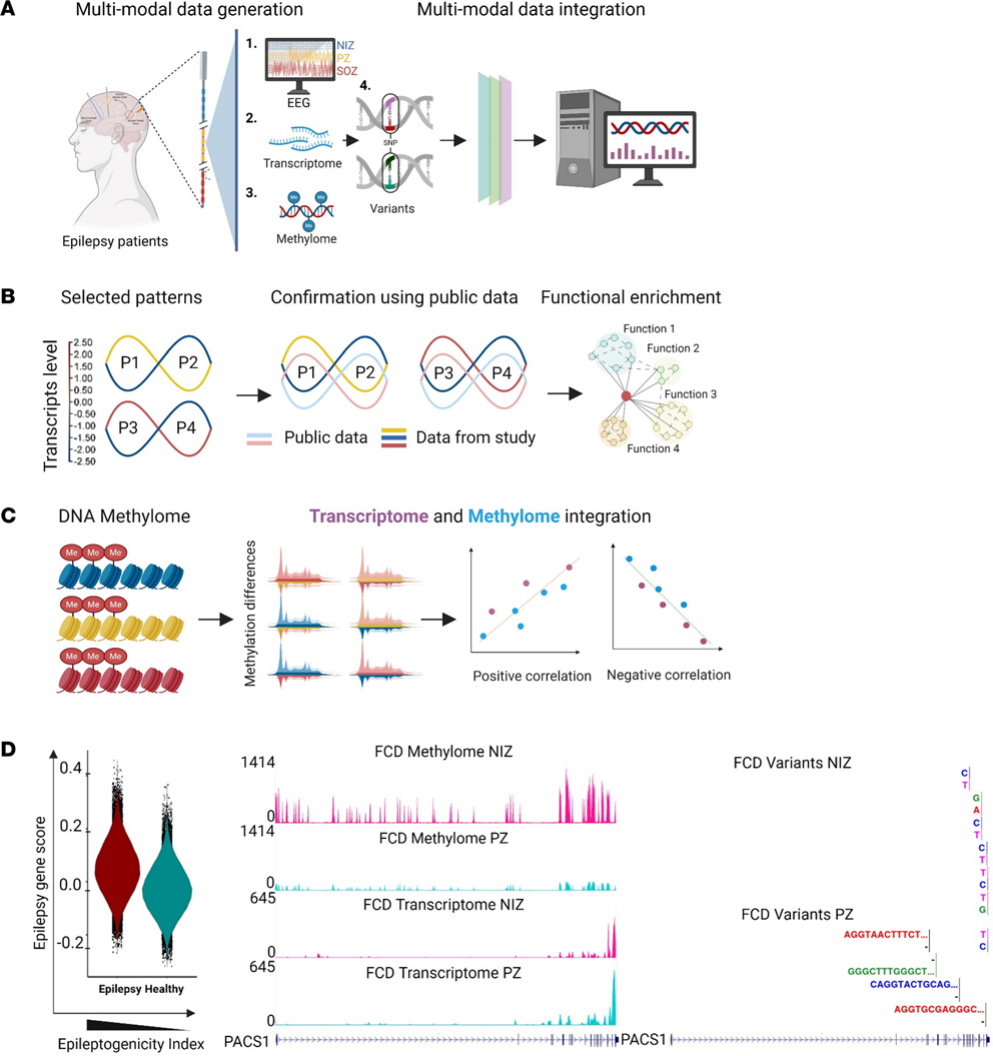
Schematic framework for multimodal profiling of different epilepsy subtypes using SEEG electrodes. (**A**) Overview of multimodal data integration of single-source EEG, whole transcriptome, methylome, and short variants profiles from electrodes collected from FCD, TLE, and RE brains. (**B**) We distinguished different sets of signatures based on differential gene expression and validated their patterns using multiple publicly available epilepsy data, followed by functional enrichment analysis. (**C**) Whole-methylome profiles were generated using the same samples and identified DMRs by investigating positive and negative correlations between the transcriptome and methylome data. (**D**) Snapshot of the integration of electrophysiology data with transcriptome signatures and a single-resolution map illustrating the correlation between transcriptome, methylome, and variants levels for a known epilepsy risk-associated gene (*PACS1*) in both PZ and NIZ electrodes from an FCD brain.

Here, we collected explanted SEEG electrodes from 3 patients with distinct epilepsy subtypes (Figure 1 and Table 1). Based on EEG profiles, we stratified the electrodes into: (a) SOZ, (b) PZ, and (c) noninvolved zone (NIZ). We then sectioned the electrodes from the SOZ, PZ, and NIZ for each patient and extracted total nucleic acid (RNA and genomic DNA [gDNA]) from the same sample. We then performed both transcriptome (RNA-Seq) and epigenome (DNA methylation) profiling on these samples. Additionally, we identified short variants (single nucleotide polymorphism [SNP], insertion-deletions [INDELs]) using RNA-Seq datasets (Figure 1A).

We first analyzed the transcriptome data, focusing on the quality and quantity of material that could be recovered and on specific expression patterns between the SOZ, PZ, and NIZ (e.g., up- or downregulated in SOZ/PZ versus NIZ comparisons) (Figure 1B). Furthermore, we then compared the detected transcripts and patterns to publicly available epilepsy data of transcriptome (bulk and single-cell/nucleus RNA-Seq) and epilepsy-related genes (24–27). Next, we conducted functional enrichment analysis to identify the biological processes (Figure 1B). For DNA methylation analysis, we examined the same samples (SOZ, PZ, and NIZ) to identify differentially methylated regions (DMRs). We integrated the transcriptome data with the methylome data to understand how DNA methylation profiles correlate with corresponding transcriptome profiles (Figure 1C). Finally, we explored whether we could integrate our molecular data with the SEEG neurophysiology (visual analysis and epileptogenicity index score) (Figure 1D).

### Patient neurophysiology, neuroimaging, and neuropathological features.

**Figure 2:**
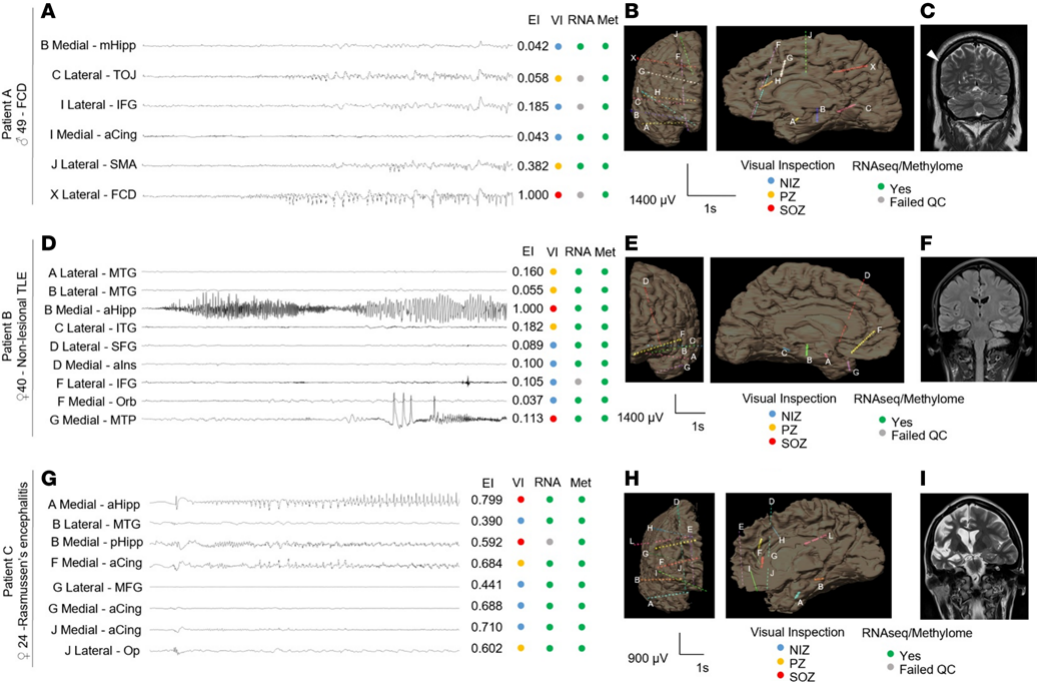
Neurophysiology, neuroimaging, and neuropathological features of study participants. (**A**, **D**, **G**) Representative sample SEEG ictal recording with matched Epileptogenicity Index, epileptogenic network involvement based on visual inspection (VI), and presence or absence of contact points in the molecular analysis. (**B**, **E**, **H**) Letters correspond to depth electrodes indicated in **B**, **E**, and **H**; bipolar recording. At ictal onset, there is fast repetitive spiking and paroxysmal fast activity. High-pass filter 5 Hz, low-pass filter 80 Hz, Notch 50 Hz. Coronal and mesial sagittal volumetric MRI reconstruction (**B**, **E**, **H**) showing the location of the implanted SEEG electrodes for patients A, B, and C. (**C**) Coronal T2-weighted MRI sequence of patient A. Arrow indicates the location of the FCD. (**F**) Coronal MRI FLAIR sequence of patient B demonstrating normal temporal lobe structures. (**I**) Coronal T2-weighted MRI sequence of patient C showing diffuse right hemisphere atrophy including the hippocampus, consistent with RE. Met, methylome sequencing; NIZ, noninvolved zone; PZ, propagation zone; SOZ, seizure onset zone; MTG, middle temporal gyrus; mHipp, midhippocampus; TOJ, temporo-occipital junction; pHipp, posterior hippocampus; aHipp, anterior hippocampus; SFG, superior frontal gyrus; MFG, middle frontal gyrus; IFG, inferior frontal gyrus; Orb, orbitofrontal; aCing, anterior cingulate; SMA, supplementary motor area; ITG, inferior temporal gyrus; aIns, anterior insula; MTP, mesial temporal pole; Op, opercular.

To assess the broad applicability of our method, we recruited 3 patients with epileptic foci of diverse etiology and extent: FCD, nonlesional temporal lobe epilepsy (TLE), and Rasmussen’s encephalitis (RE) (Table 1). Each patient was discussed at an interdisciplinary patient management conference, where intracranial evaluation with SEEG was recommended exclusively based on clinical indication. All participants underwent robot-assisted SEEG monitoring. Depth electrodes with 12, 15, or 18 contacts were implanted based on a preoperative hypothesis of the SOZ. By standard nomenclature, each depth is labeled with a letter, with electrode contacts numbered from mesial to lateral. The anatomic location of each electrode was confirmed by postoperative computed tomography (CT) coregistered with preoperative volumetric MRI (Figure 2). Continuous EEG recordings of seizures were conducted with concurrent video, and visual analysis classified cortical regions into the SOZ, PZ, or NIZ. For quantitative SEEG analysis, representative seizures with minimal artifacts occurring over 48 hours after implantation were selected. The epileptogenicity index (EI) at each electrode pair was measured to assess changes in energy ratio and time delay from electrode contacts from seizure onset, estimating the epileptogenicity of cortical regions (28, 29).

Patient details and operational outcomes are in Table 1. The first patient had medication-resistant focal epilepsy due to a right parietal FCD type IIA (Figure 2, A–C, Supplemental Figure 1A, and Supplemental Table 1; supplemental material available online with this article; https://doi.org/10.1172/jci.insight.184518DS1). During SEEG monitoring, 33 electroclinical seizures were recorded, with EEG onset from the lateral contacts of the depth electrodes at the parietal FCD (Table 1). Seizure propagation involved the supplementary motor area and temporo-occipital junction. We collected 18 electrode samples from NIZ, PZ, and SOZ regions and then selected ~40% of electrodes covering the NIZ, PZ, and SOZ regions for whole-transcriptome and DNA methylome profiling (Figure 2A).

The second patient had MRI-negative, medication-resistant left TLE (Figure 2, D–F; Table 1; Supplemental Figure 1, B–D; and Supplemental Table 1). SEEG monitoring recorded 6 electroclinical seizures, with EEG onset from the anterior hippocampus and the temporal pole. Seizure propagation involved the middle temporal gyrus and inferior temporal gyrus. We collected 12 electrode samples from the NIZ, PZ, and SOZ regions. We then profiled approximately 75% of these electrodes, covering all 3 regions (NIZ, PZ, and SOZ), for whole transcriptome and DNA methylation (Figure 2D).

The third patient was diagnosed with RE at age 15 years and was treated medically (Figure 2, G–I; Supplemental Figure 1, E–G; and Supplemental Table 1). Persistent medication-resistant epilepsy led to SEEG monitoring, during which 32 electroclinical seizures were recorded, primarily from the hippocampus and the gyrus rectus. Seizure propagation involved the cingulate and frontal operculum. We collected 20 electrode samples from the NIZ, PZ, and SOZ regions. We then profiled all 3 regions (NIZ, PZ, and SOZ), encompassing approximately 40% of the electrodes, for whole transcriptome and DNA methylation (Figure 2G).

We first investigated whether the collected explanted SEEG contacts retained any intact cells. For this, electrodes were cut into small pieces and rinsed with PBS for collecting cells. Trypan blue staining confirmed the presence of cells on these electrodes (Supplemental Figure 2A). We then extracted total nucleic acids (both RNA and gDNA) from the eluate of rinsed electrode contacts and measured their quantity using a Nanodrop spectrophotometer. Remarkably, we obtained significant concentrations of nucleic acids from these electrodes, with amounts directly proportional to the number of metal contact points in the sample (Supplemental Figure 2B). Next, we analyzed the quality of these nucleic acids using a high-sensitivity fragment analyzer, finding both gDNA and RNA in considerable concentrations (Supplemental Figure 2, C–E, and Supplemental Tables 2 and 3). We then divided these samples into equal portions and purified the gDNA and RNA separately, and the libraries were prepared for sequencing (Supplemental Figure 2, F and G, and Supplemental Tables 4–6). The purified RNA from most contacts exhibited acceptable RNA integrity numbers (RINs) (Supplemental Figure 2, A and G). These results indicate that SEEG electrodes used in the presurgical evaluation of epilepsy carry sufficient nucleic acid material from the implanted brain regions to enable the transcriptome and epigenome of living people with epilepsy.

### Single-sourced multi-ome profiles of SEEG electrode contacts show high sequence coverage and mapping.

**Figure 3:**
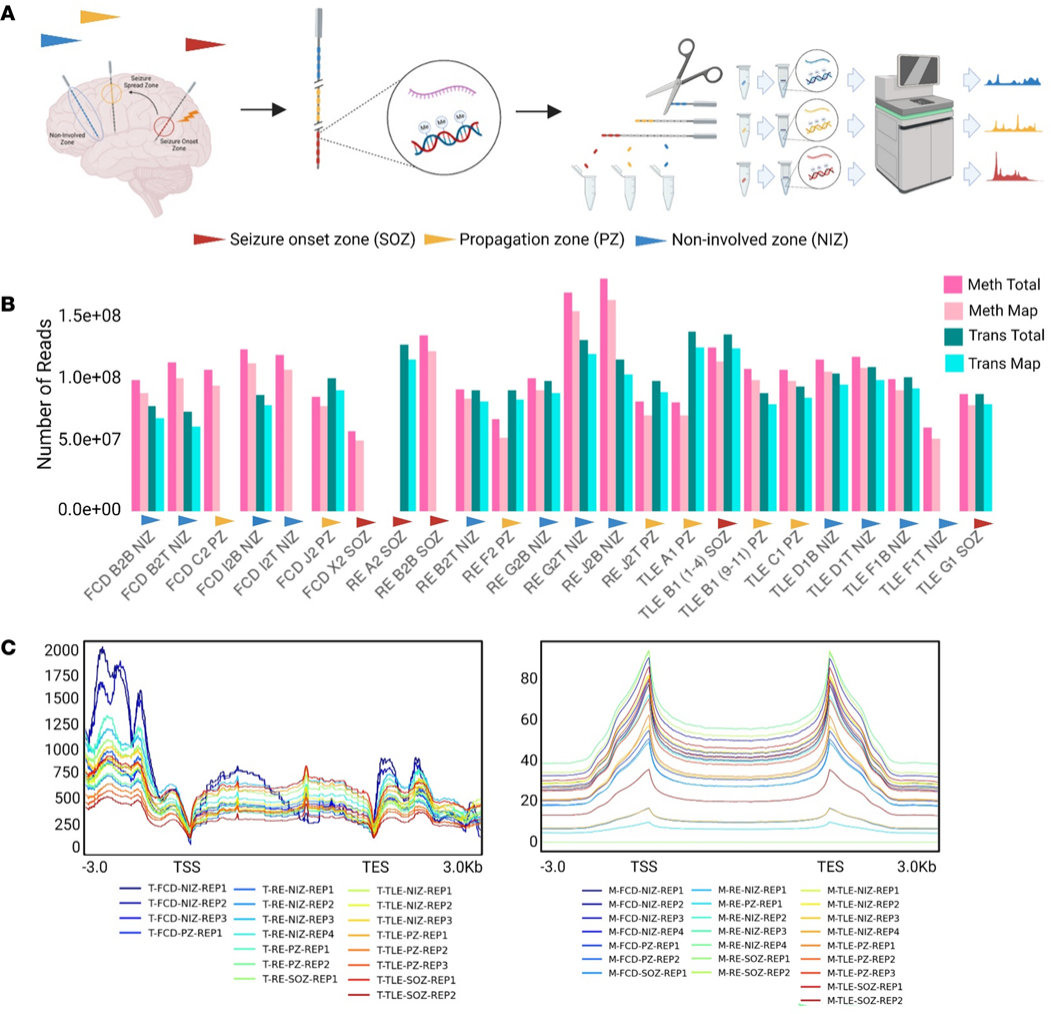
Single-source high throughput multi-omic profiling of EEG electrodes in epilepsy. (**A**) Selection of EEG electrodes: EEG electrodes were selected from SOZ, PZ, and NIZ. The attached nucleic acid material was extracted from each electrode, and whole transcriptome and methylome data were generated. (**B**) Seven electrodes explanted from the brain of a patient with FCD were used. On average, 100 million and 85 million reads were sequenced for the methylome and transcriptome, respectively. Notably, 91 million and 74 million methylome and transcriptome reads, respectively, mapped to the human genome. Similarly, 9 electrodes explanted from the patient with TLE were used. An average of 100 million and 95 million reads were sequenced for the methylome and transcriptome, respectively. Prominently, an average of 95 million and 87 million methylome and transcriptome reads, respectively, mapped to the human genome. Additionally, 8 electrodes explanted from the patient with RE were used. On average, 117 million and 101 million reads were sequenced for the methylome and transcriptome, respectively. Importantly, an average of 105 million and 92 million methylome and transcriptome reads, respectively, mapped to the human genome. (**C**) Genome-wide transcriptome and methylome density. The transcriptome and methylome density of each electrode from all 3 patients were plotted across gene body and flanking regions (3 kb). A consistent negative correlation between methylation and transcription was observed across all electrodes.

To achieve comprehensive sequence coverage in the transcriptome of very low–input RNA samples from the SEEG electrodes, we applied the Flash-Seq method (30). Originally developed for full-length single-cell RNA-Seq (scRNA-Seq), we adapted Flash-Seq for bulk RNA-Seq in our study (31). This allowed us to obtain full-length transcripts with high coverage from electrodes, performing both transcriptome and DNA methylome analysis (Figure 3A). Although some of the samples had low RINs, it did not affect the coverage of varied ranges of transcripts, indicating sufficient RNA quality (Supplemental Figure 3, A and B, and Supplemental Figure 4, A and B). Furthermore, we observed a correlation between the electrode-derived transcriptome and the transcriptome of the subsequently removed tissue in which that electrode had previously been implanted (Supplemental Figure 5, A–D). This strongly indicates that explanted SEEG electrodes retain a representative transcriptional profile from the implantation site.

From the FCD case, we used 7 different electrode samples, achieving an average of 100 million and 85 million reads per sample in DNA methylome and transcriptome sequencing, respectively. Of these, 91 million reads from the methylome and 76 million reads from the transcriptome were mapped to the human genome (Figure 2D and Figure 3B). From the TLE case, we used 9 electrode samples, resulting in an average of 100 million and 106 million reads per sample in DNA methylome and transcriptome sequencing, respectively. Some 90 million methylome reads and 97 million transcriptome reads were mapped for the TLE case to the human genome (Figure 3B). One of the RNA samples failed in quality control (QC) and was excluded from sequencing (Figure 2H and Figure 3B). From the RE case, 8 electrode samples were analyzed, yielding an average of 117 million and 101 million reads per sample in DNA methylome and transcriptome sequencing, respectively. Of these, 105 million methylome reads and 97 million transcriptome reads were mapped to the human genome (Figure 3B). One of the RNA samples failed in QC and was excluded from sequencing (Figure 3B).

We then performed genome-wide density analysis for mapped transcriptome reads across the transcriptional start site (TSS) and transcriptional end site (TES) , covering a 3 kb upstream and downstream flanking window (Figure 3C). Similarly, we analyzed the density of mapped DNA methylation reads across the TSS and TES with a 3 kb flanking window (Figure 3C). As expected, these density plots revealed a consistent negative correlation between the transcriptome and DNA methylome across all samples analyzed (Figure 3C). Thus, it is possible to obtain high-coverage multi-ome profiles from SEEG electrode contacts from presurgical patients with epilepsy.

### Transcriptome analysis of SEEG electrodes reveals signatures of epileptic brain regions.

**Figure 4:**
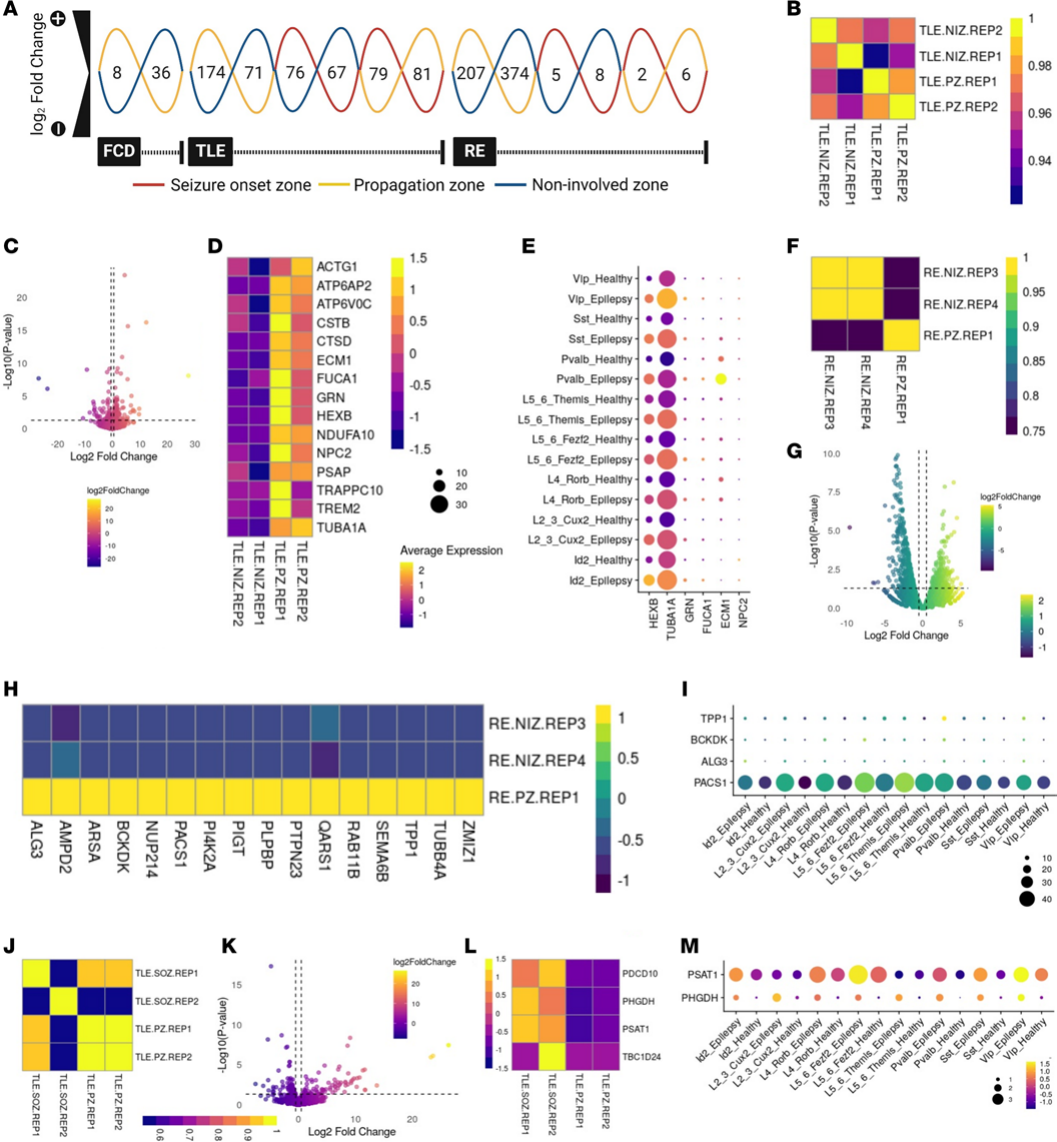
Distinct transcriptional signatures are activated in different epileptic brain regions. (**A**) Differentially expressed genes (DEGs; log_2_ fold change ± 0.5 and *P* ≤ 0.05) identified in epileptic brains. (**B** and **C**) Plot showing the correlation between different replicates, and the distribution of DEGs in PZ versus NIZ brain regions for patients with TLE. (**D**) Heatmap of upregulated genes enriched in epilepsy-related genes (*P* ≤ 0.05). (**E**) Dot plot showing upregulated genes enriched in upregulated (TLE versus Control scRNA, *P* ≤ 0.05) interneurons and neurons in patients with epilepsy compared with healthy controls and epilepsy-related genes (*P* ≤ 0.05). (**F** and **G**) Correlation between replicates and volcano plot showing DEGs from the NIZ and PZ brain regions of patients with RE. (**H**) Heatmap of upregulated genes enriched in epilepsy-related genes (*P* ≤ 0.05). (**I**) Dot plot showing upregulated genes enriched in upregulated (TLE versus Control scRNA, *P* ≤ 0.05) genes of interneurons and neurons in patients with epilepsy compared with healthy controls, as well as in epilepsy-related genes (*P* ≤ 0.05). (**J**) Plot showing the correlation between different replicates. (**K**) Distribution of DEGs in SOZ versus PZ brain regions for the patient with TLE. (**L**) Heatmap of upregulated genes enriched in epilepsy-related genes (*P* ≤ 0.05). (**M**) Dot plot showing upregulated genes enriched in TLE versus Control scRNA (*P* ≤ 0.05) of interneurons and neurons in patients with epilepsy compared with healthy controls and epilepsy-related genes (*P* ≤ 0.05). Color code: plasma, **B**–**E** (TLE PZ versus NIZ) and **J**–**M** (TLE SOZ versus PZ); viridis, **F**–**I** (RE PZ versus NIZ).

To delineate transcriptional changes associated with epilepsy, we performed a differential expression analysis comparing SOZ, PZ, and NIZ (Figure 1B and Figure 4A). To validate the transcriptome profile from SEEG electrodes, we analyzed previously published single nuclei RNA-Seq (snRNAseq) data (26, 27) from surgically obtained epilepsy samples and matched controls (Supplemental Figure 6, A–C). We integrated epilepsy and healthy single nuclei profiles and annotated different clusters using markers for various excitatory and inhibitory neurons (Supplemental Figure 6, A and B). Furthermore, we found our DEGs enriched in previous studies of TLE and RE bulk and scRNA-Seq transcriptome including key epilepsy-related genes (Supplemental Figure 6, D and E) (24–27).

For the FCD patient sample, only the PZ and NIZ samples were sequenced. We performed transcriptome correlations comparing PZ versus NIZ and found they were poorly correlated (Supplemental Figure 7A). We identified 8 significantly upregulated and 36 downregulated genes in the PZ compared with the NIZ (Figure 4A, Supplemental Figure 7B, and Supplemental Tables 7 and 8). GO enrichment analysis indicated that genes activated in PZ areas were involved in translational processes (Supplemental Figure 7C and Supplemental Table 9), whereas the downregulated genes were mostly involved in metabolic processes (Supplemental Figure 7D). We next compared the differentially expressed genes with known epilepsy-associated genes (26) and found 2 genes in our list (Supplemental Figure 7E and Supplemental Table 10). In contrast, some genes (*RPL4*, *MTPAP*, and *SNX30*) that are downregulated in the PZ also showed downregulation in epilepsy samples in the TLE versus healthy scRNA dataset (Supplemental Figure 7F) (27).

For the patient with TLE, we observed that the replicates from PZ and NIZ regions were anticorrelated, whereas a high correlation was observed within the replicates from the same regions (Figure 4B). Next, we identified 174 significantly upregulated and 71 downregulated genes in the PZ compared with NIZ (Figure 4, A and C, and Supplemental Tables 11 and 12). The upregulated genes were enriched for processes related to translation, gene expression, and peptide and macromolecule biosynthetic pathways (Supplemental Figure 8A and Supplemental Table 13), while the downregulated genes were enriched for functions related to superoxide generation, cytokine production, and various stress signaling pathways (Supplemental Figure 8B and Supplemental Table 14). We found a set of transcripts upregulated in PZ versus NIZ that were previously reported as enriched among upregulated genes in bulk and single nucleus RNA-Seq analyses of resected TLE tissue and contain known epilepsy-related genes (24–27) (Figure 4, D and E, and Supplemental Figure 6D).

Next, we compared the SOZ versus NIZ in TLE and found 76 genes significantly upregulated and 67 genes downregulated, respectively (Figure 4A, Supplemental Figure 8D, and Supplemental Tables 15 and 16). A correlation analysis for SOZ versus NIZ found that they are highly correlated within the replicates from same regions (Supplemental Figure 8C). We observed that genes upregulated in the same comparison were enriched and upregulated in the previously published single nuclei profiles from the neocortex of patients with TLE (27) (Supplemental Figure 6D and Supplemental Figure 8E). Similarly, the upregulated genes were also found to be enriched in RNA-Seq data from NeuN^+^ cells from the neocortex of patients with TLE (25). Furthermore, upregulated genes were highly enriched for cellular responses to zinc, copper, and cadmium ions (Supplemental Figure 8F and Supplemental Table 17). In contrast, the downregulated genes were enriched for immune response, cell junction disassembly, and synapse pruning (Supplemental Figure 8G and Supplemental Table 18).

We further compared the transcriptome of SOZ versus PZ for TLE, where 79 genes were significantly upregulated and 81 genes were downregulated, respectively (Figure 4, A, J and K, and Supplemental Tables 19 and 20). The upregulated genes were enriched for cellular responses to ions, dephosphorylation, cell fate commitment, and stem cell differentiation (Supplemental Figure 9A and Supplemental Table 21). In contrast, the downregulated genes were enriched for proteolysis, protein catabolic process, and peptidase activity (Supplemental Figure 9B and Supplemental Table 22). We also observed that epilepsy-associated genes *PDCC10*, *PHGDH*, *PSAT1*, and *TBC1D24* showed higher expression levels in SOZ compared with PZ (Figure 4, L and M) (26). Notably, 2 of them were upregulated and enriched in TLE versus healthy scRNA data (27) (Supplemental Figure 6D and Figure 4M).

From the RE patient SEEG samples, we detected 207 significantly upregulated and 374 downregulated genes in the PZ compared with the NIZ (Figure 4, A and G, and Supplemental Tables 23 and 24). When performing the correlation analysis, we found PZ versus NIZ replicates were less correlated (Figure 4F). Epilepsy-associated genes (*TPP1*, *BCKDK*, *ALG3*, and *PACS1*) were upregulated and enriched in PZ and showed higher expression in the TLE as compared with the healthy control scRNA data (Figure 4, H and I, and Supplemental Figure 6D) (26, 27). In addition, upregulated genes were enriched in TLE NEUN^+^, OLIG2^+^, and RE as compared with the healthy controls (24, 25). The upregulated genes were associated with processes such as nuclear export, amino acid transport, cell migration, and positive regulation of gene expression (Supplemental Figure 10A and Supplemental Table 25), whereas the downregulated genes were enriched for translation, peptide and macromolecule biosynthesis, and gene expression (Supplemental Figure 10B and Supplemental Table 26).

When we compared SOZ versus PZ in the patient with RE, we found that 2 and 6 genes were significantly up- and downregulated, respectively (Figure 4A; Supplemental Figure 9, C and D; and Supplemental Tables 27 and 28). The upregulated genes were associated with the negative regulation of the cell cycle, apoptotic process, regulation of necrotic cell death, and cellular respiration (Supplemental Figure 9E and Supplemental Table 29), whereas the downregulated genes were associated with the positive regulation of amyloid-β formation, catabolic process, and ubiquitin-dependent proteolysis (Supplemental Figure 9F and Supplemental Table 30).

In the SOZ compared with the NIZ, 5 genes were upregulated, and 8 genes were downregulated significantly (Figure 4A, Supplemental Figure 10D, and Supplemental Tables 31 and 32), and they showed less correlation with each other (Supplemental Figure 10C). Among the upregulated genes in the SOZ, the *QPCT* gene showed higher expression in epilepsy compared with healthy controls (Supplemental Figure 10F) (27). GO enrichment analysis revealed that genes activated in the SOZ were involved in the negative regulation of cell cycle and cellular response to stress (Supplemental Figure 10E and Supplemental Table 33). In contrast, downregulated genes were associated with amyloid β response and cholesterol import (Supplemental Figure 10G and Supplemental Table 34).

Overall, the SEEG transcriptional signatures from SOZ and PZ show a range of differential gene expression and shared as well as distinct biological processes for the 3 etiologies. Furthermore, our SEEG-recovered gene signatures highly correlated with tissue-based epilepsy bulk and scRNA-Seq transcriptome data showing the potential of our approach in capturing the molecular and cellular mechanisms underlying different epilepsy subtypes (24–27) (Supplemental Figure 6, D and E).

### Integration of SEEG transcriptome and DNA methylome data.

Aberrant DNA methylation has previously been reported in resected tissues from patients with epilepsy (32–35). Here, we investigated DNA methylation maps derived from the explanted SEEG electrodes from the patients with epilepsy. We began by comparing the DMRs between the SOZ, PZ, and NIZ. For the FCD case, we observed an overall raised level of transcriptome and methylome in NIZ compared with PZ in the gene body and flanking regions, and these were poorly correlated (Supplemental Figure 11, C–E). We found that 216 regions were hypermethylated and 1,801 regions were hypomethylated between PZ and NIZ (Supplemental Figure 11F). GO term analysis revealed that the hypomethylated regions were enriched for genes involved in morphogenesis and GTPase-mediated signal transduction (Supplemental Figure 11G), whereas hypermethylated genes were enriched for cholesterol storage and nuclear membrane disassembly (Supplemental Figure 11H). Notably, hypomethylated genes were enriched in epilepsy-related genes (26) (Supplemental Figure 11B).

**Figure 5:**
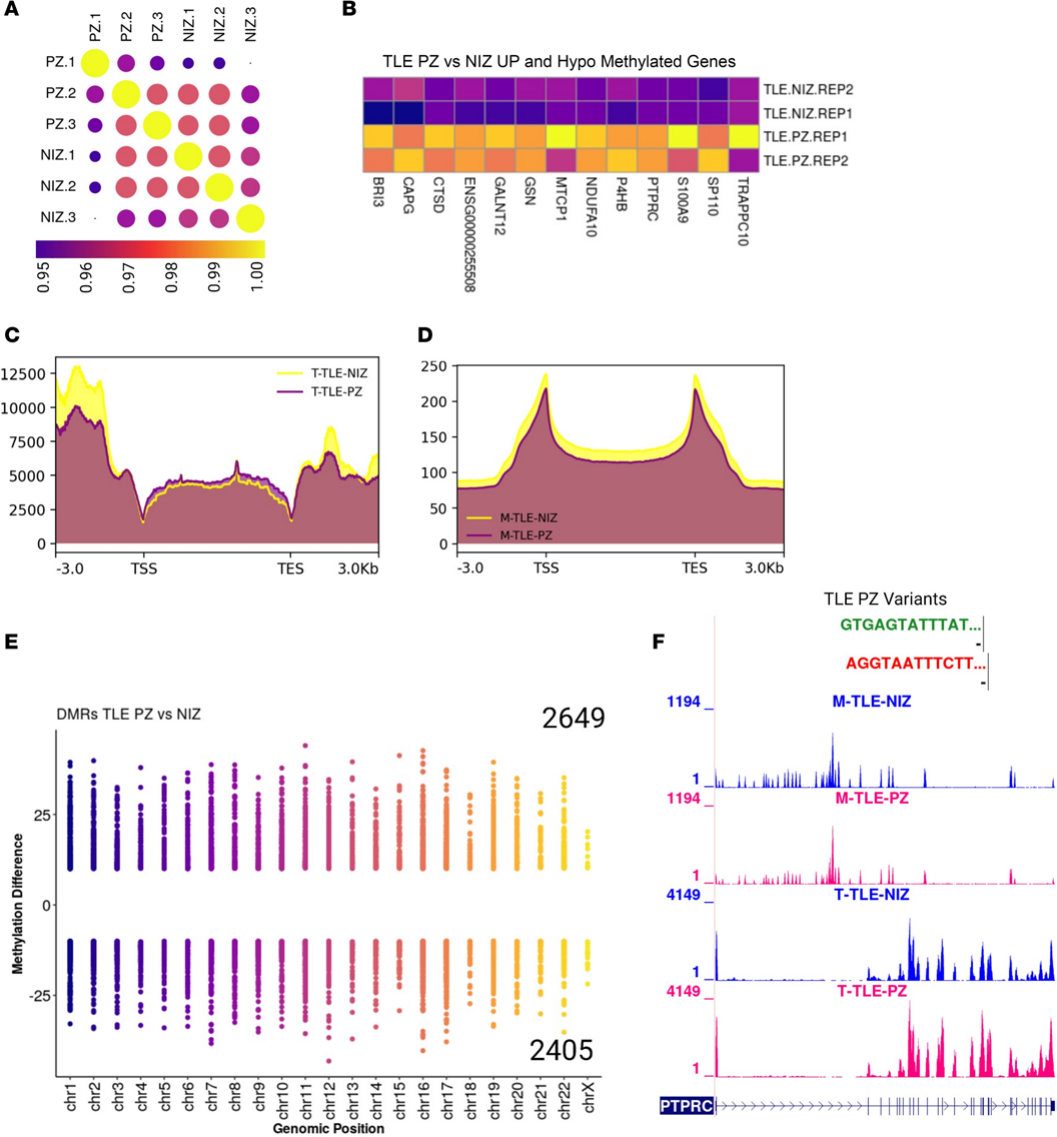
Insights into the epigenetic dysregulation in epilepsy. (**A**) Replicate-wise correlation of methylation of CpG context from PZ and NIZ regions of TLE brain. (**B** and **E**) DMRs in PZ versus NIZ comparison with genes negatively correlated with their transcriptome and methylome level. (**C** and **D**) Transcriptome and methylome levels are elevated in NIZ electrodes compared with PZ electrodes in TLE brain. Notably, transcriptome and methylome show an antagonistic correlation across the gene body. Decreased methylome levels in these regions accompany increased transcriptome levels in the gene body and flanking regions. Conversely, decreased transcriptome levels at the TSS and TES coincide with increased methylome levels at these sites. (**E**) A higher number of hypermethylated regions (2649) compared with hypomethylated regions (2405) was identified in PZ versus NIZ electrode comparisons. (**F**) Integration of transcriptome and methylome data reveal an inverse relationship between methylation and gene expression. A single-base resolution map was generated for *PTPRC*, an epilepsy-related gene, showing that depletion of methylation levels increased transcriptome levels in PZ electrodes. Conversely, increased methylation levels resulted correlated with in decreased transcriptome levels in NIZ electrodes for *PTPRC*. In addition, we also observed that *PTPRC* harbored short variants (SNPs/INDELs) in PZ regions in TLE brain.

For the TLE case, we found an increase in methylation and transcriptome levels in NIZ compared with PZ and SOZ, and they were less correlated with each other than was the PZ versus NIZ comparison (Figure 5, A, C, and D, and Supplemental Figure 13, A and B). DMR analysis of these regions showed 2,649 and 2,405 hyper- and hypomethylated regions, respectively (Figure 5E). We next investigated the link between gene expression and DNA methylation in PZ and NIZ regions in the TLE case by integrating single-source transcriptome and methylome data. This analysis revealed that methylation levels in PZ versus NIZ were negatively correlated with their transcriptome as expected (Figure 5B and Supplemental Figure 11A). In addition, hyper- and hypomethylated genes were enriched in genes related to epilepsy and those previously shown to have aberrant DNA methylation in drug-resistant patients with TLE (Supplemental Figure 11, A and B) (26, 36). Notably, transcriptome and methylome showed an inverse correlation across the gene body and flanking regions, with decreased DNA methylation levels accompanied by higher gene expression levels (Figure 5, C and D). Conversely, decreased transcription at the TSS and TES coincide with increased DNA methylation levels at these sites. The hypomethylated regions were enriched for GO terms, including phosphorylation and negative regulation of DNA binding of transcription factors (Supplemental Figure 12B), whereas the hypermethylated regions were enriched for GTPase-mediated signal transduction, regulation of cell migration, and phagocytosis (Supplemental Figure 12C). Using the example of epilepsy-associated gene *PTPRC*, we found a reduction in the DNA methylation (hypomethylation) level and an increase in transcription in the PZ compared with NIZ (Figure 5F).

Comparison of the DNA methylome of SOZ and NIZ in the TLE case showed a poor correlation (Supplemental Figure 12D). DMR analysis for these regions showed that 636 regions were hypermethylated and 475 regions were hypomethylated when comparing the SOZ with the NIZ area (Supplemental Figure 12E). Notably, these hyper- and hypomethylated genes have previously been found to be enriched in TLE patient brain tissue (36, 37). GO-term analysis of DMRs are provided in Supplemental Figure 12, F and G.

We found a positive correlation in DNA methylation levels among SOZ and PZ replicates (Supplemental Figure 13C). DMR analysis for these regions showed that 608 regions were hypermethylated and 872 regions were hypomethylated when comparing the SOZ to the PZ region (Supplemental Figure 13D). We observed that both hypermethylated and hypomethylated genes were significantly enriched in the data from the tissues of patients with drug-resistant TLE (Supplemental Figure 11, A and B) (36, 37). GTPase-mediated signal transduction, G-protein coupled receptor signaling, and negative regulation of cell proliferation–related terms were enriched for the hypomethylated regions (Supplemental Figure 13E). The hypermethylated regions were enriched for processes including phospholipid biosynthesis and CNS development (Supplemental Figure 13F).

Tissue DNA methylation landscapes have recently been reported for RE (38). For the SEEG samples from the patient with RE, we observed elevated transcriptome and DNA methylome levels in NIZ as compared with PZ and SOZ regions in gene body and flanking regions (Supplemental Figure 14, A and B). In addition, overall transcriptome and methylome levels were inversely proportional to each other in the gene body and flanking regions. Next, DNA methylation correlation analysis showed a high correlation among PZ and NIZ replicates (Supplemental Figure 14C). DMR analysis for these regions showed that 31 regions were hypermethylated and 328 regions were hypomethylated when comparing the SOZ and the PZ region (Supplemental Figure 14D). Furthermore, we observed transcriptionally upregulated genes negatively correlating with their DNA methylation levels (hypomethylated) in PZ as compared with NIZ (Supplemental Figure 14E). GO-term analysis of hypomethylated regions was enriched for sulfate biosynthesis, mesenchymal cell migration, and semaphorin-plexin signaling pathways (Supplemental Figure 14F).

### Combining neurophysiology with multi-omic data from SEEG electrodes.

**Figure 6:**
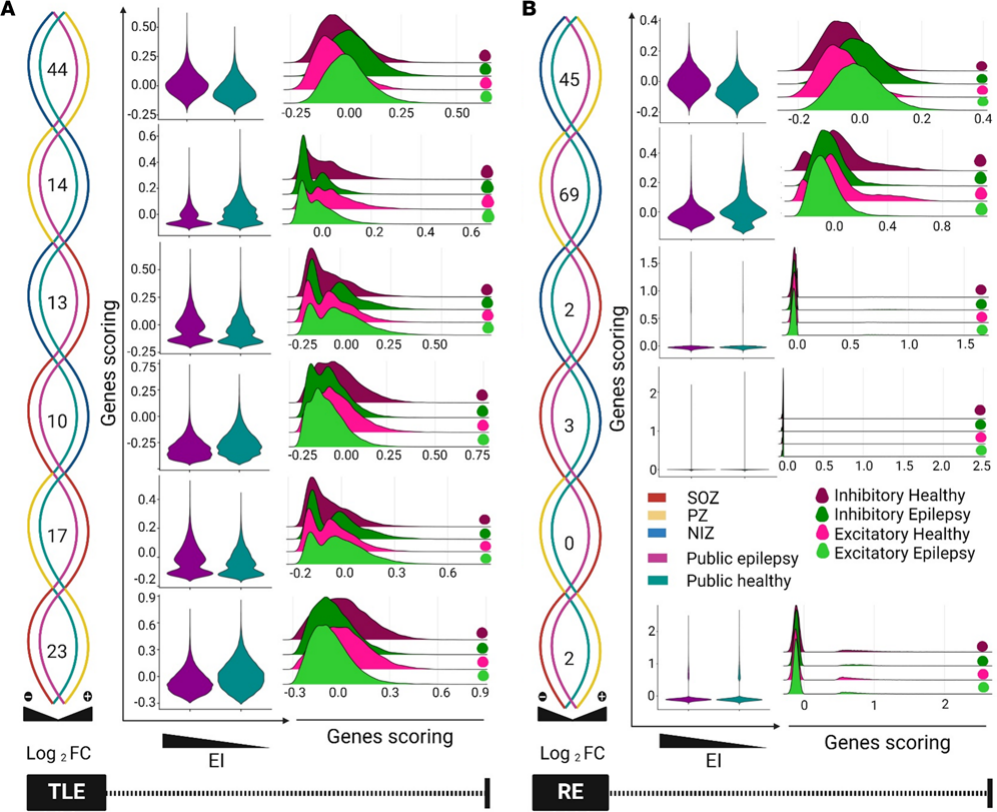
Transcriptional signatures associated with epileptogenicity index in epilepsy subtypes. (**A** and **B**) Relationship between the EI and transcriptional signatures in TLE and RE brains. This figure displays the relationship between the EI and transcriptional signatures across different brain regions (set of genes differentially expressed in the same direction in our data and public data). We analyzed SOZ, PZ, and NIZ regions of TLE and RE. The results show that transcriptional signatures associated with a high epileptogenicity index (SOZ) in TLE and RE were also highly activated in epilepsy samples compared with healthy controls (data from an independent public dataset). Similarly, transcriptional signatures associated with a moderate epileptogenicity index (PZ) in nonlesional TLE and RE displayed increased activation in epilepsy compared with healthy controls (data from an independent public dataset). The activity of transcriptional signatures in excitatory and inhibitory neurons present in epilepsy and healthy controls. The ridge plots show the activity of transcriptional signatures within excitatory and inhibitory neurons. We compared the scores of signature genes associated with a high epileptogenicity index in excitatory and inhibitory neurons from both epilepsy and healthy samples. The results reveal a higher correlation between activity scores and the epileptogenicity index for these signature genes in excitatory and inhibitory neurons of epilepsy samples compared with their counterparts in healthy controls.

Next, we explored the relationship between EI scores based on the recorded neurophysiologic signals and transcriptomic signatures. To accomplish this, we integrated the EI scores of the TLE and RE cases with transcriptome-derived signatures from NIZ, PZ, and SOZ and compared them with independent public epilepsy datasets (27). This analysis revealed that SOZ transcriptome signatures with high EI scores displayed elevated expression in epilepsy patient samples compared with healthy controls (27) (Figure 6, A and B). Similarly, transcriptional signatures linked with moderate EI scores in the PZ of the patients with TLE and RE showed increased activation in epilepsy samples relative to healthy controls (Figure 6, A and B).

Last, we explored the activity of these transcriptional signatures in excitatory and inhibitory neurons from both epilepsy and control samples. Our findings indicate that gene signatures from TLE and RE were positively correlated with EI scores in both excitatory and inhibitory neurons of patients with epilepsy compared with control counterparts (Figure 6, A and B). Overall, our analyses underscore that the expression profiles of gene signatures from SOZ and PZ of TLE and patients with RE closely mirrored those observed in existing epilepsy datasets. This shows the relevance of our approach to identifying epilepsy-related transcriptomic signatures from SEEG electrodes.

### SEEG-derived transcriptome can predict distinct genomic variants.

We investigated whether genomic variants can be detected in our SEEG-derived RNA transcript sequences. We explored the presence of short variants (SNPs and INDELs) in our transcriptome data using GATK (39) best practice workflows and annotated the high-quality variants through wANNOVAR (Supplemental Figure 15A). We then mapped the chromosome-wide distribution of variants in RE, where we compared the difference between NIZ and PZ (Supplemental Figure 15B). Similarly, we performed a chromosome-wide distribution of variants in TLE, where we compared the difference between NIZ, PZ, and SOZ (Supplemental Figure 15C).

Using the transcriptomic data from the TLE samples, we identified a total of 4,396 genes harboring the variants in NIZ, and among these, 2.8% were unique to the NIZ region. In the SOZ region, we found a total of 4,698 genes, among which were 9.5% specific to the SOZ region (Supplemental Figure 16, A and B). In the PZ region, we identified a total of 4,226 genes, and among these, 1.6% were unique to the PZ. In total, 3,588 genes (59.7%) were shared across NIZ, PZ, and SOZ (Supplemental Figure 16, A and B). We then overlaid these variants with known epilepsy-associated genes (26), and this analysis revealed that 3.9%, 3.9%, and 4.6% of genes were shared among NIZ, PZ, and SOZ, respectively (Supplemental Figure 16A). Next, we performed GO-term analysis for the 3,799 genes that were shared across the NIZ, PZ, and SOZ. Interestingly, this analysis showed that these variants were enriched for RNA-induced silencing complex (RISC) complex assembly, mRNA, pre-RNA processing, and RNA secondary structure unwinding pathways (Supplemental Figure 16C). NIZ-specific genes were enriched for negative regulation of inflammatory responses to antigens, negative regulation of DNA biosynthesis, and regulation of intracellular signaling (Supplemental Figure 16D). For the PZ region, genes were enriched for the regulation of cellular localization, apoptotic signaling pathways, and protein deubiquitylation-related GO-terms (Supplemental Figure 16E). The NIZ genes were enriched for intracellular pH elevation, nuclear pore organization and assembly, and glial cell differentiation GO-terms (Supplemental Figure 16F). We then analyzed the total number of genetic variants (INDELs and SNPs) present in SOZ, PZ, and NIZ and found that SOZ harbored more genetic variants compared with PZ and NIZ (Supplemental Figure 15D).

We also overlapped genes harboring the variants with known epilepsy-associated genes and found that 3.6% of genes were commonly present in PZ and NIZ (Supplemental Figure 17A). Similarly, in the RE brain, we found a total of 4,095 genes harboring the variants (517 unique) in the PZ area and 4,036 gene variants (458 unique) in the NIZ area; among these variants, 3,578 are present in both NIZ and PZ (Supplemental Figure 17B). We further annotated these genes using GO enrichment analysis (Supplemental Figure 17, C–E). In RE samples, the shared genes were enriched for protein localization to chromatin, cytoskeletal organization, and ribonucleotides biosynthesis (Supplemental Figure 17C), whereas the genes specific to the PZ regions were enriched for glycerophospholipid metabolism, lymphoid progenitor cell differentiation, and axonal guidance–related processes (Supplemental Figure 17D). Furthermore, for the NIZ-related genes, mRNA processing and phosphorylation-related GO-terms were enriched (Supplemental Figure 17E). We then analyzed the total number of genetic variants (INDELs and SNPs) present in SOZ, PZ, and NIZ and found the SOZ harbored more genetic variants compared with PZ and NIZ (Supplemental Figure 15E). These observations suggest that SEEG electrode–derived transcripts can be used to successfully detect genomic variants and complement the matched data on gene expression, DNA methylation, electrophysiology, and radiology from patients with epilepsy.

## Discussion

Unraveling the molecular mechanisms underlying the development and maintenance of the epileptic state presents several challenges due to its multifactorial nature and heterogeneity. Genetic complexity, including polygenic inheritance and gene-environment interactions, complicates the identification of causal factors (40–43). The dynamic nature of epileptogenesis, the interplay between excitatory and inhibitory neurotransmission, and the effects of recurring seizures on brain networks further obscure our understanding (44–46). An incomplete understanding of seizure onset and network recruitment mechanisms and the lack of biomarkers for early diagnosis pose additional obstacles (40, 42, 47, 48). Addressing these challenges requires interdisciplinary collaboration, advanced experimental models and innovative technologies and data integration to decipher the intricate molecular landscape of epilepsy and develop targeted therapies and innovations in diagnosis (49).

Treatment-resistant epilepsies offer a unique opportunity to access the living human brain via analysis of surgically resected material. This has yielded significant advances in understanding brain function and mechanisms of epileptogenesis (50–52). Intracranial EEG recordings from depth electrodes provides a further means of detailed spatial and temporal mapping of epileptogenic tissue, offering valuable insights into seizure onset and propagation. Recent studies have demonstrated the feasibility of extracting nucleic acids from depth electrodes, opening potentially new avenues for molecular profiling of the human brain (23, 37, 53, 54). In this study, we demonstrate MoPEDE, a method to extract nucleic acids from explanted depth electrodes, enabling multimodal profiling of the transcriptome and epigenome in relation to the electrophysiological readings. Through meticulous experimental procedures, including electrode sectioning, nucleic acid extraction, and comprehensive profiling, we demonstrate the feasibility of obtaining high-quality RNA and gDNA from SEEG electrodes, even from deep brain structures. The inclusion of FCD, nonlesional TLE, and RE demonstrates that MoPEDE can, in principle, be applied across spatial scales ranging from highly localized (FCD) through to whole hemisphere (RE). We identified transcripts and DNA methylation profiles that correlate with recorded neurophysiological signals, highlighting the potential relevance of molecular profiling in understanding epilepsy etiology. While the SEEG electrodes covered only selected regions of the brain, surgical outcomes are the gold standard for measuring successful identification and surgical removal of epileptogenic tissue. The excellent surgical outcome, along with accurate ictal recordings, support that the electrodes were in the epileptic foci (Table 1). Additionally, the identification of specific genes and pathways enriched in epileptic brain regions, validated through comparison with publicly available datasets and scRNA-Seq data (24–27), is an important validation of the findings. Our approach also demonstrates the potential to derive information about clinical variants, such as SNPs and INDELs, from a limited number of SEEG electrodes. This includes identifying both pathogenic and risk factor variants across various brain regions affected by epilepsy and their associated functions (22).

While the biological significance of the variation in numbers of differentially expressed genes in relation to EI and etiologies cannot be determined using the small number of cases in the present study, the trace nucleic acids on SEEG contacts may reflect processes that differ in the SOZ and PZ compared with NIZ. This included a number of genes associated with cell metabolism, processes increasingly recognized across the epilepsies. In both TLE and RE samples, we found differences in the SOZ compared with PZ that included transcripts regulating cell death. This could reflect the engagement of pathways that limit neurodegeneration in tissue actively generating seizures, processes known to be evoked by repeated seizures (55). Analysis of SNPs and INDELs within the transcriptome data showed enrichment of many of the same pathways, as well as highlighting RNA processing and chromatin processes that are also implicated in the pathogenesis of epilepsy (56–59). The amount of these variants appears to scale with EI score, implying potential diagnostic applications in distinguishing SOZ from PZ. Furthermore, DNA methylation findings showed alignment with findings from tissue-based studies of FCD and TLE, in terms of numbers of hyper- and hypomethylated genes (60, 61) and biological processes influenced by this epigenetic mark. Correlations between SOZ and PZ suggest that a proportion of epigenetic marks reflect the effects of seizures per se, whereas others may distinguish seizure-triggering sites from recruited networks. Thus, the surface of explanted SEEG contacts appears to bear molecular traces reflective of cellular biology and pathology of the source tissue.

The source of the detected transcripts and epigenetic signals on the electrode surface is likely to be from the surrounding neurons, glia, and perhaps vascular cells at that specific site or along the path of contact. Indeed, we detected transcripts representing multiple resident brain cell types in all 3 cases. Mechanical injury is the likely cause of the release of these nucleic acids due to insertion and/or withdrawal of the electrode, but controlled release is also possible. Indeed, both neurons and glia are capable of releasing packets of membrane-enclosed cellular material in the form of extracellular vesicles (62). While the extent and functional significance of such information-carrying paracrine signaling remains incompletely understood, experimentally evoked seizures have been reported to adjust the abundance and nucleic acid content of extracellular vesicles (63). The coherence between epigenetic and transcript signals suggests these materials had a similar cellular source, but it is possible that origin and release mechanisms differ between the 2 nucleic acid types. The source of some nucleic acids may be from infiltrating immune cells, which deliver such material to resident brain cells, including neurons after seizures (64). While RE is most strongly associated with an infiltrating inflammatory cellular presence, both TLE (65) and FCD (66) feature immune cell infiltration. Further studies may yield a more complete understanding of the basis of the detected signals and their relationship to pathophysiological communication from local and perhaps nonresident cellular sources.

There are a number of limitations to consider in the present study. Foremost, the small number of cases in our proof-of-concept study limits insights into disease mechanisms and etiological factors. Subsequent studies with larger patient numbers will be needed. Obtaining sufficient quantities of high-quality nucleic acids for Next-generation sequencing (NGS) methods from a limited number of SEEG electrodes remains challenging. Indeed, we did not achieve uniform sample integrity, with several samples failing to pass the QC in the NGS workflow. This may be addressed by further improvements in the workflow such as reducing the time to snap-freezing electrodes or proceeding to immediate extraction of nucleic acids. Additional or new extraction methods may better ensure the preservation of sample integrity. Furthermore, other information-containing material may also be recoverable that would complement MoPEDE — for example, histone modifications, which play a major role in controlling gene expression and cellular identity (47, 67).

Another caveat of the present study is that the implantation or explantation procedure may result in some gliosis as well as the carriage of nucleic acid material from one site to another, thereby obscuring the origin of the signal. A detailed evaluation of the extent of such material transfer and its effect on molecular information will be required in future studies. Also, our study also relied on comparisons with publicly available datasets that include data derived from postmortem samples. Future studies could explore matching explanted electrode fingerprints to findings in matched postresection tissue samples. Indeed, recent studies comparing the transcriptome profiles of fresh, frozen, and formalin-fixed, paraffin-embedded (FFPE) tissues have demonstrated that both frozen and FFPE tissues can be used to retrieve certain information on gene expression, provided proper QC measures are in place (68, 69).

It is important to note that SEEG has an inherent sampling bias with low spatial resolution and is able to cover only a limited area of the brain or epileptic network. Increasing numbers of depth electrodes must be balanced with limiting intraoperative and postoperative complications of multiple-depth electrode placement. Visual inspection of SEEG data is open to reader variability and, while still the clinical standard, has prompted the development of more automated techniques. EI scores, as used here, are more accurate with fast frequencies at seizure onset but are not as accurate with slower frequencies. For example, the temporal pole SOZ in patient B had a slow ictal onset frequency with a low EI score. EI does not uniformly correlate with visual inspection. For example, EI scores in the RE case were diffusely elevated, including in the NIZ, likely due to the expected diffuse epileptogenicity in the affected hemisphere.

In conclusion, we describe a multimodal methodology that has the potential to provide insights into disease mechanisms and prospects for improving the diagnosis and treatment of epilepsy. The ability to extract nucleic acids from depth electrodes provides a noninvasive means of molecular profiling, offering potential applications beyond epilepsy, such as studying other neurological disorders. Future research directions may involve exploring additional nucleic acids (e.g., long noncoding RNA, small RNAs), epitranscriptome changes (RNA modifications), and epigenetic marks (histone marks) obtained from depth electrodes as well as investigating the applicability of this approach across diverse epilepsy subtypes.

## Methods

### Sex as a biological variable.

This study examined data from both male (*n* = 1) and female (*n* = 2) individuals. Due to the small number of patients, the primary analyses in this study did not consider sex as a biological variable.

### Neurophysiology data collection.

All participants underwent robot-assisted implantation of intracranial depth electrodes and SEEG monitoring in the Epilepsy Monitoring Unit in Beaumont Hospital Dublin, Ireland. Depth electrodes (DIXI Medical) were implanted according to the preoperative hypothesis of the SOZ. The anatomic location of each electrode was confirmed by postoperative CT coregistered with preoperative volumetric MRI (Figure 2). Continuous EEG was recorded at 1,024 Hz with concurrent video recording (Xltek EEG System, Natus Inc.). Two experienced clinical neurophysiologists/epileptologists performed visual inspection of seizures and classified each cortical region as part of the SOZ, PZ, or NIZ, based on summation of ictal EEG data. The SOZ is defined as the electrodes involved at the onset of the EEG seizure and correlates with an EI > 0.4. The PZ is defined as the electrodes involved in early seizure propagation within 10 seconds and correlates with an EI of 0.2–0.4. The NIZ is defined as electrodes not involved in the seizure and correlates with an EI < 0.2.

To measure EI, EEG was analyzed using the AnyWave software (Marseille) (28, 29). The EI measures the change in energy ratio and time delay from electrode contacts at seizure onset to estimate the epileptogenicity of a given cortical region. Representative seizures from each patient that occurred over 48 hours from electrode implantation and with minimal artifact were selected. Bipolar contacts in gray matter at the mesial or lateral point of each depth electrode represented each brain region under investigation. Contacts in white matter or outside of the brain were excluded.

The depth electrodes were explanted under general anesthesia following standard clinical procedures. They were immediately placed in sterile, RNA, DNA, and nuclease-free 15 mL tubes and frozen in dry ice for transport. The electrodes were then stored at –80°C until further processing.

### Patient clinical characteristics.

Table 1 summarizes information on the patients. Patient A is a 49-year-old male who underwent SEEG monitoring for medication-resistant focal epilepsy due to a right parietal FCD. Thirty-three electroclinical seizures were recorded. The EEG onset for all seizures arose from the lateral contacts of the X electrode, at the parietal FCD. Seizure propagation involved the supplementary motor area and temporo-occipital junction. The patient underwent resection of the FCD and achieved an Engel class 1A (70) outcome, remaining seizure free at 1 year. Histopathology confirmed a type IIa FCD.

Patient B is a 40-year-old female with MRI-negative, medication-resistant TLE who underwent SEEG monitoring. Six electroclinical seizures were recorded. EEG onset was from the anterior hippocampus and the temporal pole. Seizure propagation involved the middle temporal gyrus and inferior temporal gyrus. The patient underwent a left temporal lobectomy with amygdalohippocampectomy, resulting in an Engel class 2A outcome at 1 year, and was initially seizure free but experiencing rare seizures subsequently. Histopathology revealed Chaslin’s subpial gliosis.

Patient C is a 24-year-old female who was diagnosed with RE at the age of 15 years and has been treated medically. Due to persistent medication-resistant epilepsy, she underwent SEEG monitoring. Thirty-two electroclinical seizures were recorded. Ictal onset of most seizures was from the hippocampus and the gyrus rectus. Seizure propagation involved the cingulate and frontal operculum. The patient underwent right frontal and anterior temporal resection with an Engel class 2A outcome at 1 year; the patient was initially seizure free with rare seizures subsequently. Initial brain biopsy at age 15 years showed chronic encephalitis with T cell–rich perivascular and parenchymal inflammation. Histopathology from the resection 8 years later revealed severe hippocampal sclerosis and cortical gliosis, with no active inflammation (Table 1).

### Total nucleic acid extraction.

For the total nucleic acid extraction, snap-frozen SEEG electrodes were cut into small pieces using sterile nuclease-free scissors into microfuge tubes, and the total nucleic acids were extracted using PicoPure RNA Isolation Kit (Thermo Fisher Scientific, KIT0204) according to the manufacturer’s instructions with minor modifications. Briefly, 50 to 100 μL (up to the electrode pieces were completely immersed) extraction buffer was added to the cut electrodes followed by 30 minutes of incubation at 42°C. Meanwhile, the column was preconditioned with 250 μL of conditioning buffer. An equal amount (50–100 μL) of 70% ethanol was added to the extracted samples and mixed, transferred to the preconditioned column centrifuge for 2 minutes at 100*g*, immediately followed by centrifugation at 16,000*g* for 30 seconds to remove flowthrough. Bound fractions were washed with wash buffer I without DNase to retain the gDNA in the same samples followed by wash buffer II and the samples were eluted with 11 μL of elution buffer.

Samples were quantified in a NanoDrop, and the quality of the samples was analyzed using an Agilent TapeStation system with high-sensitivity RNA tapes or in a fragment analyzer. For the separation of DNA and RNA, the eluted samples were divided into equal portions. One portion was used for RNA isolation and the other was used for DNA isolation.

### RNA purification.

For the RNA purification, samples were first treated with DNase I (Thermo Fisher Scientific, EN0521) to remove the DNA and purified using GeneJET RNA Purification Kit (Thermo Fisher Scientific, K0732) according to the manufacturer’s instructions. Purified RNA samples were quantified using Qubit high-sensitivity RNA Quantification assay (Thermo Fisher Scientific, Q32852). The quality of the RNA was analyzed using Agilent TapeStation systems with high-sensitivity RNA tapes with RINs.

### gDNA purification.

For gDNA purification, samples were added to nuclease-free water (Thermo Fisher Scientific, AM9939) to the final volume of 100 μL. Then 3 μL of RNase A (Thermo Fisher Scientific, R1253) was added to the samples and incubated for 20 minutes at 37°C. Further DNA was purified using Monarch Genomic DNA Purification Kit (NEB, T3010S) according to the manufacturer’s instructions with minor modifications. A total of 1 μL of Proteinase K (Thermo Fisher Scientific, EO0491) was added to the samples, and 100 μL of lysis buffer was added and incubated for 5 minutes at 57°C. Then 400 μL of binding buffer was added and loaded to the purification columns, washed twice with wash buffer, and eluted with 15 μL of elution buffer. Purified DNA samples were quantified using Qubit high-sensitivity dsDNA Quantification assay (Thermo Fisher Scientific, Q32854). The quality of the gDNA was analyzed using an Agilent TapeStation system with high-sensitivity gDNA tapes.

### Bulk FLASH-Seq.

Following extraction, RNA samples were normalized to 1 ng/μL, and 1 μL of RNA was input to prepare RNA libraries using a bulk input optimized FLASH-Seq protocol. In brief, RNA was converted to cDNA fragments using Maxima H Minus (Thermo Fisher Scientific, EP0753) and amplified with KAPA HiFi HotStart (Roche, KK2602). Note, double lysis mix volume used and 4 μL RTPCR mix used to account for larger wells in 96-well plates. cDNA was then cleaned using a 0.8× ratio of homebrew SeraMag beads in 18% PEG (Cytivia, GE24152105050250). cDNA concentrations and sizings were checked using Qubit (Thermo Fisher Scientific, Q33231) and Agilent Bioanalyzer (Agilent, 5067-4626). cDNA were normalized to 200 pg/μL before tagmentation using 0.2 μM of homemade Tn5 (EPFL). The reaction was halted with 0.2% SDS. Indexing PCR was performed to add Nextera index adapters (1 μM final, Integrated DNA Technology) using KAPA HiFi reagents (KK2102, Roche). Libraries were pooled in equal volumes, and a final 0.8× cleanup was performed with homebrew SeraMag beads before measuring the sample concentration and sizing. The library pool was normalized and sequenced on Illumina NovaSeq SP flow cell (catalog 20040719) at approximately 25 million reads/sample. Basecalling and demultiplexing were performed with bcl2fastq (v2.20, Illumina).

### Transcriptome quality check, mapping, quantification, and variant analysis.

Adapter sequences were removed from raw FASTQ files using Cutadapt (v4.9) (71). Subsequently, high-quality, adapter-trimmed reads were mapped against the human reference genome (GRCh38) using the STARlong utility within the STAR aligner (72). Transcriptome assembly and quantification of gene expression was performed using StringTie2 at default parameters for each electrode from different brain regions in each case (73). Then we performed differential gene expression for all regions and epilepsy brains using DESeq2 (74). We incorporated publicly available scRNA-Seq data from patients with epilepsy (27), into our analysis. Quality checks, data reduction, and integration of scRNA-Seq data from healthy controls and patients with epilepsy were performed using Seurat (v4) (75, 76). To identify variants using transcriptome data, we employed the GATK (v4.5.0.0) RNA-Seq short variant discovery (SNPs + INDELs) pipeline (75). The process began by marking duplicate reads with MarkDuplicates. Next, we used SplitNCigarReads to split reads with N in the CIGAR string into multiple supplementary alignments and hard clip mismatching overhangs. We then used AddOrReplaceReadGroups (Picard) to assign all reads in a file to a single new-read group. Variants were called using HaplotypeCaller, followed by high-quality variant filtering with VariantFiltration, applying filters at quality by depth (QD) < 2.0, FS > 60.0, MQ (number of reads supporting the variants) < 30.0, DP (reads coverage at variant site) < 20.0, and quality score (QUAL) < 20.0 (77). Variants effect was determined using Ensembl Variant Effect Predictor (VEP) for high-quality variants (78). Furthermore, we intersected the variants present in all replicate for a region in epilepsy brains using BCFtools (79), and we annotated them using wANNOVAR (80).

### Methylation quality check, mapping, and differential analysis.

We performed quality checks using FastQC to assess the overall quality of the bisulfite-converted sequencing (BS-Seq) reads. Following QC, adapter sequences were trimmed using TrimGalore. The human reference genome (GRCh38) was prepared using the bismark_genome_preparation utility from Bismark to account for bisulfite conversion. Subsequently, high-quality, adapter-trimmed reads were mapped to the prepared reference genome using Bismark (v0.22.3). Methylated cytosines were extracted from the mapped reads using MethylDackel, considering all 3 methylation contexts: CpG, CHG, and CHH (dpryan79/MethylDackel: a nearly universal methylation extractor for BS-Seq experiments GitHub repository). DMRs were identified using methylKit with a sliding window approach (window size = 200 bp, step size = 200 bp, minimum coverage = 10 bp, difference ≥ 5 and ≤ –5, and *q* ≤ 0.05) using default parameters. Methylated regions were further annotated using the bedtools intersect utility, to identify overlapping genes. Finally, we integrated methylation information at the gene level with their corresponding transcriptional expression data.

### Statistics.

We used mapped BAM files to generate BigWig files for visualization of transcriptome, and we used methylome density in gene bodies and flanking regions using deepTools (81), various utilities available within SAMtools, to prepare the file inputs for different analyses (82). Statistical analyses and visualizations were performed using R packages (R Core Team, 2023). Finally, BioRender was used to create schematics and enhance the visual presentation of our figures.

### Study approval.

The present study was reviewed and approved by the Beaumont Hospital Medical Research Ethics Committee under study no. 20.58. All patients provided written informed consent.

### Data availability.

We have submitted raw fastq and count matrix for transcriptome and methylome data generated in this study to Gene Expression Omnibus (GEO) using accession nos. GSE268714 and GSE268715. Values for all data points in graphs are reported in the Supporting Data Values file.

## Author contributions

AKD performed data analysis and wrote the manuscript. AM performed the experiments and molecular analysis of samples and wrote the manuscript; AS and JL participated in sample procurement, neurophysiology, and clinical data collection and analysis. KJS and DFO performed SEEG implantation procedures and surgical resections and obtained consent. PWW oversaw and interpreted the clinical, neurophysiological, radiological, and pathological data collection and analysis and edited the manuscript. RAS and SP performed the FLASH-Seq procedure on SEEG-derived RNA. VKT and DCH coconceived the study, obtained funding, supervised the study, performed data interpretation, and coedited the manuscript.

## Supplementary Material

Supplemental data

Supplemental tables 1-34

Supporting data values

## Figures and Tables

**Table 1:**
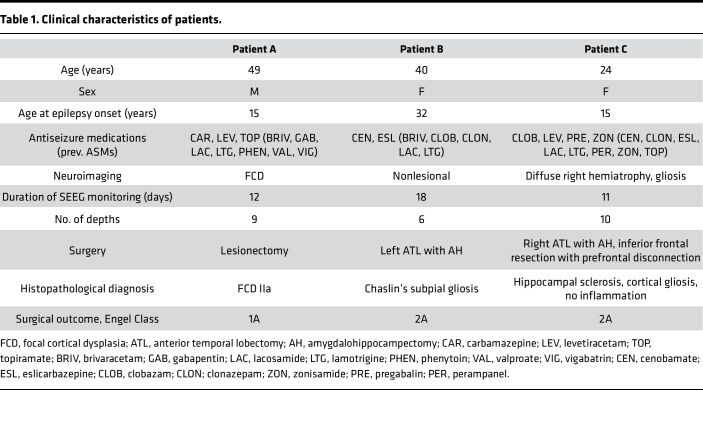
Clinical characteristics of patients.

## Version 1 11/14/2024

In-Press Preview

## Version 2 01/09/2025

Electronic publication
