# Supplementary material for: High-resolution multimodal profiling of human epileptic brain activity via explanted depth electrodes
Source: JCI Insight. 2025 Jan 9;10(1):e184518. doi: 10.1172/jci.insight.184518 (PMC11721296; doi:10.1172/jci.insight.184518)
Supplement: Supplemental data [file jciinsight-10-184518-s115.pdf]

Figure S1

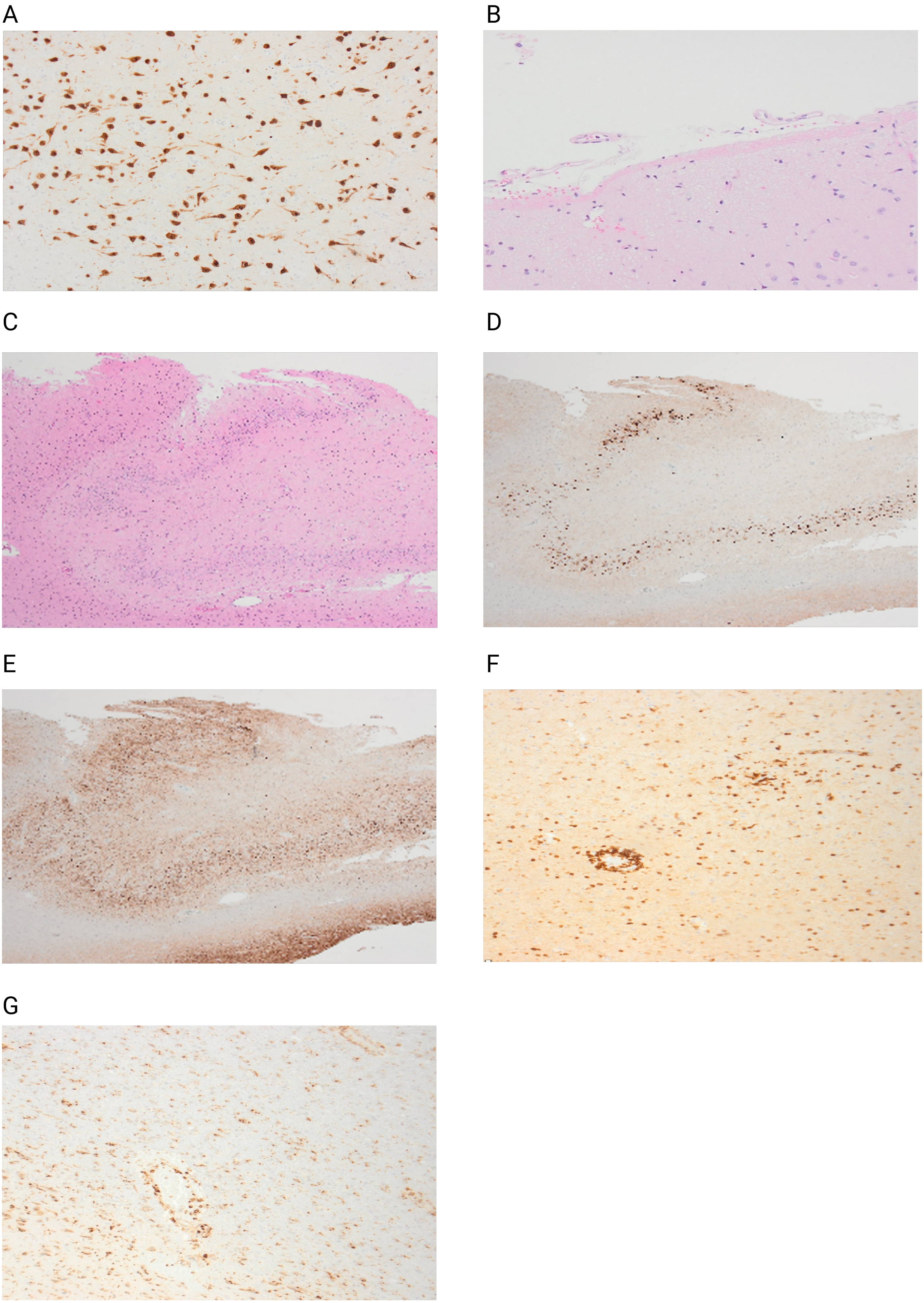

Figure S2

A.

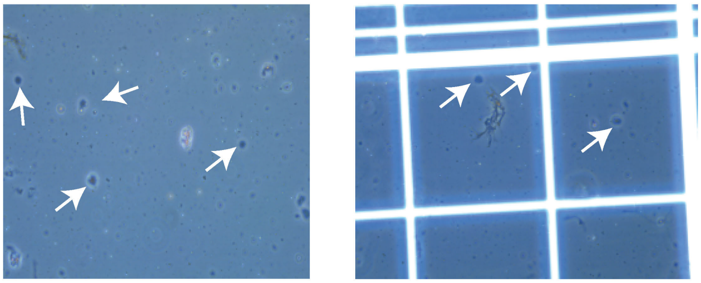

C.

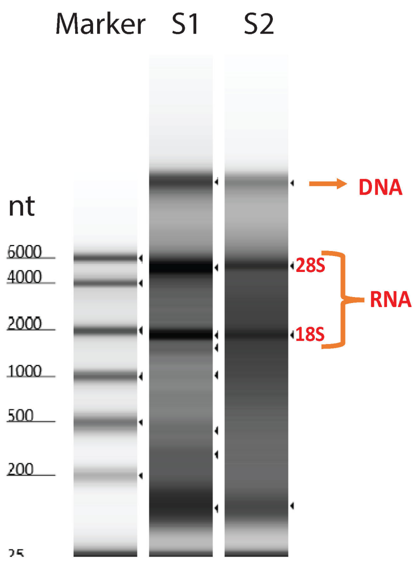

B.

Number of cut pieces	Concentration (ng)	A260/280	A260/230
12	84	1.98	1.08
6	31.1	1.86	0.74
4	34.8	1.87	0.23
3	35	1.83	1.03
2	13.9	1.58	0.35

D.

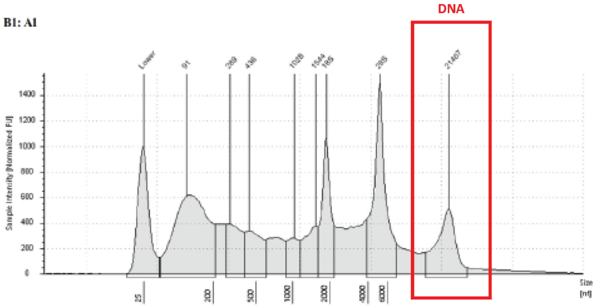

E.

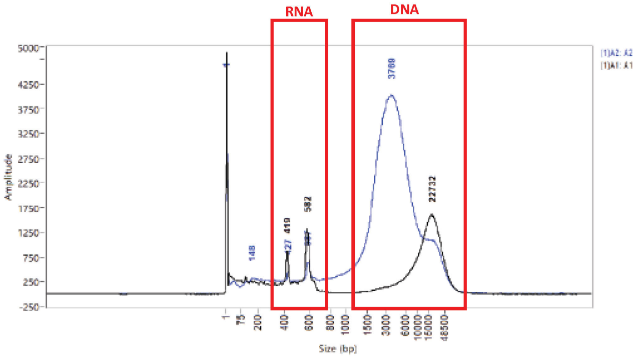

F.

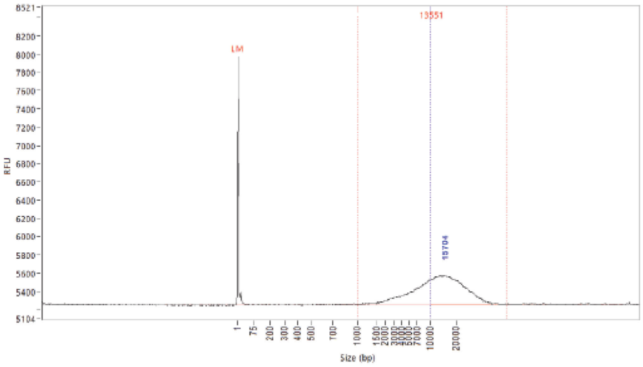

G.

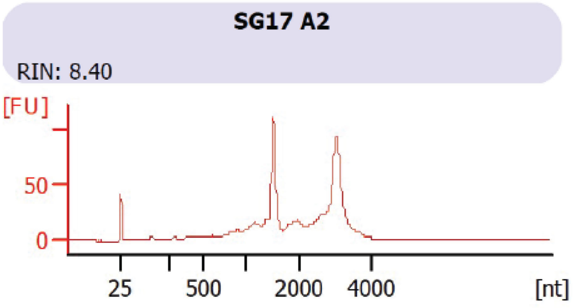

Figure S3

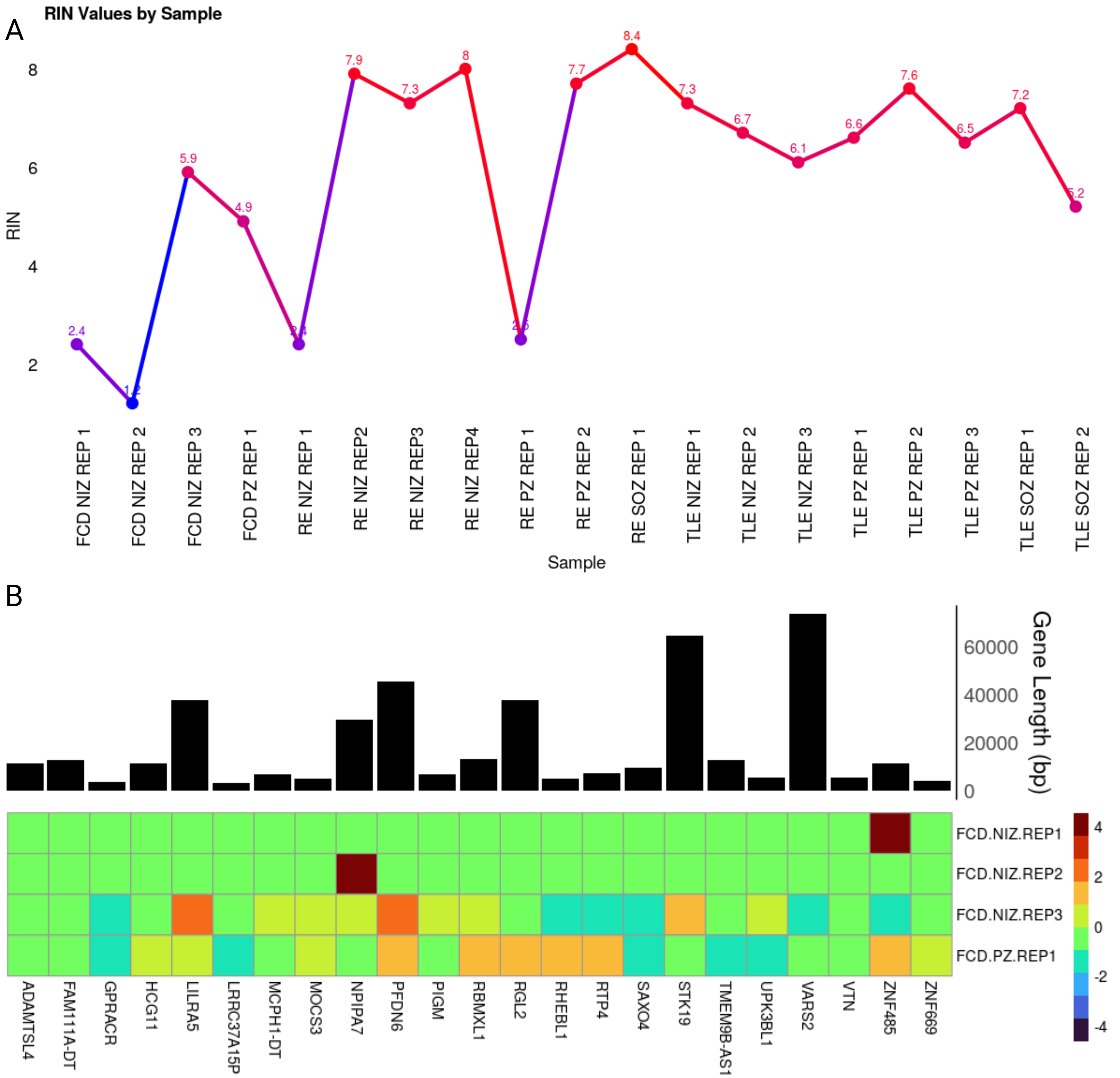
A
RIN Values by Sample
RIN
8
6
4
2
2.4
1.2
5.9
4.9
7.9
7.3
8
7.7
8.4
7.3
6.7
6.1
6.6
7.6
6.5
7.2
FCD NIZ REP 1
FCD NIZ REP 2
FCD NIZ REP 3
FCD PZ REP 1
RE NIZ REP 1
RE NIZ REP2
RE NIZ REP3
RE NIZ REP4
RE PZ REP 1
RE PZ REP 2
RE SOZ REP 1
TLE NIZ REP 1
TLE NIZ REP 2
TLE NIZ REP 3
TLE PZ REP 1
TLE PZ REP 2
TLE PZ REP 3
TLE SOZ REP 1
TLE SOZ REP 2
Sample
B
Gene Length (bp)
60000
40000
20000
0
FCD.NIZ.REP1
FCD.NIZ.REP2
FCD.NIZ.REP3
FCD.PZ.REP1
4
2
0
-2
-4
ADAMTSL4
FAM111A-DT
GPRACR
HCG11
LILRA5
LRRC37A15P
MCPH1-DT
MOCS3
NPIPA7
PFDN6
PIGM
RBMXL1
RGL2
RHEBL1
RTP4
SAXO4
STK19
TMEM9B-AS1
UPK3BL1
VARS2
VTN
ZNF485
ZNF669

Figure S4

A

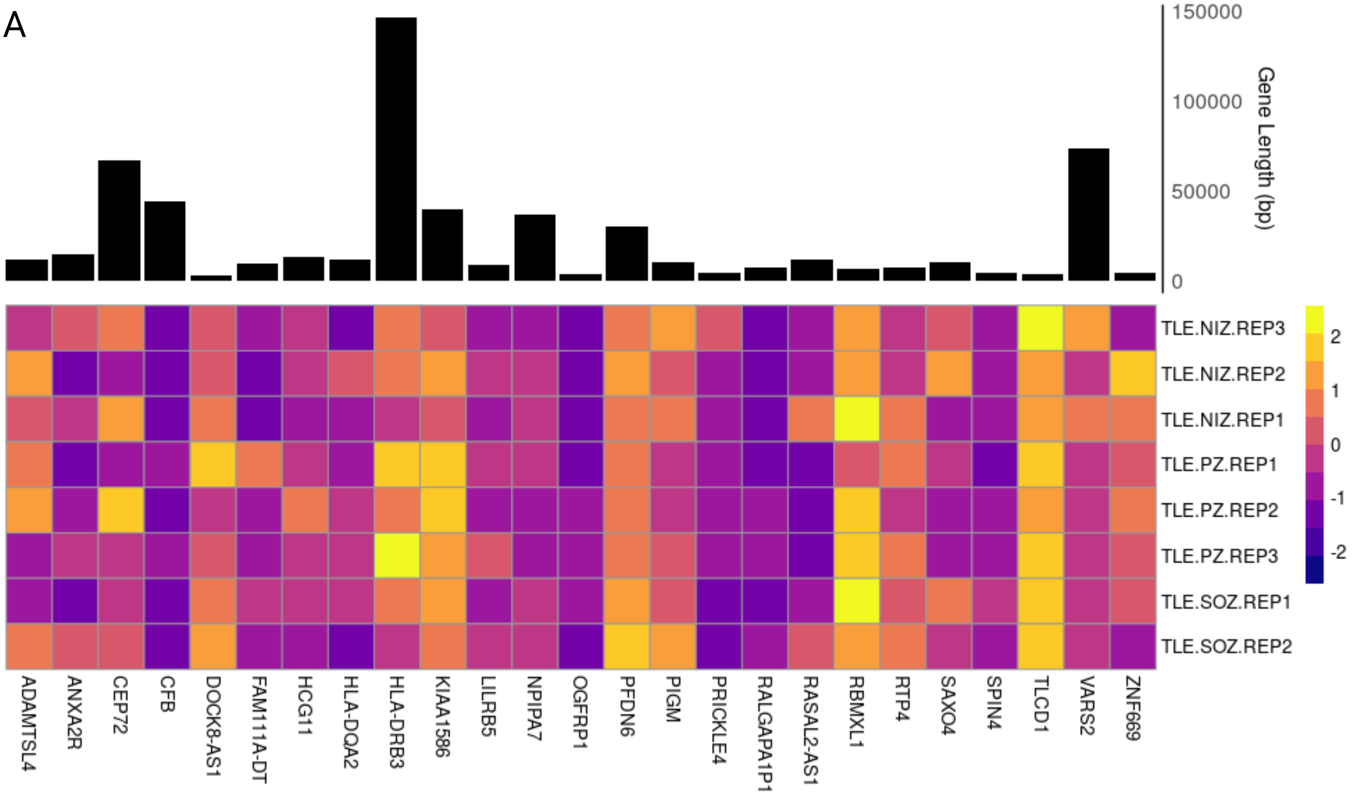
150000
100000
50000
0
Gene Length (bp)
TLE.NIZ.REP3
TLE.NIZ.REP2
TLE.NIZ.REP1
TLE.PZ.REP1
TLE.PZ.REP2
TLE.PZ.REP3
TLE.SOZ.REP1
TLE.SOZ.REP2
2
1
0
-1
-2
ADAMTSL4
ANXA2R
CEP72
CFB
DOCK8-AS1
FAM111A-DT
HCG11
HLA-DQA2
HLA-DRB3
KIAA1586
LILRB5
NPIPA7
OGFRP1
PFDN6
PIGM
PRICKLE4
RALGAPA1P1
RASAL2-AS1
RBMXL1
RTP4
SAXO4
SPIN4
TLCD1
VARS2
ZNF669

B

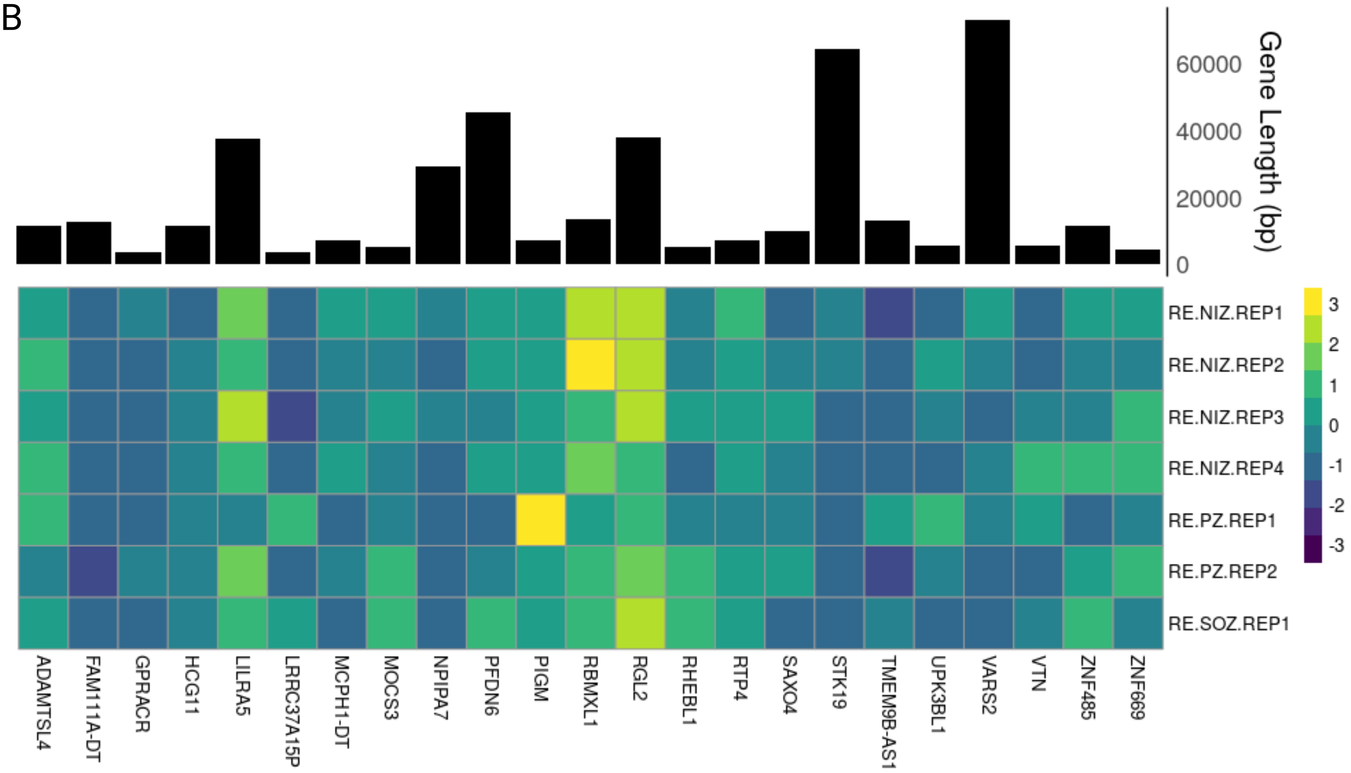
60000
40000
20000
0
Gene Length (bp)
RE.NIZ.REP1
RE.NIZ.REP2
RE.NIZ.REP3
RE.NIZ.REP4
RE.PZ.REP1
RE.PZ.REP2
RE.SOZ.REP1
3
2
1
0
-1
-2
-3
ADAMTSL4
FAM111A-DT
GPRACR
HCG11
LILRA5
LRRC37A15P
MCPH1-DT
MOCS3
NPIPA7
PFDN6
PIGM
RBMXL1
RGL2
RHEBL1
RTP4
SAXO4
STK19
TMEM9B-AS1
UPK3BL1
VARS2
VTN
ZNF485
ZNF669

Figure S5

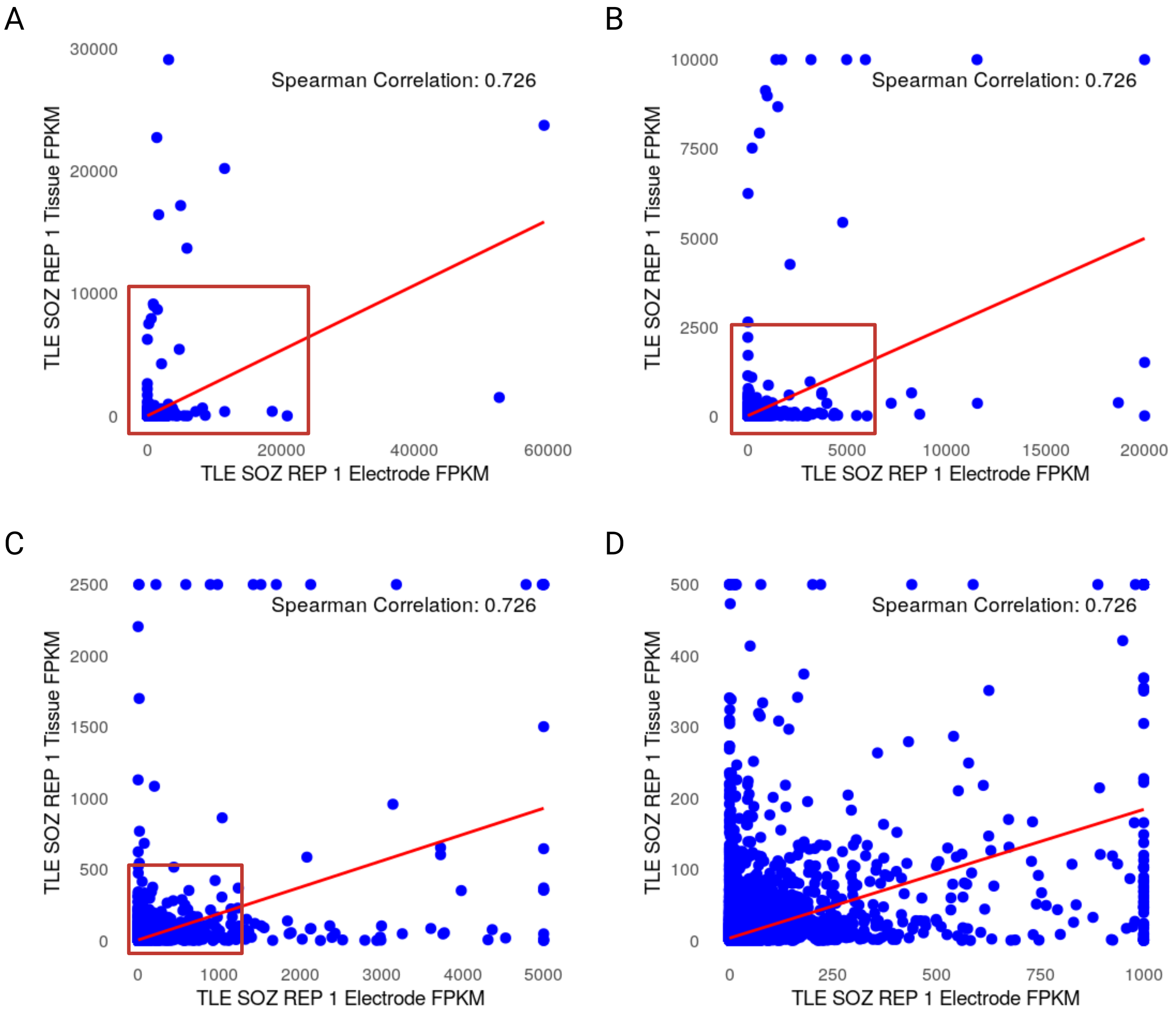
A
Spearman Correlation: 0.726
TLE SOZ REP 1 Tissue FPKM
TLE SOZ REP 1 Electrode FPKM
0
10000
20000
30000
0
20000
40000
60000
B
Spearman Correlation: 0.726
TLE SOZ REP 1 Tissue FPKM
TLE SOZ REP 1 Electrode FPKM
0
2500
5000
7500
10000
0
5000
10000
15000
20000
C
Spearman Correlation: 0.726
TLE SOZ REP 1 Tissue FPKM
TLE SOZ REP 1 Electrode FPKM
0
500
1000
1500
2000
2500
0
1000
2000
3000
4000
5000
D
Spearman Correlation: 0.726
TLE SOZ REP 1 Tissue FPKM
TLE SOZ REP 1 Electrode FPKM
0
100
200
300
400
500
0
250
500
750
1000

Figure S6

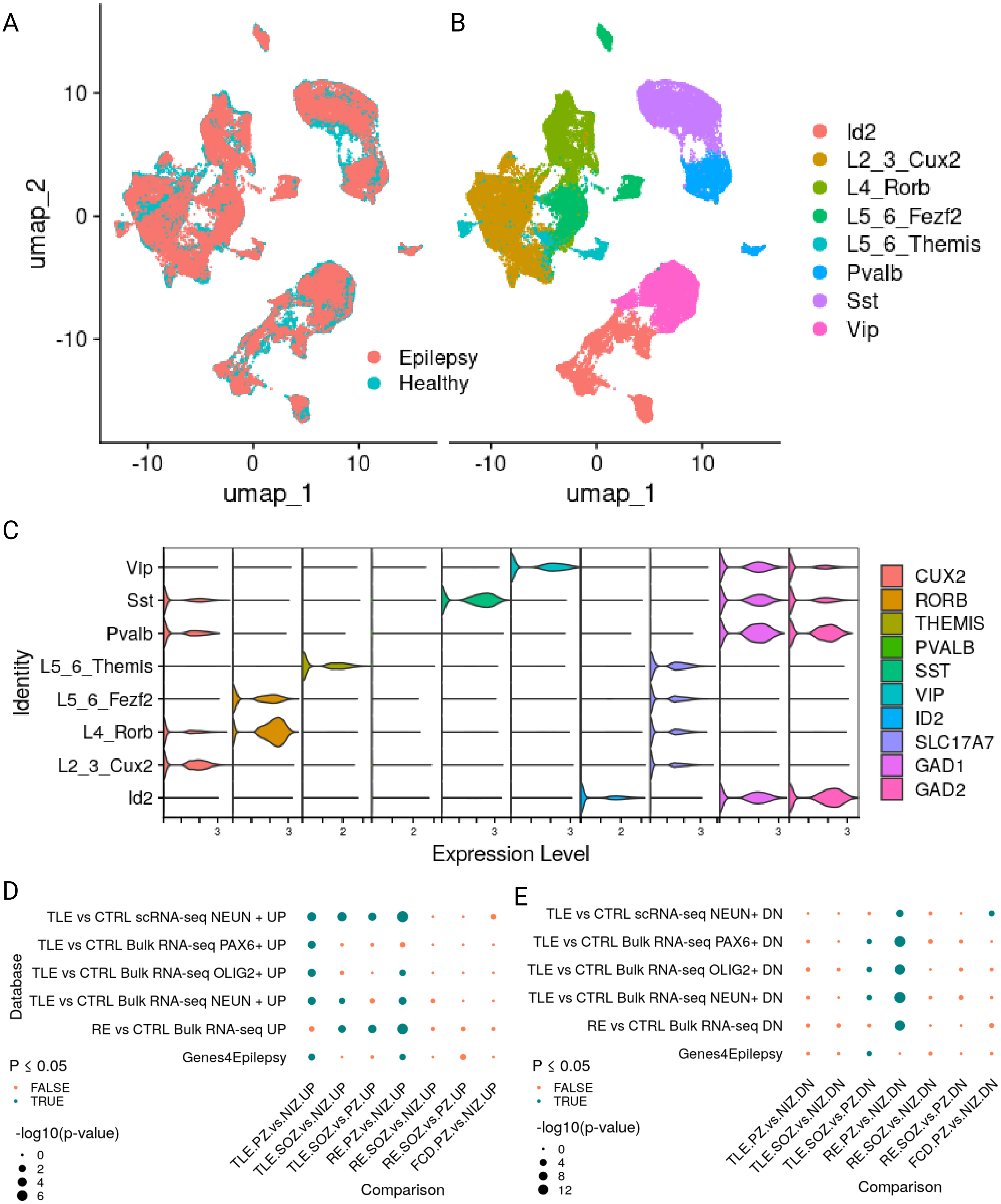

A
B
umap_2
umap_1
Epilepsy
Healthy
Id2
L2_3_Cux2
L4_Rorb
L5_6_Fezf2
L5_6_Themis
Pvalb
Sst
Vip
C
Identity
Expression Level
CUX2
RORB
THEMIS
PVALB
SST
VIP
ID2
SLC17A7
GAD1
GAD2
D
TLE vs CTRL scRNA-seq NEUN + UP
TLE vs CTRL Bulk RNA-seq PAX6+ UP
TLE vs CTRL Bulk RNA-seq OLIG2+ UP
TLE vs CTRL Bulk RNA-seq NEUN + UP
RE vs CTRL Bulk RNA-seq UP
Genes4Epilepsy
Database
P ≤ 0.05
FALSE
TRUE
-log10(p-value)
TLE.PZ.vs.NIZ.UP
TLE.SOZ.vs.NIZ.UP
TLE.SOZ.vs.PZ.UP
RE.PZ.vs.NIZ.UP
RE.SOZ.vs.NIZ.UP
RE.SOZ.vs.PZ.UP
FCD.PZ.vs.NIZ.UP
Comparison
E
TLE vs CTRL scRNA-seq NEUN+ DN
TLE vs CTRL Bulk RNA-seq PAX6+ DN
TLE vs CTRL Bulk RNA-seq OLIG2+ DN
TLE vs CTRL Bulk RNA-seq NEUN+ DN
RE vs CTRL Bulk RNA-seq DN
Genes4Epilepsy
P ≤ 0.05
FALSE
TRUE
-log10(p-value)
TLE.PZ.vs.NIZ.DN
TLE.SOZ.vs.NIZ.DN
TLE.SOZ.vs.PZ.DN
RE.PZ.vs.NIZ.DN
RE.SOZ.vs.NIZ.DN
RE.SOZ.vs.PZ.DN
FCD.PZ.vs.NIZ.DN
Comparison

Figure S7

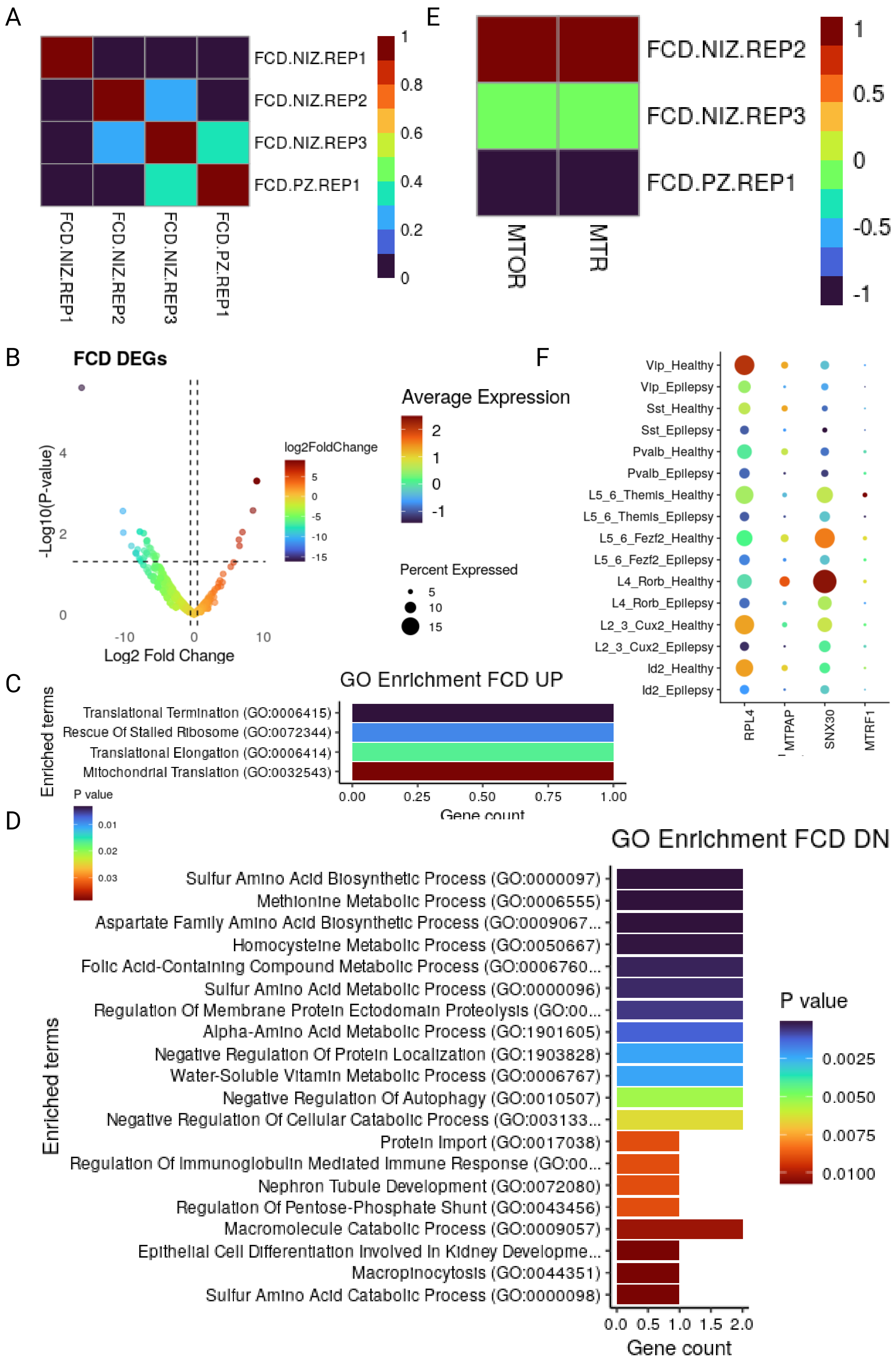

A
FCD.NIZ.REP1
FCD.NIZ.REP2
FCD.NIZ.REP3
FCD.PZ.REP1
1
0.8
0.6
0.4
0.2
0
E
MTOR
MTR
1
0.5
0
-0.5
-1
B
FCD DEGs
-Log10(P-value)
Log2 Fold Change
log2FoldChange
F
Average Expression
Percent Expressed
Vip_Healthy
Vip_Epilepsy
Sst_Healthy
Sst_Epilepsy
Pvalb_Healthy
Pvalb_Epilepsy
L5_6_Themis_Healthy
L5_6_Themis_Epilepsy
L5_6_Fezf2_Healthy
L5_6_Fezf2_Epilepsy
L4_Rorb_Healthy
L4_Rorb_Epilepsy
L2_3_Cux2_Healthy
L2_3_Cux2_Epilepsy
Id2_Healthy
Id2_Epilepsy
RPL4
MTPAP
SNX30
MTRF1
C
GO Enrichment FCD UP
Enriched terms
Translational Termination (GO:0006415)
Rescue Of Stalled Ribosome (GO:0072344)
Translational Elongation (GO:0006414)
Mitochondrial Translation (GO:0032543)
Gene count
P value
D
GO Enrichment FCD DN
Enriched terms
Sulfur Amino Acid Biosynthetic Process (GO:0000097)
Methionine Metabolic Process (GO:0006555)
Aspartate Family Amino Acid Biosynthetic Process (GO:0009067...
Homocysteine Metabolic Process (GO:0050667)
Folic Acid-Containing Compound Metabolic Process (GO:0006760...
Sulfur Amino Acid Metabolic Process (GO:0000096)
Regulation Of Membrane Protein Ectodomain Proteolysis (GO:00...
Alpha-Amino Acid Metabolic Process (GO:1901605)
Negative Regulation Of Protein Localization (GO:1903828)
Water-Soluble Vitamin Metabolic Process (GO:0006767)
Negative Regulation Of Autophagy (GO:0010507)
Negative Regulation Of Cellular Catabolic Process (GO:003133...
Protein Import (GO:0017038)
Regulation Of Immunoglobulin Mediated Immune Response (GO:00...
Nephron Tubule Development (GO:0072080)
Regulation Of Pentose-Phosphate Shunt (GO:0043456)
Macromolecule Catabolic Process (GO:0009057)
Epithelial Cell Differentiation Involved In Kidney Developme...
Macropinocytosis (GO:0044351)
Sulfur Amino Acid Catabolic Process (GO:0000098)
Gene count
P value

Figure S8

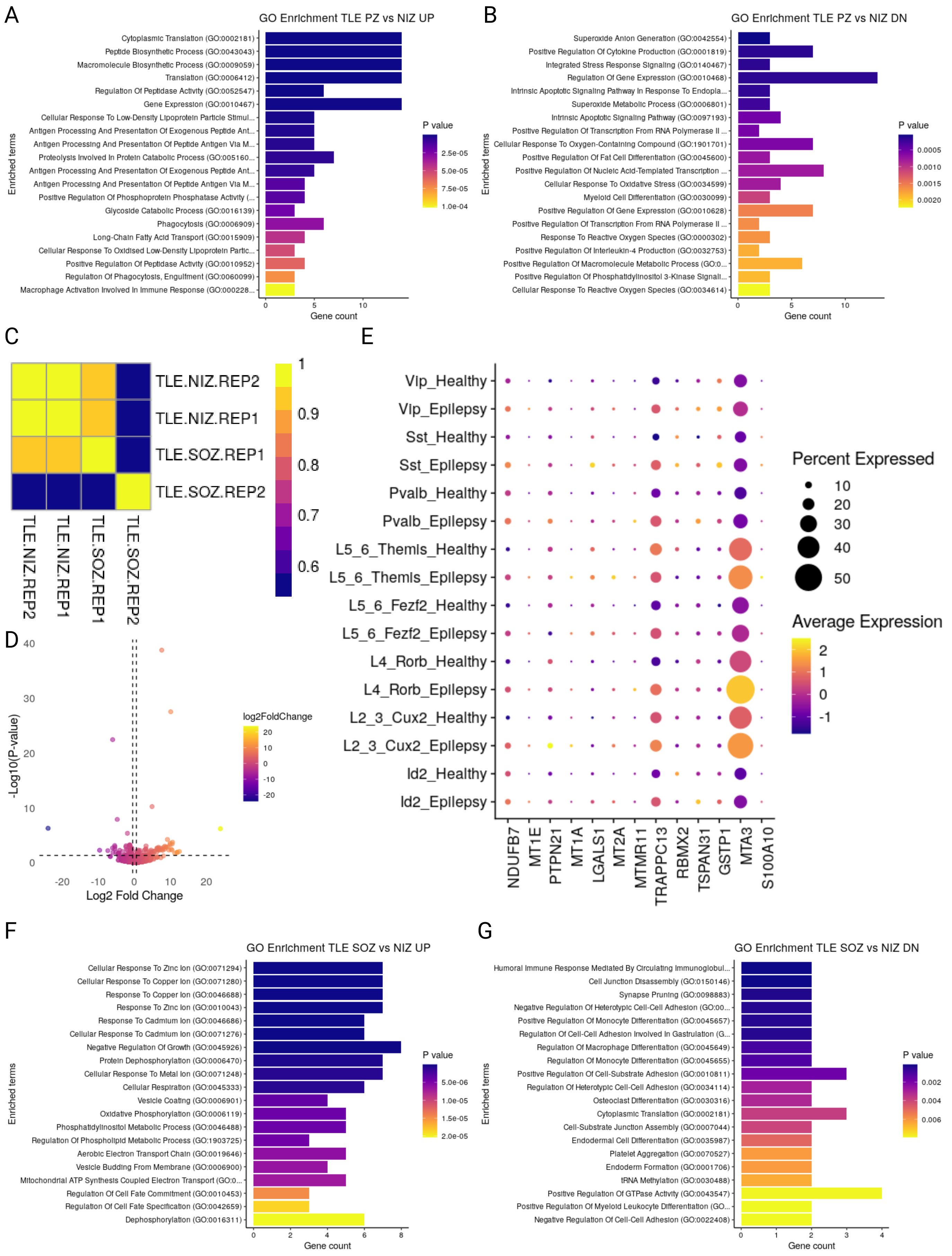
A
GO Enrichment TLE PZ vs NIZ UP
Cytoplasmic Translation (GO:0002181)
Peptide Biosynthetic Process (GO:0043043)
Macromolecule Biosynthetic Process (GO:0009059)
Translation (GO:0006412)
Regulation Of Peptidase Activity (GO:0052547)
Gene Expression (GO:0010467)
Cellular Response To Low-Density Lipoprotein Particle Stimul...
Antigen Processing And Presentation Of Exogenous Peptide Ant...
Antigen Processing And Presentation Of Peptide Antigen Via M...
Proteolysis Involved In Protein Catabolic Process (GO:005160...
Antigen Processing And Presentation Of Exogenous Peptide Ant...
Antigen Processing And Presentation Of Peptide Antigen Via M...
Positive Regulation Of Phosphoprotein Phosphatase Activity (...
Glycoside Catabolic Process (GO:0016139)
Phagocytosis (GO:0006909)
Long-Chain Fatty Acid Transport (GO:0015909)
Cellular Response To Oxidised Low-Density Lipoprotein Partic...
Positive Regulation Of Peptidase Activity (GO:0010952)
Regulation Of Phagocytosis, Engulfment (GO:0060099)
Macrophage Activation Involved In Immune Response (GO:000228...
Enriched terms
Gene count
P value
2.5e-05
5.0e-05
7.5e-05
1.0e-04
B
GO Enrichment TLE PZ vs NIZ DN
Superoxide Anion Generation (GO:0042554)
Positive Regulation Of Cytokine Production (GO:0001819)
Integrated Stress Response Signaling (GO:0140467)
Regulation Of Gene Expression (GO:0010468)
Intrinsic Apoptotic Signaling Pathway In Response To Endopla...
Superoxide Metabolic Process (GO:0006801)
Intrinsic Apoptotic Signaling Pathway (GO:0097193)
Positive Regulation Of Transcription From RNA Polymerase II ...
Cellular Response To Oxygen-Containing Compound (GO:1901701)
Positive Regulation Of Fat Cell Differentiation (GO:0045600)
Positive Regulation Of Nucleic Acid-Templated Transcription ...
Cellular Response To Oxidative Stress (GO:0034599)
Myeloid Cell Differentiation (GO:0030099)
Positive Regulation Of Gene Expression (GO:0010628)
Positive Regulation Of Transcription From RNA Polymerase II ...
Response To Reactive Oxygen Species (GO:0000302)
Positive Regulation Of Interleukin-4 Production (GO:0032753)
Positive Regulation Of Macromolecule Metabolic Process (GO:0...
Positive Regulation Of Phosphatidylinositol 3-Kinase Signali...
Cellular Response To Reactive Oxygen Species (GO:0034614)
Enriched terms
Gene count
P value
0.0005
0.0010
0.0015
0.0020
C
TLE.NIZ.REP2
TLE.NIZ.REP1
TLE.SOZ.REP1
TLE.SOZ.REP2
1
0.9
0.8
0.7
0.6
D
-Log10(P-value)
Log2 Fold Change
log2FoldChange
E
Vip_Healthy
Vip_Epilepsy
Sst_Healthy
Sst_Epilepsy
Pvalb_Healthy
Pvalb_Epilepsy
L5_6_Themis_Healthy
L5_6_Themis_Epilepsy
L5_6_Fezf2_Healthy
L5_6_Fezf2_Epilepsy
L4_Rorb_Healthy
L4_Rorb_Epilepsy
L2_3_Cux2_Healthy
L2_3_Cux2_Epilepsy
Id2_Healthy
Id2_Epilepsy
NDUFB7
MT1E
PTPN21
MT1A
LGALS1
MT2A
MTMR11
TRAPPC13
RBMX2
TSPAN31
GSTP1
MTA3
S100A10
Percent Expressed
Average Expression
F
GO Enrichment TLE SOZ vs NIZ UP
Cellular Response To Zinc Ion (GO:0071294)
Cellular Response To Copper Ion (GO:0071280)
Response To Copper Ion (GO:0046688)
Response To Zinc Ion (GO:0010043)
Response To Cadmium Ion (GO:0046686)
Cellular Response To Cadmium Ion (GO:0071276)
Negative Regulation Of Growth (GO:0045926)
Protein Dephosphorylation (GO:0006470)
Cellular Response To Metal Ion (GO:0071248)
Cellular Respiration (GO:0045333)
Vesicle Coating (GO:0006901)
Oxidative Phosphorylation (GO:0006119)
Phosphatidylinositol Metabolic Process (GO:0046488)
Regulation Of Phospholipid Metabolic Process (GO:1903725)
Aerobic Electron Transport Chain (GO:0019646)
Vesicle Budding From Membrane (GO:0006900)
Mitochondrial ATP Synthesis Coupled Electron Transport (GO:0...
Regulation Of Cell Fate Commitment (GO:0010453)
Regulation Of Cell Fate Specification (GO:0042659)
Dephosphorylation (GO:0016311)
Enriched terms
Gene count
P value
5.0e-06
1.0e-05
1.5e-05
2.0e-05
G
GO Enrichment TLE SOZ vs NIZ DN
Humoral Immune Response Mediated By Circulating Immunoglobul...
Cell Junction Disassembly (GO:0150146)
Synapse Pruning (GO:0098883)
Negative Regulation Of Heterotypic Cell-Cell Adhesion (GO:00...
Positive Regulation Of Monocyte Differentiation (GO:0045657)
Regulation Of Cell-Cell Adhesion Involved In Gastrulation (G...
Regulation Of Macrophage Differentiation (GO:0045649)
Regulation Of Monocyte Differentiation (GO:0045655)
Positive Regulation Of Cell-Substrate Adhesion (GO:0010811)
Regulation Of Heterotypic Cell-Cell Adhesion (GO:0034114)
Osteoclast Differentiation (GO:0030316)
Cytoplasmic Translation (GO:0002181)
Cell-Substrate Junction Assembly (GO:0007044)
Endodermal Cell Differentiation (GO:0035987)
Platelet Aggregation (GO:0070527)
Endoderm Formation (GO:0001706)
tRNA Methylation (GO:0030488)
Positive Regulation Of GTPase Activity (GO:0043547)
Positive Regulation Of Myeloid Leukocyte Differentiation (GO...
Negative Regulation Of Cell-Cell Adhesion (GO:0022408)
Enriched terms
Gene count
P value
0.002
0.004
0.006

Figure S9

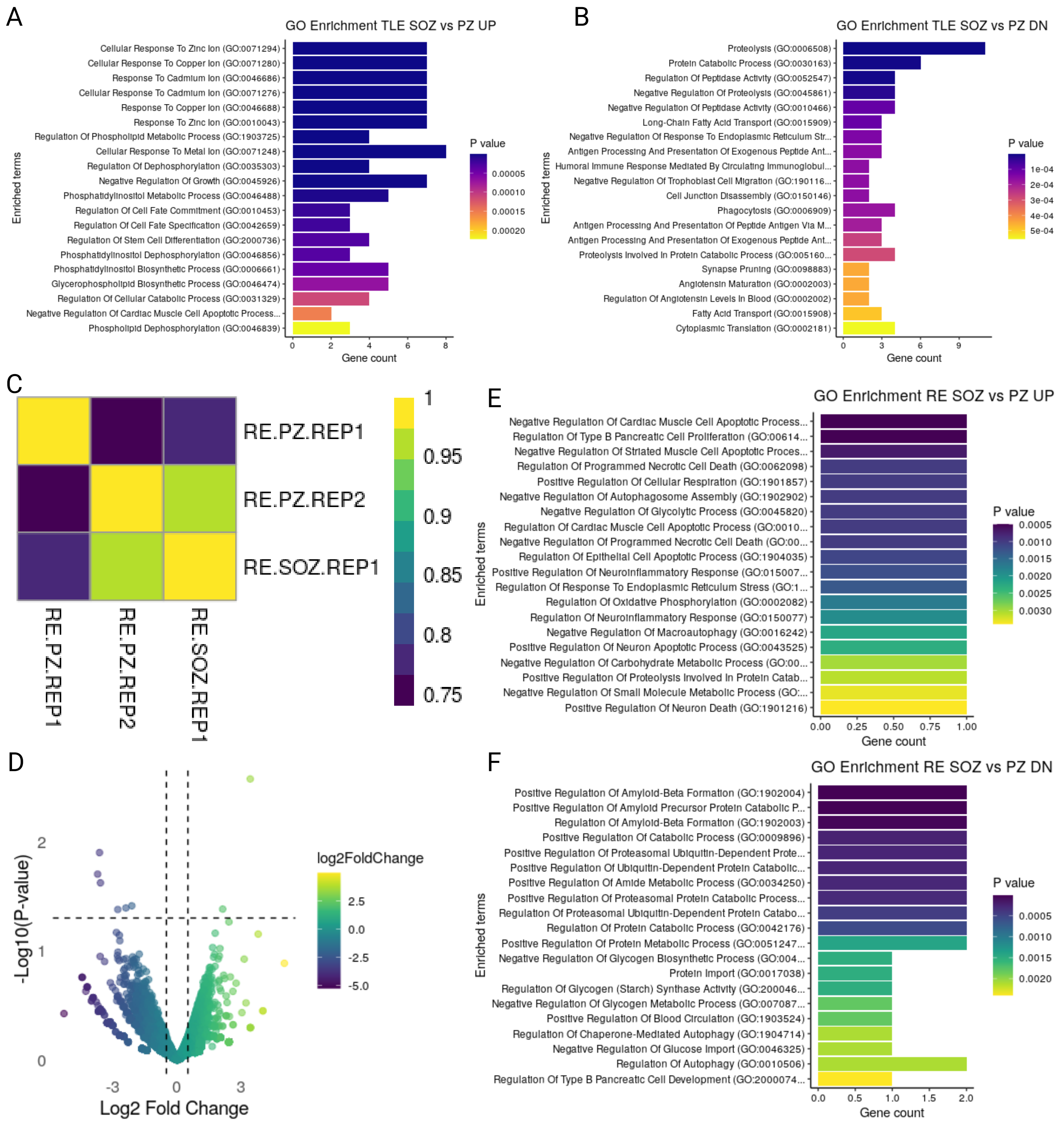
A
GO Enrichment TLE SOZ vs PZ UP
Cellular Response To Zinc Ion (GO:0071294)
Cellular Response To Copper Ion (GO:0071280)
Response To Cadmium Ion (GO:0046686)
Cellular Response To Cadmium Ion (GO:0071276)
Response To Copper Ion (GO:0046688)
Response To Zinc Ion (GO:0010043)
Regulation Of Phospholipid Metabolic Process (GO:1903725)
Cellular Response To Metal Ion (GO:0071248)
Regulation Of Dephosphorylation (GO:0035303)
Negative Regulation Of Growth (GO:0045926)
Phosphatidylinositol Metabolic Process (GO:0046488)
Regulation Of Cell Fate Commitment (GO:0010453)
Regulation Of Cell Fate Specification (GO:0042659)
Regulation Of Stem Cell Differentiation (GO:2000736)
Phosphatidylinositol Dephosphorylation (GO:0046856)
Phosphatidylinositol Biosynthetic Process (GO:0006661)
Glycerophospholipid Biosynthetic Process (GO:0046474)
Regulation Of Cellular Catabolic Process (GO:0031329)
Negative Regulation Of Cardiac Muscle Cell Apoptotic Process...
Phospholipid Dephosphorylation (GO:0046839)
Enriched terms
Gene count
P value
B
GO Enrichment TLE SOZ vs PZ DN
Proteolysis (GO:0006508)
Protein Catabolic Process (GO:0030163)
Regulation Of Peptidase Activity (GO:0052547)
Negative Regulation Of Proteolysis (GO:0045861)
Negative Regulation Of Peptidase Activity (GO:0010466)
Long-Chain Fatty Acid Transport (GO:0015909)
Negative Regulation Of Response To Endoplasmic Reticulum Str...
Antigen Processing And Presentation Of Exogenous Peptide Ant...
Humoral Immune Response Mediated By Circulating Immunoglobul...
Negative Regulation Of Trophoblast Cell Migration (GO:190116...
Cell Junction Disassembly (GO:0150146)
Phagocytosis (GO:0006909)
Antigen Processing And Presentation Of Peptide Antigen Via M...
Antigen Processing And Presentation Of Exogenous Peptide Ant...
Proteolysis Involved In Protein Catabolic Process (GO:005160...
Synapse Pruning (GO:0098883)
Angiotensin Maturation (GO:0002003)
Regulation Of Angiotensin Levels In Blood (GO:0002002)
Fatty Acid Transport (GO:0015908)
Cytoplasmic Translation (GO:0002181)
Enriched terms
Gene count
P value
C
RE.PZ.REP1
RE.PZ.REP2
RE.SOZ.REP1
D
-Log10(P-value)
Log2 Fold Change
log2FoldChange
E
GO Enrichment RE SOZ vs PZ UP
Negative Regulation Of Cardiac Muscle Cell Apoptotic Process...
Regulation Of Type B Pancreatic Cell Proliferation (GO:00614...
Negative Regulation Of Striated Muscle Cell Apoptotic Proces...
Regulation Of Programmed Necrotic Cell Death (GO:0062098)
Positive Regulation Of Cellular Respiration (GO:1901857)
Negative Regulation Of Autophagosome Assembly (GO:1902902)
Negative Regulation Of Glycolytic Process (GO:0045820)
Regulation Of Cardiac Muscle Cell Apoptotic Process (GO:0010...
Negative Regulation Of Programmed Necrotic Cell Death (GO:00...
Regulation Of Epithelial Cell Apoptotic Process (GO:1904035)
Positive Regulation Of Neuroinflammatory Response (GO:015007...
Regulation Of Response To Endoplasmic Reticulum Stress (GO:1...
Regulation Of Oxidative Phosphorylation (GO:0002082)
Regulation Of Neuroinflammatory Response (GO:0150077)
Negative Regulation Of Macroautophagy (GO:0016242)
Positive Regulation Of Neuron Apoptotic Process (GO:0043525)
Negative Regulation Of Carbohydrate Metabolic Process (GO:00...
Positive Regulation Of Proteolysis Involved In Protein Catab...
Negative Regulation Of Small Molecule Metabolic Process (GO:...
Positive Regulation Of Neuron Death (GO:1901216)
Enriched terms
Gene count
P value
F
GO Enrichment RE SOZ vs PZ DN
Positive Regulation Of Amyloid-Beta Formation (GO:1902004)
Positive Regulation Of Amyloid Precursor Protein Catabolic P...
Regulation Of Amyloid-Beta Formation (GO:1902003)
Positive Regulation Of Catabolic Process (GO:0009896)
Positive Regulation Of Proteasomal Ubiquitin-Dependent Prote...
Positive Regulation Of Ubiquitin-Dependent Protein Catabolic...
Positive Regulation Of Amide Metabolic Process (GO:0034250)
Positive Regulation Of Proteasomal Protein Catabolic Process...
Regulation Of Proteasomal Ubiquitin-Dependent Protein Catabo...
Regulation Of Protein Catabolic Process (GO:0042176)
Positive Regulation Of Protein Metabolic Process (GO:0051247...
Negative Regulation Of Glycogen Biosynthetic Process (GO:004...
Protein Import (GO:0017038)
Regulation Of Glycogen (Starch) Synthase Activity (GO:200046...
Negative Regulation Of Glycogen Metabolic Process (GO:007087...
Positive Regulation Of Blood Circulation (GO:1903524)
Regulation Of Chaperone-Mediated Autophagy (GO:1904714)
Negative Regulation Of Glucose Import (GO:0046325)
Regulation Of Autophagy (GO:0010506)
Regulation Of Type B Pancreatic Cell Development (GO:2000074...
Enriched terms
Gene count
P value

Figure S10

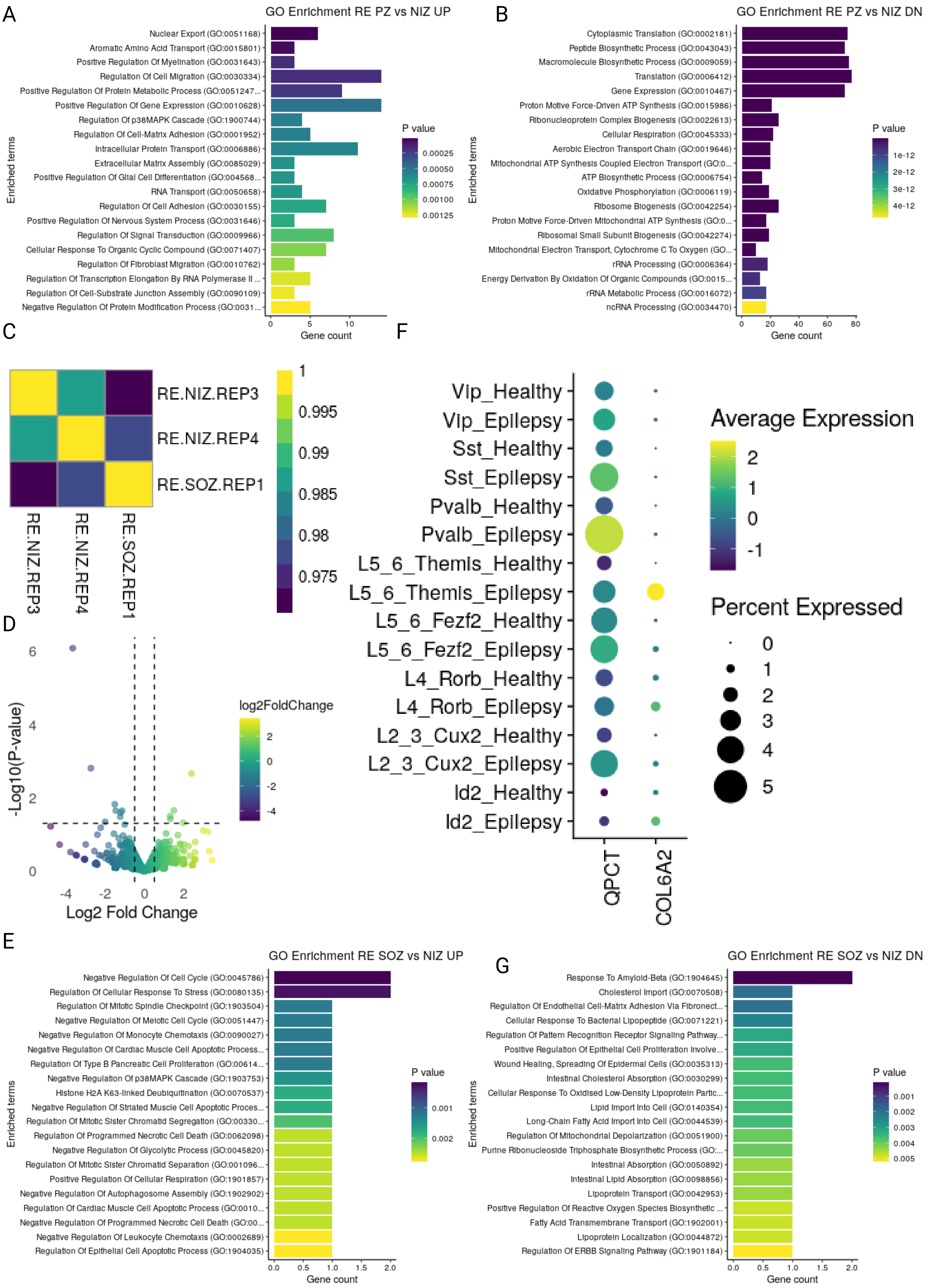

A
GO Enrichment RE PZ vs NIZ UP
Nuclear Export (GO:0051168)
Aromatic Amino Acid Transport (GO:0015801)
Positive Regulation Of Myelination (GO:0031643)
Regulation Of Cell Migration (GO:0030334)
Positive Regulation Of Protein Metabolic Process (GO:0051247...
Positive Regulation Of Gene Expression (GO:0010628)
Regulation Of p38MAPK Cascade (GO:1900744)
Regulation Of Cell-Matrix Adhesion (GO:0001952)
Intracellular Protein Transport (GO:0006886)
Extracellular Matrix Assembly (GO:0085029)
Positive Regulation Of Glial Cell Differentiation (GO:004568...
RNA Transport (GO:0050658)
Regulation Of Cell Adhesion (GO:0030155)
Positive Regulation Of Nervous System Process (GO:0031646)
Regulation Of Signal Transduction (GO:0009966)
Cellular Response To Organic Cyclic Compound (GO:0071407)
Regulation Of Fibroblast Migration (GO:0010762)
Regulation Of Transcription Elongation By RNA Polymerase II ...
Regulation Of Cell-Substrate Junction Assembly (GO:0090109)
Negative Regulation Of Protein Modification Process (GO:0031...
Enriched terms
Gene count
0
5
10
P value
0.00025
0.00050
0.00075
0.00100
0.00125
B
GO Enrichment RE PZ vs NIZ DN
Cytoplasmic Translation (GO:0002181)
Peptide Biosynthetic Process (GO:0043043)
Macromolecule Biosynthetic Process (GO:0009059)
Translation (GO:0006412)
Gene Expression (GO:0010467)
Proton Motive Force-Driven ATP Synthesis (GO:0015986)
Ribonucleoprotein Complex Biogenesis (GO:0022613)
Cellular Respiration (GO:0045333)
Aerobic Electron Transport Chain (GO:0019646)
Mitochondrial ATP Synthesis Coupled Electron Transport (GO:0...
ATP Biosynthetic Process (GO:0006754)
Oxidative Phosphorylation (GO:0006119)
Ribosome Biogenesis (GO:0042254)
Proton Motive Force-Driven Mitochondrial ATP Synthesis (GO:0...
Ribosomal Small Subunit Biogenesis (GO:0042274)
Mitochondrial Electron Transport, Cytochrome C To Oxygen (GO...
rRNA Processing (GO:0006364)
Energy Derivation By Oxidation Of Organic Compounds (GO:0015...
rRNA Metabolic Process (GO:0016072)
ncRNA Processing (GO:0034470)
Enriched terms
Gene count
0
20
40
60
80
P value
1e-12
2e-12
3e-12
4e-12
C
RE.NIZ.REP3
RE.NIZ.REP4
RE.SOZ.REP1
1
0.995
0.99
0.985
0.98
0.975
D
-Log10(P-value)
Log2 Fold Change
log2FoldChange
F
Vip_Healthy
Vip_Epilepsy
Sst_Healthy
Sst_Epilepsy
Pvalb_Healthy
Pvalb_Epilepsy
L5_6_Themis_Healthy
L5_6_Themis_Epilepsy
L5_6_Fezf2_Healthy
L5_6_Fezf2_Epilepsy
L4_Rorb_Healthy
L4_Rorb_Epilepsy
L2_3_Cux2_Healthy
L2_3_Cux2_Epilepsy
Id2_Healthy
Id2_Epilepsy
QPCT
COL6A2
Average Expression
Percent Expressed
E
GO Enrichment RE SOZ vs NIZ UP
Negative Regulation Of Cell Cycle (GO:0045786)
Regulation Of Cellular Response To Stress (GO:0080135)
Regulation Of Mitotic Spindle Checkpoint (GO:1903504)
Negative Regulation Of Meiotic Cell Cycle (GO:0051447)
Negative Regulation Of Monocyte Chemotaxis (GO:0090027)
Negative Regulation Of Cardiac Muscle Cell Apoptotic Process...
Regulation Of Type B Pancreatic Cell Proliferation (GO:00614...
Negative Regulation Of p38MAPK Cascade (GO:1903753)
Histone H2A K63-linked Deubiquitination (GO:0070537)
Negative Regulation Of Striated Muscle Cell Apoptotic Proces...
Regulation Of Mitotic Sister Chromatid Segregation (GO:00330...
Regulation Of Programmed Necrotic Cell Death (GO:0062098)
Negative Regulation Of Glycolytic Process (GO:0045820)
Regulation Of Mitotic Sister Chromatid Separation (GO:001096...
Positive Regulation Of Cellular Respiration (GO:1901857)
Negative Regulation Of Autophagosome Assembly (GO:1902902)
Regulation Of Cardiac Muscle Cell Apoptotic Process (GO:0010...
Negative Regulation Of Programmed Necrotic Cell Death (GO:00...
Negative Regulation Of Leukocyte Chemotaxis (GO:0002689)
Regulation Of Epithelial Cell Apoptotic Process (GO:1904035)
Enriched terms
Gene count
0.0
0.5
1.0
1.5
2.0
P value
0.001
0.002
G
GO Enrichment RE SOZ vs NIZ DN
Response To Amyloid-Beta (GO:1904645)
Cholesterol Import (GO:0070508)
Regulation Of Endothelial Cell-Matrix Adhesion Via Fibronect...
Cellular Response To Bacterial Lipopeptide (GO:0071221)
Regulation Of Pattern Recognition Receptor Signaling Pathway...
Positive Regulation Of Epithelial Cell Proliferation Involve...
Wound Healing, Spreading Of Epidermal Cells (GO:0035313)
Intestinal Cholesterol Absorption (GO:0030299)
Cellular Response To Oxidised Low-Density Lipoprotein Partic...
Lipid Import Into Cell (GO:0140354)
Long-Chain Fatty Acid Import Into Cell (GO:0044539)
Regulation Of Mitochondrial Depolarization (GO:0051900)
Purine Ribonucleoside Triphosphate Biosynthetic Process (GO:...
Intestinal Absorption (GO:0050892)
Intestinal Lipid Absorption (GO:0098856)
Lipoprotein Transport (GO:0042953)
Positive Regulation Of Reactive Oxygen Species Biosynthetic ...
Fatty Acid Transmembrane Transport (GO:1902001)
Lipoprotein Localization (GO:0044872)
Regulation Of ERBB Signaling Pathway (GO:1901184)
Enriched terms
Gene count
0.0
0.5
1.0
1.5
2.0
P value
0.001
0.002
0.003
0.004
0.005

Figure S11

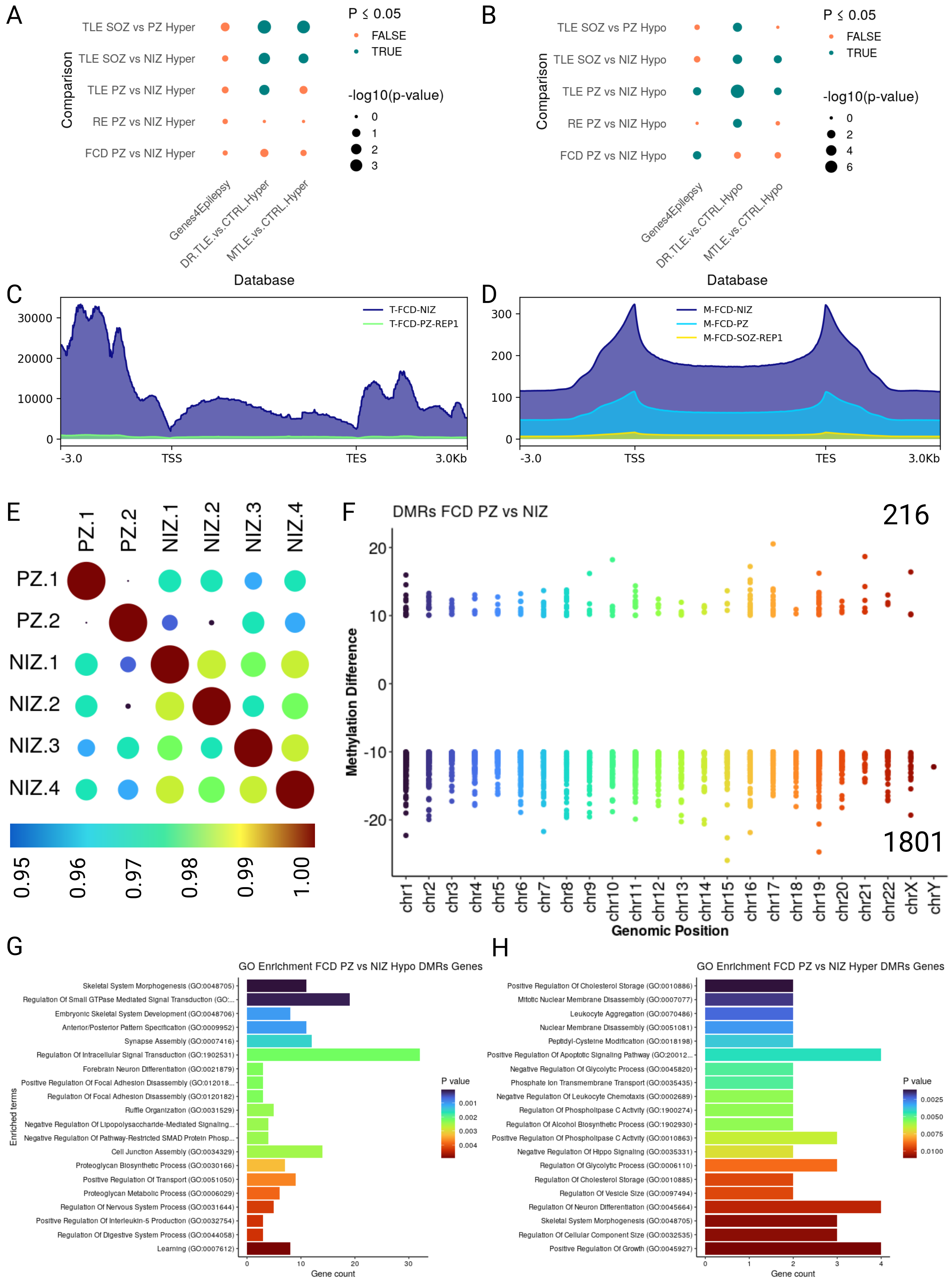
A
TLE SOZ vs PZ Hyper
TLE SOZ vs NIZ Hyper
TLE PZ vs NIZ Hyper
RE PZ vs NIZ Hyper
FCD PZ vs NIZ Hyper
Comparison
Genes4Epilepsy
DR.TLE.vs.CTRL.Hyper
MTLE.vs.CTRL.Hyper
Database
P ≤ 0.05
FALSE
TRUE
-log10(p-value)
B
TLE SOZ vs PZ Hypo
TLE SOZ vs NIZ Hypo
TLE PZ vs NIZ Hypo
RE PZ vs NIZ Hypo
FCD PZ vs NIZ Hypo
DR.TLE.vs.CTRL.Hypo
MTLE.vs.CTRL.Hypo
C
T-FCD-NIZ
T-FCD-PZ-REP1
TSS
TES
3.0Kb
D
M-FCD-NIZ
M-FCD-PZ
M-FCD-SOZ-REP1
E
PZ.1
PZ.2
NIZ.1
NIZ.2
NIZ.3
NIZ.4
F
DMRs FCD PZ vs NIZ
216
1801
Methylation Difference
Genomic Position
G
GO Enrichment FCD PZ vs NIZ Hypo DMRs Genes
Skeletal System Morphogenesis (GO:0048705)
Regulation Of Small GTPase Mediated Signal Transduction (GO:...
Embryonic Skeletal System Development (GO:0048706)
Anterior/Posterior Pattern Specification (GO:0009952)
Synapse Assembly (GO:0007416)
Regulation Of Intracellular Signal Transduction (GO:1902531)
Forebrain Neuron Differentiation (GO:0021879)
Positive Regulation Of Focal Adhesion Disassembly (GO:012018...
Regulation Of Focal Adhesion Disassembly (GO:0120182)
Ruffle Organization (GO:0031529)
Negative Regulation Of Lipopolysaccharide-Mediated Signaling...
Negative Regulation Of Pathway-Restricted SMAD Protein Phosp...
Cell Junction Assembly (GO:0034329)
Proteoglycan Biosynthetic Process (GO:0030166)
Positive Regulation Of Transport (GO:0051050)
Proteoglycan Metabolic Process (GO:0006029)
Regulation Of Nervous System Process (GO:0031644)
Positive Regulation Of Interleukin-5 Production (GO:0032754)
Regulation Of Digestive System Process (GO:0044058)
Learning (GO:0007612)
Enriched terms
Gene count
P value
H
GO Enrichment FCD PZ vs NIZ Hyper DMRs Genes
Positive Regulation Of Cholesterol Storage (GO:0010886)
Mitotic Nuclear Membrane Disassembly (GO:0007077)
Leukocyte Aggregation (GO:0070486)
Nuclear Membrane Disassembly (GO:0051081)
Peptidyl-Cysteine Modification (GO:0018198)
Positive Regulation Of Apoptotic Signaling Pathway (GO:20012...
Negative Regulation Of Glycolytic Process (GO:0045820)
Phosphate Ion Transmembrane Transport (GO:0035435)
Negative Regulation Of Leukocyte Chemotaxis (GO:0002689)
Regulation Of Phospholipase C Activity (GO:1900274)
Regulation Of Alcohol Biosynthetic Process (GO:1902930)
Positive Regulation Of Phospholipase C Activity (GO:0010863)
Negative Regulation Of Hippo Signaling (GO:0035331)
Regulation Of Glycolytic Process (GO:0006110)
Regulation Of Cholesterol Storage (GO:0010885)
Regulation Of Vesicle Size (GO:0097494)
Regulation Of Neuron Differentiation (GO:0045664)
Skeletal System Morphogenesis (GO:0048705)
Regulation Of Cellular Component Size (GO:0032535)
Positive Regulation Of Growth (GO:0045927)
Gene count
P value

Figure S12

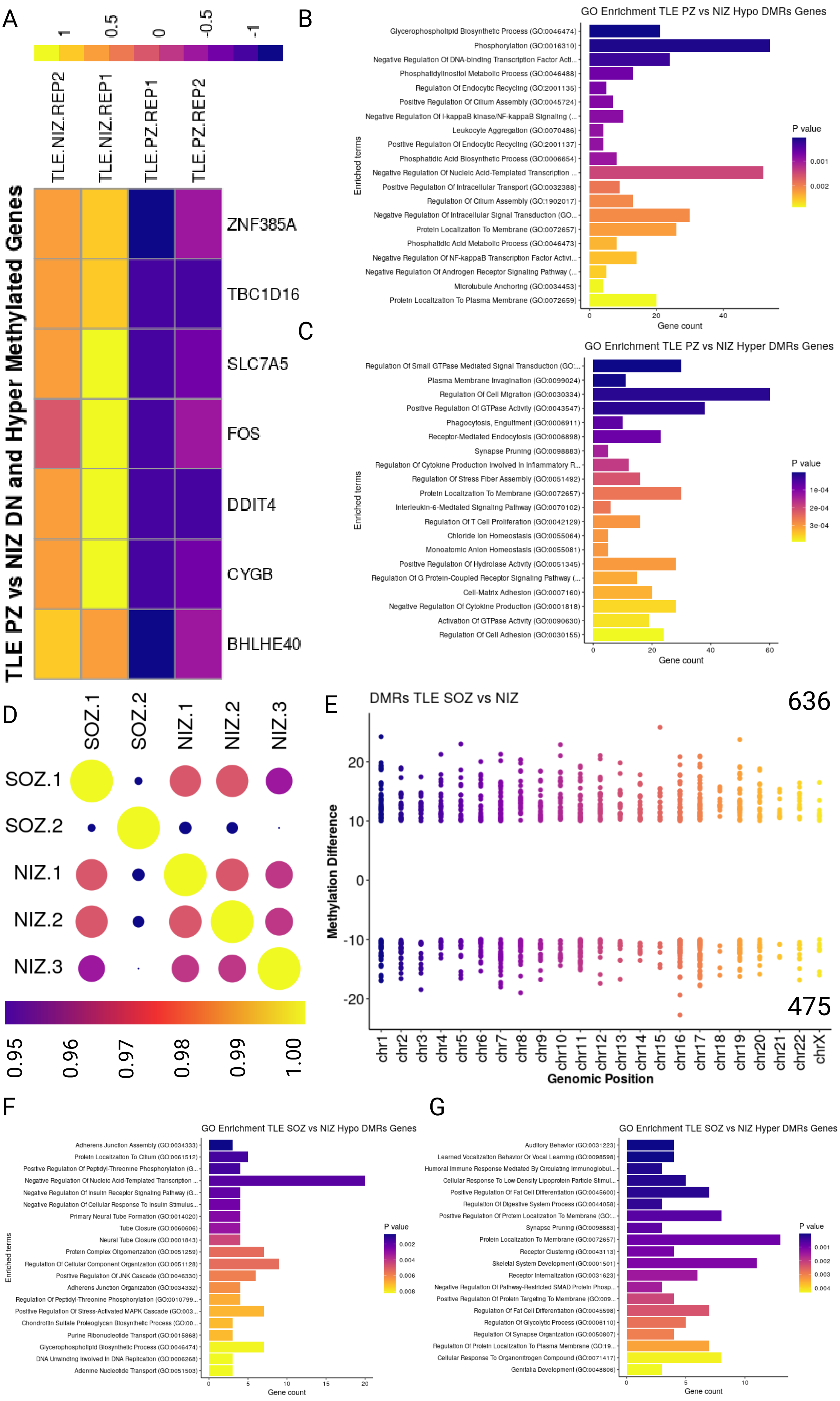

A
1
0.5
0
-0.5
-1
TLE.NIZ.REP2
TLE.NIZ.REP1
TLE.PZ.REP1
TLE.PZ.REP2
TLE PZ vs NIZ DN and Hyper Methylated Genes
ZNF385A
TBC1D16
SLC7A5
FOS
DDIT4
CYGB
BHLHE40
B
GO Enrichment TLE PZ vs NIZ Hypo DMRs Genes
Glycerophospholipid Biosynthetic Process (GO:0046474)
Phosphorylation (GO:0016310)
Negative Regulation Of DNA-binding Transcription Factor Acti...
Phosphatidylinositol Metabolic Process (GO:0046488)
Regulation Of Endocytic Recycling (GO:2001135)
Positive Regulation Of Cilium Assembly (GO:0045724)
Negative Regulation Of I-kappaB kinase/NF-kappaB Signaling (...
Leukocyte Aggregation (GO:0070486)
Positive Regulation Of Endocytic Recycling (GO:2001137)
Phosphatidic Acid Biosynthetic Process (GO:0006654)
Negative Regulation Of Nucleic Acid-Templated Transcription ...
Positive Regulation Of Intracellular Transport (GO:0032388)
Regulation Of Cilium Assembly (GO:1902017)
Negative Regulation Of Intracellular Signal Transduction (GO...
Protein Localization To Membrane (GO:0072657)
Phosphatidic Acid Metabolic Process (GO:0046473)
Negative Regulation Of NF-kappaB Transcription Factor Activi...
Negative Regulation Of Androgen Receptor Signaling Pathway (...
Microtubule Anchoring (GO:0034453)
Protein Localization To Plasma Membrane (GO:0072659)
Enriched terms
Gene count
0
20
40
P value
0.001
0.002
C
GO Enrichment TLE PZ vs NIZ Hyper DMRs Genes
Regulation Of Small GTPase Mediated Signal Transduction (GO:...
Plasma Membrane Invagination (GO:0099024)
Regulation Of Cell Migration (GO:0030334)
Positive Regulation Of GTPase Activity (GO:0043547)
Phagocytosis, Engulfment (GO:0006911)
Receptor-Mediated Endocytosis (GO:0006898)
Synapse Pruning (GO:0098883)
Regulation Of Cytokine Production Involved In Inflammatory R...
Regulation Of Stress Fiber Assembly (GO:0051492)
Protein Localization To Membrane (GO:0072657)
Interleukin-6-Mediated Signaling Pathway (GO:0070102)
Regulation Of T Cell Proliferation (GO:0042129)
Chloride Ion Homeostasis (GO:0055064)
Monoatomic Anion Homeostasis (GO:0055081)
Positive Regulation Of Hydrolase Activity (GO:0051345)
Regulation Of G Protein-Coupled Receptor Signaling Pathway (...
Cell-Matrix Adhesion (GO:0007160)
Negative Regulation Of Cytokine Production (GO:0001818)
Activation Of GTPase Activity (GO:0090630)
Regulation Of Cell Adhesion (GO:0030155)
Enriched terms
Gene count
0
20
40
60
P value
1e-04
2e-04
3e-04
D
SOZ.1
SOZ.2
NIZ.1
NIZ.2
NIZ.3
0.95
0.96
0.97
0.98
0.99
1.00
E
DMRs TLE SOZ vs NIZ
636
475
Methylation Difference
20
10
0
-10
-20
chr1
chr2
chr3
chr4
chr5
chr6
chr7
chr8
chr9
chr10
chr11
chr12
chr13
chr14
chr15
chr16
chr17
chr18
chr19
chr20
chr21
chr22
chrX
Genomic Position
F
GO Enrichment TLE SOZ vs NIZ Hypo DMRs Genes
Adherens Junction Assembly (GO:0034333)
Protein Localization To Cilium (GO:0061512)
Positive Regulation Of Peptidyl-Threonine Phosphorylation (G...
Negative Regulation Of Nucleic Acid-Templated Transcription ...
Negative Regulation Of Insulin Receptor Signaling Pathway (G...
Negative Regulation Of Cellular Response To Insulin Stimulus...
Primary Neural Tube Formation (GO:0014020)
Tube Closure (GO:0060606)
Neural Tube Closure (GO:0001843)
Protein Complex Oligomerization (GO:0051259)
Regulation Of Cellular Component Organization (GO:0051128)
Positive Regulation Of JNK Cascade (GO:0046330)
Adherens Junction Organization (GO:0034332)
Regulation Of Peptidyl-Threonine Phosphorylation (GO:0010799...
Positive Regulation Of Stress-Activated MAPK Cascade (GO:003...
Chondroitin Sulfate Proteoglycan Biosynthetic Process (GO:00...
Purine Ribonucleotide Transport (GO:0015868)
Glycerophospholipid Biosynthetic Process (GO:0046474)
DNA Unwinding Involved In DNA Replication (GO:0006268)
Adenine Nucleotide Transport (GO:0051503)
Enriched terms
Gene count
0
5
10
15
20
P value
0.002
0.004
0.006
0.008
G
GO Enrichment TLE SOZ vs NIZ Hyper DMRs Genes
Auditory Behavior (GO:0031223)
Learned Vocalization Behavior Or Vocal Learning (GO:0098598)
Humoral Immune Response Mediated By Circulating Immunoglobul...
Cellular Response To Low-Density Lipoprotein Particle Stimul...
Positive Regulation Of Fat Cell Differentiation (GO:0045600)
Regulation Of Digestive System Process (GO:0044058)
Positive Regulation Of Protein Localization To Membrane (GO:...
Synapse Pruning (GO:0098883)
Protein Localization To Membrane (GO:0072657)
Receptor Clustering (GO:0043113)
Skeletal System Development (GO:0001501)
Receptor Internalization (GO:0031623)
Negative Regulation Of Pathway-Restricted SMAD Protein Phosp...
Positive Regulation Of Protein Targeting To Membrane (GO:009...
Regulation Of Fat Cell Differentiation (GO:0045598)
Regulation Of Glycolytic Process (GO:0006110)
Regulation Of Synapse Organization (GO:0050807)
Regulation Of Protein Localization To Plasma Membrane (GO:19...
Cellular Response To Organonitrogen Compound (GO:0071417)
Genitalia Development (GO:0048806)
Gene count
0
5
10
P value
0.001
0.002
0.003
0.004

Figure S13

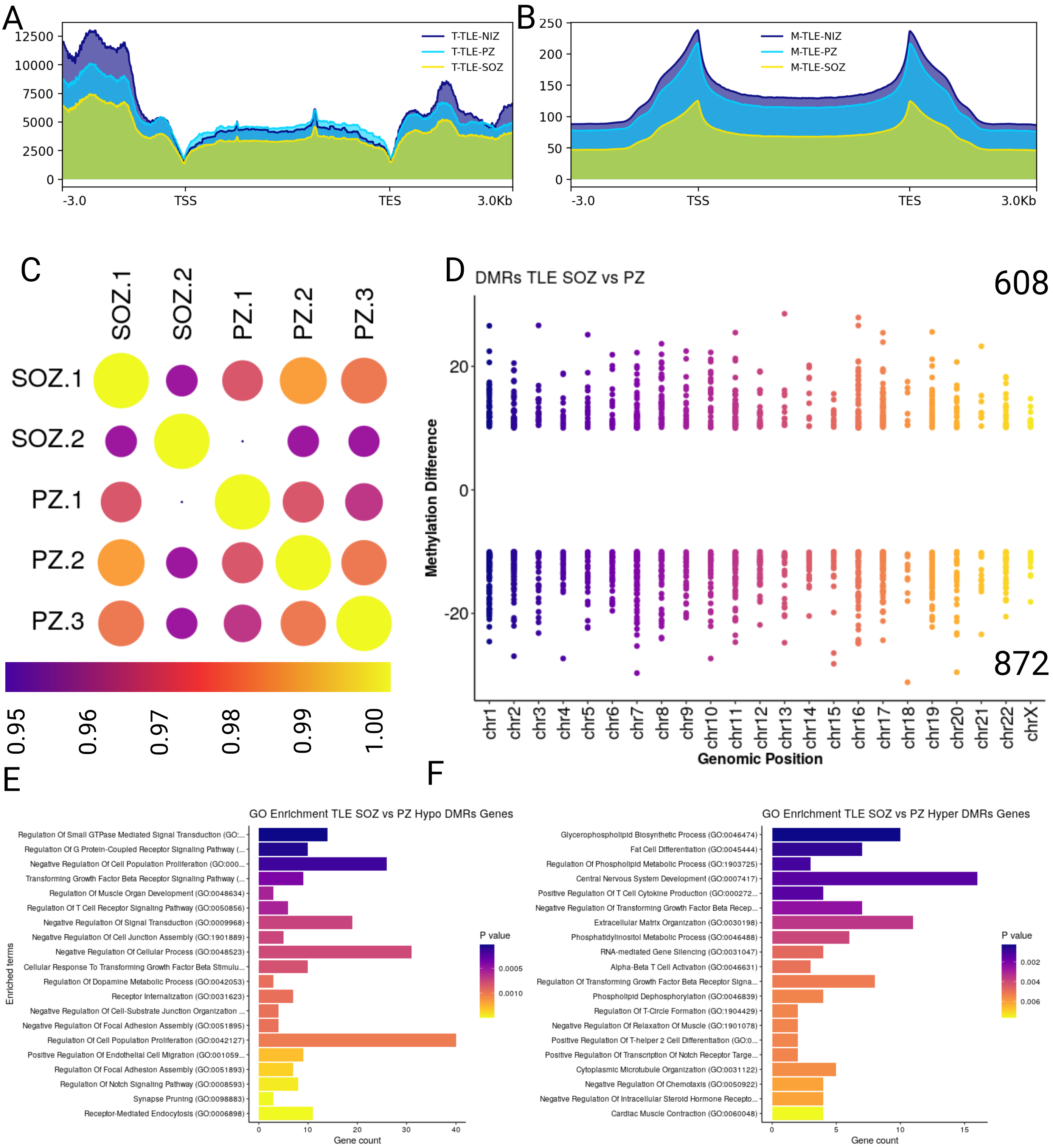
A
T-TLE-NIZ
T-TLE-PZ
T-TLE-SOZ
12500
10000
7500
5000
2500
0
-3.0
TSS
TES
3.0Kb
B
M-TLE-NIZ
M-TLE-PZ
M-TLE-SOZ
250
200
150
100
50
0
-3.0
TSS
TES
3.0Kb
C
SOZ.1
SOZ.2
PZ.1
PZ.2
PZ.3
0.95
0.96
0.97
0.98
0.99
1.00
D
DMRs TLE SOZ vs PZ
608
872
Methylation Difference
20
0
-20
chr1 chr2 chr3 chr4 chr5 chr6 chr7 chr8 chr9 chr10 chr11 chr12 chr13 chr14 chr15 chr16 chr17 chr18 chr19 chr20 chr21 chr22 chrX
Genomic Position
E
GO Enrichment TLE SOZ vs PZ Hypo DMRs Genes
Enriched terms
Regulation Of Small GTPase Mediated Signal Transduction (GO:...
Regulation Of G Protein-Coupled Receptor Signaling Pathway (...
Negative Regulation Of Cell Population Proliferation (GO:000...
Transforming Growth Factor Beta Receptor Signaling Pathway (...
Regulation Of Muscle Organ Development (GO:0048634)
Regulation Of T Cell Receptor Signaling Pathway (GO:0050856)
Negative Regulation Of Signal Transduction (GO:0009968)
Negative Regulation Of Cell Junction Assembly (GO:1901889)
Negative Regulation Of Cellular Process (GO:0048523)
Cellular Response To Transforming Growth Factor Beta Stimulu...
Regulation Of Dopamine Metabolic Process (GO:0042053)
Receptor Internalization (GO:0031623)
Negative Regulation Of Cell-Substrate Junction Organization ...
Negative Regulation Of Focal Adhesion Assembly (GO:0051895)
Regulation Of Cell Population Proliferation (GO:0042127)
Positive Regulation Of Endothelial Cell Migration (GO:001059...
Regulation Of Focal Adhesion Assembly (GO:0051893)
Regulation Of Notch Signaling Pathway (GO:0008593)
Synapse Pruning (GO:0098883)
Receptor-Mediated Endocytosis (GO:0006898)
0
10
20
30
40
Gene count
P value
0.0005
0.0010
F
GO Enrichment TLE SOZ vs PZ Hyper DMRs Genes
Glycerophospholipid Biosynthetic Process (GO:0046474)
Fat Cell Differentiation (GO:0045444)
Regulation Of Phospholipid Metabolic Process (GO:1903725)
Central Nervous System Development (GO:0007417)
Positive Regulation Of T Cell Cytokine Production (GO:000272...
Negative Regulation Of Transforming Growth Factor Beta Recep...
Extracellular Matrix Organization (GO:0030198)
Phosphatidylinositol Metabolic Process (GO:0046488)
RNA-mediated Gene Silencing (GO:0031047)
Alpha-Beta T Cell Activation (GO:0046631)
Regulation Of Transforming Growth Factor Beta Receptor Signa...
Phospholipid Dephosphorylation (GO:0046839)
Regulation Of T-Circle Formation (GO:1904429)
Negative Regulation Of Relaxation Of Muscle (GO:1901078)
Positive Regulation Of T-helper 2 Cell Differentiation (GO:0...
Positive Regulation Of Transcription Of Notch Receptor Targe...
Cytoplasmic Microtubule Organization (GO:0031122)
Negative Regulation Of Chemotaxis (GO:0050922)
Negative Regulation Of Intracellular Steroid Hormone Recepto...
Cardiac Muscle Contraction (GO:0060048)
0
5
10
15
Gene count
P value
0.002
0.004
0.006

Figure S14

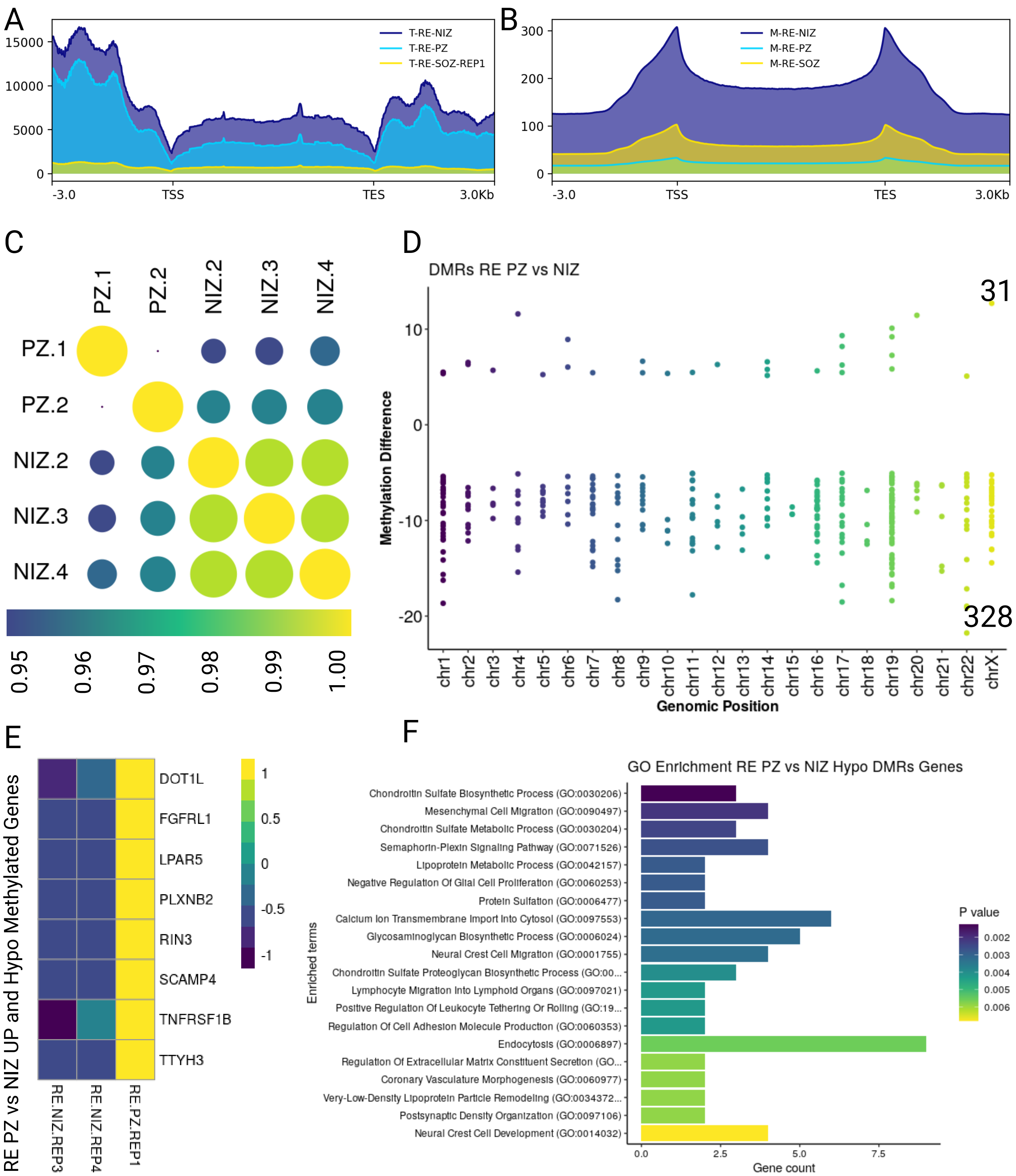
A
T-RE-NIZ
T-RE-PZ
T-RE-SOZ-REP1
15000
10000
5000
0
-3.0
TSS
TES
3.0Kb
B
M-RE-NIZ
M-RE-PZ
M-RE-SOZ
300
200
100
0
-3.0
TSS
TES
3.0Kb
C
PZ.1
PZ.2
NIZ.2
NIZ.3
NIZ.4
0.95
0.96
0.97
0.98
0.99
1.00
D
DMRs RE PZ vs NIZ
31
328
Methylation Difference
10
0
-10
-20
chr1 chr2 chr3 chr4 chr5 chr6 chr7 chr8 chr9 chr10 chr11 chr12 chr13 chr14 chr15 chr16 chr17 chr18 chr19 chr20 chr21 chr22 chrX
Genomic Position
E
RE PZ vs NIZ UP and Hypo Methylated Genes
DOT1L
FGFRL1
LPAR5
PLXNB2
RIN3
SCAMP4
TNFRSF1B
TTYH3
RE.NIZ.REP3
RE.NIZ.REP4
RE.PZ.REP1
1
0.5
0
-0.5
-1
F
GO Enrichment RE PZ vs NIZ Hypo DMRs Genes
Enriched terms
Chondroitin Sulfate Biosynthetic Process (GO:0030206)
Mesenchymal Cell Migration (GO:0090497)
Chondroitin Sulfate Metabolic Process (GO:0030204)
Semaphorin-Plexin Signaling Pathway (GO:0071526)
Lipoprotein Metabolic Process (GO:0042157)
Negative Regulation Of Glial Cell Proliferation (GO:0060253)
Protein Sulfation (GO:0006477)
Calcium Ion Transmembrane Import Into Cytosol (GO:0097553)
Glycosaminoglycan Biosynthetic Process (GO:0006024)
Neural Crest Cell Migration (GO:0001755)
Chondroitin Sulfate Proteoglycan Biosynthetic Process (GO:00...
Lymphocyte Migration Into Lymphoid Organs (GO:0097021)
Positive Regulation Of Leukocyte Tethering Or Rolling (GO:19...
Regulation Of Cell Adhesion Molecule Production (GO:0060353)
Endocytosis (GO:0006897)
Regulation Of Extracellular Matrix Constituent Secretion (GO...
Coronary Vasculature Morphogenesis (GO:0060977)
Very-Low-Density Lipoprotein Particle Remodeling (GO:0034372...
Postsynaptic Density Organization (GO:0097106)
Neural Crest Cell Development (GO:0014032)
0.0
2.5
5.0
7.5
Gene count
P value
0.002
0.003
0.004
0.005
0.006

Figure S15

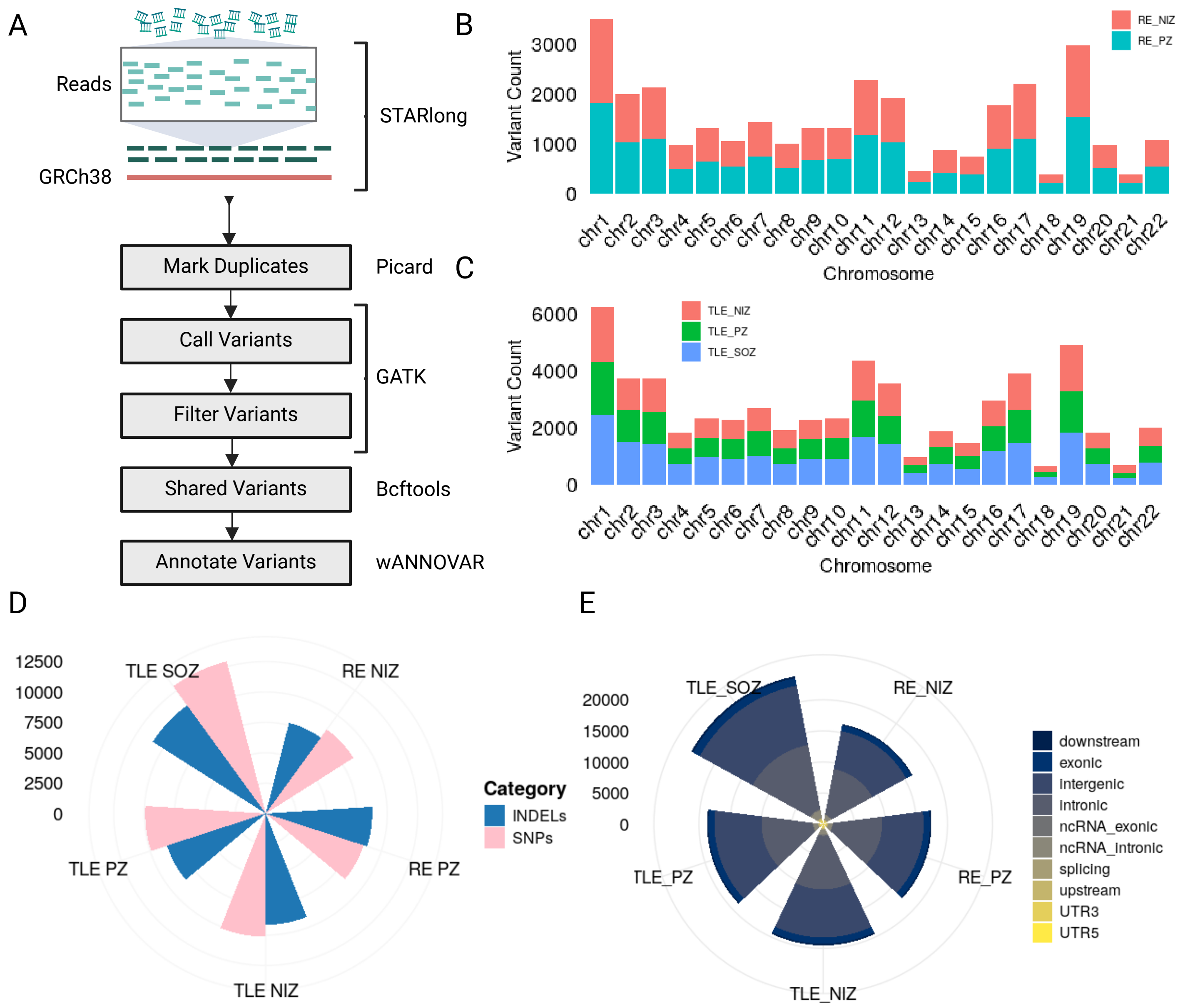
A
Reads
GRCh38
STARlong
Mark Duplicates
Picard
Call Variants
Filter Variants
GATK
Shared Variants
Bcftools
Annotate Variants
wANNOVAR
B
Variant Count
3000
2000
1000
0
RE_NIZ
RE_PZ
chr1 chr2 chr3 chr4 chr5 chr6 chr7 chr8 chr9 chr10 chr11 chr12 chr13 chr14 chr15 chr16 chr17 chr18 chr19 chr20 chr21 chr22
Chromosome
C
Variant Count
6000
4000
2000
0
TLE_NIZ
TLE_PZ
TLE_SOZ
chr1 chr2 chr3 chr4 chr5 chr6 chr7 chr8 chr9 chr10 chr11 chr12 chr13 chr14 chr15 chr16 chr17 chr18 chr19 chr20 chr21 chr22
Chromosome
D
12500
10000
7500
5000
2500
0
TLE SOZ
RE NIZ
RE PZ
TLE NIZ
TLE PZ
Category
INDELs
SNPs
E
20000
15000
10000
5000
0
TLE_SOZ
RE_NIZ
RE_PZ
TLE_NIZ
TLE_PZ
downstream
exonic
intergenic
intronic
ncRNA_exonic
ncRNA_intronic
splicing
upstream
UTR3
UTR5

Figure S16

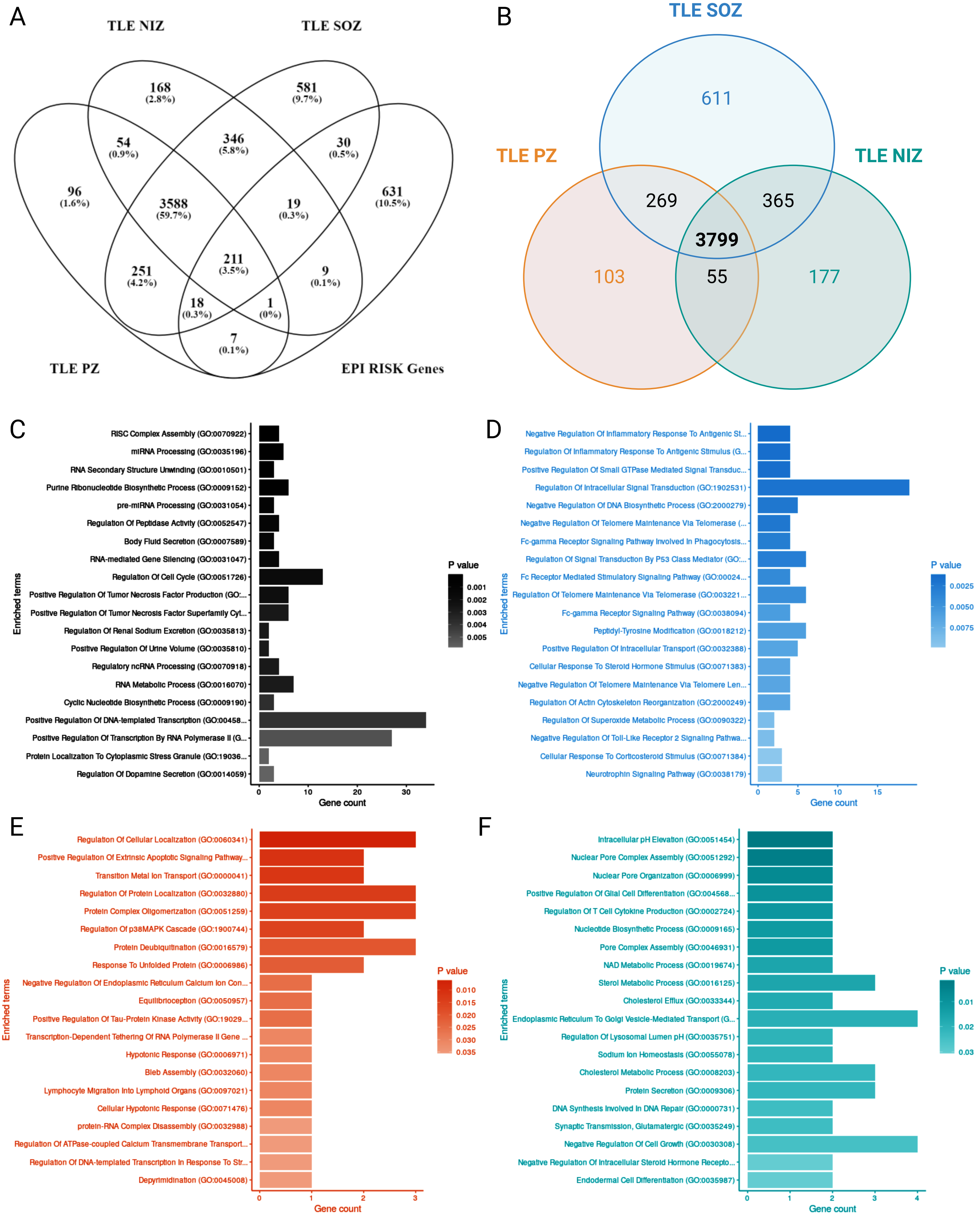
A
TLE NIZ
TLE SOZ
TLE PZ
EPI RISK Genes
168 (2.8%)
581 (9.7%)
54 (0.9%)
346 (5.8%)
30 (0.5%)
96 (1.6%)
3588 (59.7%)
19 (0.3%)
631 (10.5%)
211 (3.5%)
251 (4.2%)
9 (0.1%)
18 (0.3%)
1 (0%)
7 (0.1%)
B
TLE SOZ
TLE PZ
TLE NIZ
611
269
365
3799
103
55
177
C
RISC Complex Assembly (GO:0070922)
miRNA Processing (GO:0035196)
RNA Secondary Structure Unwinding (GO:0010501)
Purine Ribonucleotide Biosynthetic Process (GO:0009152)
pre-miRNA Processing (GO:0031054)
Regulation Of Peptidase Activity (GO:0052547)
Body Fluid Secretion (GO:0007589)
RNA-mediated Gene Silencing (GO:0031047)
Regulation Of Cell Cycle (GO:0051726)
Positive Regulation Of Tumor Necrosis Factor Production (GO:...
Positive Regulation Of Tumor Necrosis Factor Superfamily Cyt...
Regulation Of Renal Sodium Excretion (GO:0035813)
Positive Regulation Of Urine Volume (GO:0035810)
Regulatory ncRNA Processing (GO:0070918)
RNA Metabolic Process (GO:0016070)
Cyclic Nucleotide Biosynthetic Process (GO:0009190)
Positive Regulation Of DNA-templated Transcription (GO:00458...
Positive Regulation Of Transcription By RNA Polymerase II (G...
Protein Localization To Cytoplasmic Stress Granule (GO:19036...
Regulation Of Dopamine Secretion (GO:0014059)
Enriched terms
Gene count
0
10
20
30
P value
0.001
0.002
0.003
0.004
0.005
D
Negative Regulation Of Inflammatory Response To Antigenic St...
Regulation Of Inflammatory Response To Antigenic Stimulus (G...
Positive Regulation Of Small GTPase Mediated Signal Transduc...
Regulation Of Intracellular Signal Transduction (GO:1902531)
Negative Regulation Of DNA Biosynthetic Process (GO:2000279)
Negative Regulation Of Telomere Maintenance Via Telomerase (...
Fc-gamma Receptor Signaling Pathway Involved In Phagocytosis...
Regulation Of Signal Transduction By P53 Class Mediator (GO:...
Fc Receptor Mediated Stimulatory Signaling Pathway (GO:00024...
Regulation Of Telomere Maintenance Via Telomerase (GO:003221...
Fc-gamma Receptor Signaling Pathway (GO:0038094)
Peptidyl-Tyrosine Modification (GO:0018212)
Positive Regulation Of Intracellular Transport (GO:0032388)
Cellular Response To Steroid Hormone Stimulus (GO:0071383)
Negative Regulation Of Telomere Maintenance Via Telomere Len...
Regulation Of Actin Cytoskeleton Reorganization (GO:2000249)
Regulation Of Superoxide Metabolic Process (GO:0090322)
Negative Regulation Of Toll-Like Receptor 2 Signaling Pathwa...
Cellular Response To Corticosteroid Stimulus (GO:0071384)
Neurotrophin Signaling Pathway (GO:0038179)
Enriched terms
Gene count
0
5
10
15
P value
0.0025
0.0050
0.0075
E
Regulation Of Cellular Localization (GO:0060341)
Positive Regulation Of Extrinsic Apoptotic Signaling Pathway...
Transition Metal Ion Transport (GO:0000041)
Regulation Of Protein Localization (GO:0032880)
Protein Complex Oligomerization (GO:0051259)
Regulation Of p38MAPK Cascade (GO:1900744)
Protein Deubiquitination (GO:0016579)
Response To Unfolded Protein (GO:0006986)
Negative Regulation Of Endoplasmic Reticulum Calcium Ion Con...
Equilibrioception (GO:0050957)
Positive Regulation Of Tau-Protein Kinase Activity (GO:19029...
Transcription-Dependent Tethering Of RNA Polymerase II Gene ...
Hypotonic Response (GO:0006971)
Bleb Assembly (GO:0032060)
Lymphocyte Migration Into Lymphoid Organs (GO:0097021)
Cellular Hypotonic Response (GO:0071476)
protein-RNA Complex Disassembly (GO:0032988)
Regulation Of ATPase-coupled Calcium Transmembrane Transport...
Regulation Of DNA-templated Transcription In Response To Str...
Depyrimidination (GO:0045008)
Enriched terms
Gene count
0
1
2
3
P value
0.010
0.015
0.020
0.025
0.030
0.035
F
Intracellular pH Elevation (GO:0051454)
Nuclear Pore Complex Assembly (GO:0051292)
Nuclear Pore Organization (GO:0006999)
Positive Regulation Of Glial Cell Differentiation (GO:004568...
Regulation Of T Cell Cytokine Production (GO:0002724)
Nucleotide Biosynthetic Process (GO:0009165)
Pore Complex Assembly (GO:0046931)
NAD Metabolic Process (GO:0019674)
Sterol Metabolic Process (GO:0016125)
Cholesterol Efflux (GO:0033344)
Endoplasmic Reticulum To Golgi Vesicle-Mediated Transport (G...
Regulation Of Lysosomal Lumen pH (GO:0035751)
Sodium Ion Homeostasis (GO:0055078)
Cholesterol Metabolic Process (GO:0008203)
Protein Secretion (GO:0009306)
DNA Synthesis Involved In DNA Repair (GO:0000731)
Synaptic Transmission, Glutamatergic (GO:0035249)
Negative Regulation Of Cell Growth (GO:0030308)
Negative Regulation Of Intracellular Steroid Hormone Recepto...
Endodermal Cell Differentiation (GO:0035987)
Enriched terms
Gene count
0
1
2
3
4
P value
0.01
0.02
0.03

Figure S17

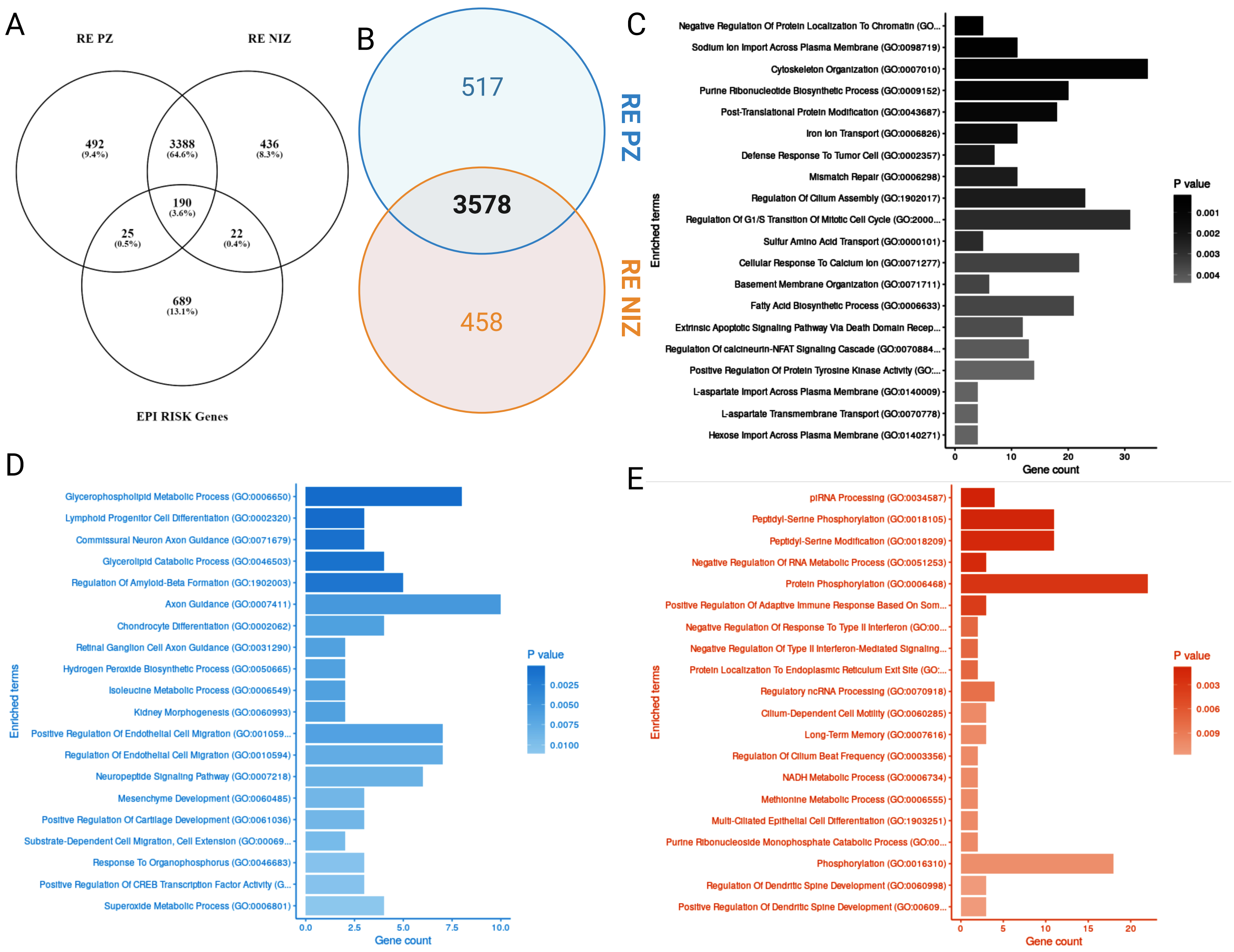
A
RE PZ
RE NIZ
492 (9.4%)
3388 (64.6%)
436 (8.3%)
190 (3.6%)
25 (0.5%)
22 (0.4%)
689 (13.1%)
EPI RISK Genes
B
517
3578
458
RE PZ
RE NIZ
C
Negative Regulation Of Protein Localization To Chromatin (GO...
Sodium Ion Import Across Plasma Membrane (GO:0098719)
Cytoskeleton Organization (GO:0007010)
Purine Ribonucleotide Biosynthetic Process (GO:0009152)
Post-Translational Protein Modification (GO:0043687)
Iron Ion Transport (GO:0006826)
Defense Response To Tumor Cell (GO:0002357)
Mismatch Repair (GO:0006298)
Regulation Of Cilium Assembly (GO:1902017)
Regulation Of G1/S Transition Of Mitotic Cell Cycle (GO:2000...
Sulfur Amino Acid Transport (GO:0000101)
Cellular Response To Calcium Ion (GO:0071277)
Basement Membrane Organization (GO:0071711)
Fatty Acid Biosynthetic Process (GO:0006633)
Extrinsic Apoptotic Signaling Pathway Via Death Domain Recep...
Regulation Of calcineurin-NFAT Signaling Cascade (GO:0070884...
Positive Regulation Of Protein Tyrosine Kinase Activity (GO:...
L-aspartate Import Across Plasma Membrane (GO:0140009)
L-aspartate Transmembrane Transport (GO:0070778)
Hexose Import Across Plasma Membrane (GO:0140271)
Enriched terms
Gene count
0 10 20 30
P value
0.001
0.002
0.003
0.004
D
Glycerophospholipid Metabolic Process (GO:0006650)
Lymphoid Progenitor Cell Differentiation (GO:0002320)
Commissural Neuron Axon Guidance (GO:0071679)
Glycerolipid Catabolic Process (GO:0046503)
Regulation Of Amyloid-Beta Formation (GO:1902003)
Axon Guidance (GO:0007411)
Chondrocyte Differentiation (GO:0002062)
Retinal Ganglion Cell Axon Guidance (GO:0031290)
Hydrogen Peroxide Biosynthetic Process (GO:0050665)
Isoleucine Metabolic Process (GO:0006549)
Kidney Morphogenesis (GO:0060993)
Positive Regulation Of Endothelial Cell Migration (GO:001059...
Regulation Of Endothelial Cell Migration (GO:0010594)
Neuropeptide Signaling Pathway (GO:0007218)
Mesenchyme Development (GO:0060485)
Positive Regulation Of Cartilage Development (GO:0061036)
Substrate-Dependent Cell Migration, Cell Extension (GO:00069...
Response To Organophosphorus (GO:0046683)
Positive Regulation Of CREB Transcription Factor Activity (G...
Superoxide Metabolic Process (GO:0006801)
Enriched terms
Gene count
0.0 2.5 5.0 7.5 10.0
P value
0.0025
0.0050
0.0075
0.0100
E
piRNA Processing (GO:0034587)
Peptidyl-Serine Phosphorylation (GO:0018105)
Peptidyl-Serine Modification (GO:0018209)
Negative Regulation Of RNA Metabolic Process (GO:0051253)
Protein Phosphorylation (GO:0006468)
Positive Regulation Of Adaptive Immune Response Based On Som...
Negative Regulation Of Response To Type II Interferon (GO:00...
Negative Regulation Of Type II Interferon-Mediated Signaling...
Protein Localization To Endoplasmic Reticulum Exit Site (GO:...
Regulatory ncRNA Processing (GO:0070918)
Cilium-Dependent Cell Motility (GO:0060285)
Long-Term Memory (GO:0007616)
Regulation Of Cilium Beat Frequency (GO:0003356)
NADH Metabolic Process (GO:0006734)
Methionine Metabolic Process (GO:0006555)
Multi-Ciliated Epithelial Cell Differentiation (GO:1903251)
Purine Ribonucleoside Monophosphate Catabolic Process (GO:00...
Phosphorylation (GO:0016310)
Regulation Of Dendritic Spine Development (GO:0060998)
Positive Regulation Of Dendritic Spine Development (GO:00609...
Enriched terms
Gene count
0 5 10 15 20
P value
0.003
0.006
0.009

Table S1: Full Neuropathology findings in the operative tissue specimens

**Patient A**
In many areas cortical neuronal lamination appears normal without significant neuronal loss or gliosis. In one area however, NeuN immunohistochemical staining shows slightly disrupted neuronal lamination with scattered dysmorphic and irregularly clustered neurons. Occasional cells with the morphology suggestive of balloon cells are seen (not definite, and not resolved by immunohistochemistry). A neoplastic infiltrate is not identified and mitoses, Rosenthal fibres, eosinophilic granular bodies, calcification are not seen. Features are consistent with malformation of cortical development, Focal Cortical Dysplasia type IIA.
**Patient B**
On microscopic examination of the temporal pole cortical grey matter, subcortical white matter and overlying leptomeninges, there are several foci of leptomeningeal mixed inflammatory infiltrate composed of lymphocytes, macrophages and granulocytes including eosinophils. Subjacent superficial cortical subacute infarction (pallor and dissolution of normal tissue architecture, acute eosinophilic neuronal necrosis and prominence of capillary blood vessels) is consistent with SEEG insertion site.<br>Elsewhere there is prominent Chaslin's subpial gliosis. Where optimally orientated, cortical neurology lamination appears normal without significant neuronal loss or gliosis. Dysmorphic or malorientated neurons not seen, and balloon cells are not identified. There is no evidence of parenchymal inflammation or mineralisation. The boundary between grey and white matter is well defined. Heterotopic white matter neurons are not present to excess. There is a mild increase in subcortical white matter cellularity, interpreted as a degree of non-neoplastic oligodendrocyte hyperplasia.<br>The conclusion is Chaslin's subpial gliosis, with no evidence of focal cortical dysplasia and no diagnostic features of hippocampal sclerosis. There is no evidence of inflammation, vascular malformation or neoplasm.
**Patient C**
**Right frontal biopsy (six years prior to SEEG)**<br>Perivascular and parenchymal lymphocytic inflammation is present and mild to moderate in intensity. Mononuclear inflammatory infiltrate surrounding leptomeningeal vessels is also noted. Vessel wall infiltration by lymphocytes, fibrinoid necrosis or thrombosis, is not identified. Microglial activation is striking, and multiple microglial nodules are identified throughout the grey matter. Small lymphocytes and microglia are focally closely apposed to neurons, but definite neuronophagia is not seen. Subpial gliosis is moderate and extends into cortex and subcortical white matter. Perivascular pigment and macrophages are noted. CD3 highlights large number of T-lymphocytes within the leptomeninges, perivascular spaces and parenchymal brain tissues, as do CD4 and CD8, the former also highlighting very large numbers of microglia. The conclusion is of chronic encephalitis with T-cell rich perivascular and parenchymal inflammation, microglial nodules and widespread microglial activation, subpial and parenchymal gliosis.<br><br>**SEEG resection specimen**<br>In the lateral temporal pole, a significant inflammatory infiltrate is not seen. Microglial nodules are not identified. There is a single foci of perivascular chronic inflammation. In one

specimen there is a linear transversely orientated focus of cortical gliosis and rarefaction without amounting to insipient parenchymal cavitation. In one specimen there is occasional leptomeningeal and perivascular lymphocytes, and rare parenchymal lymphocytes.
In the hippocampal head, there is depletion of and dispersion of granular neurons and pyramidal neurons. In the frontal lobe there are occasional foci of perivascular lymphocytic inflammation. A diffuse parenchymal inflammatory infiltrate or microglial nodules are not seen.
The conclusion is that minimal inflammation in present in the current specimen, restricted largely to perivascular aggregates of T-lymphocytes. Significant parenchymal inflammation is not seen. There are however foci of cortical gliosis and evidence of severe hippocampal sclerosis with aberrant mossy fibre sprouting patterns.

LEGEND OF SUPPLEMENTARY TABLES

Table S1: Full Neuropathology findings in the operative tissue specimens

Table S2. Nanodrop quantifications for DNA isolated from SEEG electrodes.

Table S3. Nanodrop quantifications for RNA isolated from SEEG electrodes.

Table S4. DNA QC before library preparation.

Table S5. RNA QC before library preparation.

Table S6. RNA QC after library preparation.

Table S7. Up regulated genes in FCD PZ vs NIZ comparison.

Table S8. Down regulated genes in FCD PZ vs NIZ comparison.

Table S9. Enriched GO terms in up regulated genes in FCD PZ vs NIZ comparison.

Table S10. Enriched GO terms in down regulated genes in FCD PZ vs NIZ comparison.

Table S11. Up regulated genes in TLE PZ vs NIZ comparison.

Table S12. Down regulated genes in TLE PZ vs NIZ comparison.

Table S13. Enriched GO terms in up regulated genes in TLE PZ vs NIZ comparison.

Table S14. Enriched GO terms in down regulated genes in TLE PZ vs NIZ comparison.

Table S15. Up regulated genes in TLE SOZ vs NIZ comparison.

Table S16. Down regulated genes in TLE SOZ vs NIZ comparison.

Table S17. Enriched GO terms in up regulated genes in TLE SOZ vs NIZ comparison.

Table S18. Enriched GO terms in down regulated genes in TLE SOZ vs NIZ comparison.

Table S19. Up regulated genes in TLE SOZ vs PZ comparison.

Table S20. Down regulated genes in TLE SOZ vs PZ comparison.

Table S21. Enriched GO terms in up regulated genes in TLE SOZ vs PZ comparison.

Table S22. Enriched GO terms in down regulated genes in TLE SOZ vs PZ comparison.

Table S23. Up regulated genes in RE PZ vs NIZ comparison.

Table S24. Down regulated genes in RE PZ vs NIZ comparison.

Table S25. Enriched GO terms in up regulated genes in RE PZ vs NIZ comparison.

Table S26. Enriched GO terms in down regulated genes in RE PZ vs NIZ comparison.

Table S27. Up regulated genes in RE SOZ vs PZ comparison.

Table S28. Down regulated genes in RE SOZ vs PZ comparison.

Table S29. Enriched GO terms in up regulated genes in RE SOZ vs PZ comparison.

Table S30. Enriched GO terms in downregulated genes in RE SOZ vs PZ comparison.

Table S31. Up regulated genes in RE SOZ vs NIZ comparison.

Table S32. Down regulated genes in RE SOZ vs NIZ comparison.

Table S33. Enriched GO terms in up regulated genes in RE SOZ vs NIZ comparison.

Table S34. Enriched GO terms in down regulated genes in RE SOZ vs NIZ comparison.

LEGENDS FOR SUPPLEMENTARY FIGURES

Figure S1. Neuropathology details of study participants. (A) NeuN (MilliporeSigma, USA) immunohistochemical staining for patient A demonstrating dysmorphic, haphazardly arranged and abnormally clustered cortical neurons consistent with Focal Cortical Dysplasia, ILAE Type 2A. No clear balloon cells were identified. (B) Hematoxylin and eosin (H&E) staining for patient B showing prominent Chaslin's subpial gliosis. Cortical neurology lamination appears normal without significant neuronal loss or gliosis. Dysmorphic or malorientated neurons not seen, and balloon cells are not identified. Heterotopic white matter neurons are not present to excess. There is a mild increase in subcortical white matter cellularity, interpreted as a degree of non-neoplastic oligodendrocyte hyperplasia. H&E (C), NeuN (D) and Zinc transporter 3 (Proteintech, USA) (E) staining for patient C showing severe hippocampal sclerosis with granular neuron depletion and mossy fibre sprouting. In the hippocampal head, there is depletion of and dispersion of granular neurons and pyramidal neurons. CD3 (Agilent Dako, US) (F) and CD68 (Agilent Dako, USA) (G) staining for patient C highlighting the presence of T-lymphocytes and macrophages respectively. Minimal inflammation in present in the current specimen, restricted largely to perivascular aggregates of T-lymphocytes. Significant parenchymal inflammation is not seen.

Figure S2. Nucleic acid extraction from SEEG electrodes from epilepsy patients. (A). Trypan blue staining shows the presence of cells in the SEEG electrodes from epilepsy patients. (B). The amount of total nuclear fractions was directly proportional to a number of cut pieces (Metal contact points) from one electrode. (C). High sensitivity fragment analyser shows the presence of both DNA

and RNA in the samples (S1 and S2). (D-E). Electropherograms from the fragment analyzer confirm the RNA and DNA with their respective molecular weight. (F). Electropherograms from the fragment analyzer confirm the effective separation of DNA from total nucleic acid fraction. (G). Tape station analysis of purified RNA fraction confirmed the quality of (RIN) extracted RNA.

Figure S3. Quality check of the transcriptome datasets. (A) The distribution of RNA integrity number (RIN) across all samples from the NIZ, PZ, and SOZ regions of FCD, TLE, and RE brains. The plot illustrates the variability in RNA quality within each region and across different brain conditions.(B) A heatmap depicting the consistent expression of genes across all replicates of FCD brain samples, regardless of gene length and the RIN values of the samples. The heatmap highlights the robustness of gene expression patterns in FCD brains.

Figure S4. Quality assessment for TLE and RE brain transcriptomes. (A) Heatmap illustrating uniform gene expression across all TLE brain sample replicates, indicating stable expression profiles independent of gene length and RIN values. (B) Heatmap depicting consistent gene expression across all RE brain sample replicates, similarly indicating reliable expression patterns regardless of gene length and RIN values.

Figure S5. Reproducibility of electrode transcriptome in tissue samples from matched brain regions with similar seizure activity. (A-D) The transcriptome from electrodes and corresponding tissue samples from TLE patients in SOZ region shows a strong correlation (correlation coefficient: 0.7). The correlation was evaluated at various FPKM thresholds to assess the consistency of results.

Figure S6. Enrichment of differentially expressed genes (DEGs) identified in our study in publicly available scRNA-seq and bulk RNA-seq datasets. (A) UMAP shows the integration of Epilepsy (TLE) and control (healthy) scRNA and (B) their cell type annotation using the (C) marker

expression in violoin plot. (D) Dotplot shows the enrichment of our DEGs in previously reported TLE and RE bulk and single cell transcriptome (RNA-seq).

Figure S7. Gene expression differences between NIZ and PZ areas of FCD brains. (A) Replicate-wise correlation of NIZ and PZ regions transcriptome. (B) Volcano plot of differentially expressed genes between PZ and NIZ regions. (C-D) GO enrichment of up and down regulated genes, respectively. (E) Heatmap of down regulated known epilepsy risk genes in PZ as compared with NIZ region. (F) Dotplot showing the expression of down regulated genes (PZ vs NIZ) in Epilepsy vs Healthy scRNA data.

Figure S8. Gene expression differences between NIZ and PZ areas of TLE brains. This figure examines gene expression changes in different regions of non-lesional temporal lobe epilepsy (TLE) brains. Panels (A-B) show GO enrichment for upregulated and downregulated genes in the PZ vs. NIZ comparison in TLE brains, respectively. (C-D) Display the replicate-wise correlation of NIZ and SOZ regions and a volcano plot of differentially expressed genes for these regions. (F-G) Present GO enrichment for upregulated and downregulated genes in the SOZ vs. NIZ comparison in TLE brains, respectively. (E) Dot plot showing the expression of upregulated genes in SOZ vs. NIZ and Epilepsy vs. Healthy samples from public data.

Figure S9. Transcriptome response in PZ and SOZ areas of TLE and RE brains. This figure explores gene expression changes in the PZ and SOZ regions of TLE and RE brains. Panels (A-B) show GO enrichment analysis of upregulated and downregulated genes in the SOZ vs. PZ comparison within the TLE brain. (C-D) Present replicate-wise correlations and differential gene expression analysis between the SOZ and PZ regions in the RE brain. Panels (E-F) display GO enrichment analysis of upregulated and downregulated genes in the SOZ vs. PZ comparison within the RE brain.

Figure S10. RE transcriptome response in NIZ, PZ and SOZ regions. (A-B) GO enrichment of up- and down regulated genes in PZ vs NIZ region comparison of RE brain. (C-D) Replicate- wise correlations and differential expression of genes in SOZ and NIZ regions of RE brain. (E and G) GO enrichment of up- and down regulated genes in SOZ vs NIZ region comparison of RE brain. (F) Dotplot showing the expression of up regulated genes (SOZ vs NIZ) in Epilepsy vs Healthy scRNA from public data.

Figure S11. Enrichment of differentially methylated genes (DMGs) identified in our study in publicly available bulk methylome data and DNA methylome landscape in FCD. (A-B) Dotplot shows the enrichment of our DMGs in previously reported drug resistants TLE (DR TLE) and Mesial TLE (MTLE) methylome. (C-D) Transcriptome and methylome density plot in gene body and flanking regions. (E) Replicated wise correlation plot of PZ and NIZ regions methylome in FCD brain. (F) A comparable number of hypermethylated (216) and hypomethylated (1801) DMRs are identified when comparing the PZ to the NIZ region. (G-H) GO enrichment of hypomethylated and hypermethylated genes found in PZ regions.

Figure S12. Transcriptome and methylome comparison for TLE brain. (A) Heatmap of down regulated and hyper methylated genes in PZ vs NIZ comparison. (B-C) GO enrichment of hypomethylated and hypermethylated genes in PZ vs NIZ regions. (D) Replicate-wise correlation plot of SOZ and NIZ regions methylome in TLE brain. (E) A comparable number of hypermethylated (636) and hypomethylated (475) DMRs are identified when comparing the SOZ to the NIZ region. (F-G) GO enrichment of hypomethylated and hypermethylated genes found in SOZ vs NIZ regions.

Figure S13. Transcriptome and methylome analogy in SOZ and PZ regions in TLE brain. (A-B) Transcriptome and methylome density plot in gene body and flanking regions. (C) Replicated wise correlation plot of SOZ and PZ regions methylome in TLE brain. (D) A comparable number of

hypermethylated (608) and hypomethylated (872) DMRs are identified when comparing the SOZ to the PZ region. (E-F) GO enrichment of hypomethylated and hypermethylated genes found in SOZ vs PZ regions.

Figure S14. Transcriptome and methylome landscape in SOZ and NIZ regions in RE brain. (A-B) Transcriptome and methylome density plot in gene body and flanking regions. (C) Replicated wise correlation plot of PZ and NIZ regions methylome in RE brain. (D) A comparable number of hypermethylated (31) and hypomethylated (328) DMRs are identified when comparing the PZ to the NIZ region. (E) Up regulated and hypo methylated genes in PZ as compared to NIZ regions. (F) GO enrichment of hypomethylated and hypermethylated genes found in PZ vs NIZ regions.

Figure S15. An attempt to identify short variants (SNPs + Indels) from transcriptome (RNA-seq) data in TLE and RE brains. (A) We identified short variants using GATK best practices workflows for RNAseq short variant discovery (SNPs + Indels) and annotated the high quality variants through wANNOVAR. (B-C). Chromosome wise distribution of variants in RE (NIZ and PZ) and TLE (NIZ, PZ and SOZ) brains. (D) Number of variants falling in different categories in TLE and RE brains. (E) Functional annotations of variants harbouring at different genomic regions.

Figure S16. Functional enrichment analysis of variants in NIZ, PZ, and SOZ regions of TLE brain. (A-B) Venn diagrams depicting the overlap between genes with variants in the NIZ, PZ, and SOZ regions of TLE brains and those associated with epilepsy risk. (C) GO enrichment analysis of genes carrying variants across all three regions (NIZ, PZ, and SOZ). GO enrichment specific to genes with variants found exclusively in the (D) SOZ, (E) PZ, and (F) NIZ regions.

Figure S17. Functional enrichment analysis of variants in NIZ and PZ regions of RE brains. (A-B) Venn diagrams illustrating the intersection of genes with variants in the NIZ and PZ regions of RE brains and known epilepsy risk genes. (C) GO enrichment analysis for genes containing variants

across NIZ and PZ regions. GO enrichment results for genes with variants specific to the (D) PZ and (E) NIZ regions.
